# Iron–Nitride Isomerization as a Deactivation Pathway in Ammonia Oxidation: A Computational Study of Sequential HAT Mechanisms

**DOI:** 10.1021/acs.inorgchem.6c02749

**Published:** 2026-10-13

**Authors:** Luo Sheng, Julianna Oláh

**Affiliations:** Department of Inorganic and Analytical Chemistry, Faculty of Chemical Technology and Biotechnology, Budapest University of Technology and Economics, Műegyetem Rkp. 3, Budapest 1111, Hungary

## Abstract

Ammonia oxidation remains a kinetic bottleneck in utilizing ammonia as a carbon-free energy carrier. Iron-based complexes offer a cost-effective alternative to noble-metal catalysts, but use is hampered by low turnover numbers. Here, density functional theory is used to investigate the catalytic activity and deactivation pathways of Fe^2+^/Fe^3+^ complexes bearing cis-ammonia ligands. We confirm the ground-state electronic structures of these complexes, then establish an accurate SMD/DRACO protocol—using a van der Waals cavity with Gaussian-smeared surface charge discretization—that predicts redox potentials with mean error below 0.013 V versus experiment. Thermodynamic evaluation of sequential hydrogen atom transfer (HAT) steps reveals a “peaked” bond dissociation free energy profile. Crucially, the exergonic third HAT step yields not the anticipated nitrido species, but a novel isomer in which the nitride inserts into the Fe–C bond. This Fe–N–C isomer is a deep thermodynamic sink, trapping the catalyst in an inactive state. A similarly low-energy Fe–N–C isomer exists for the imido intermediate but is kinetically inaccessible. These results indicate that Fe–C-containing complexes are unlikely to serve as efficient AO catalysts, as a high-valent iron center with a multiply bonded nitrogen ligand renders the Fe–C bond prone to alkyl migration, a facile deactivation pathway.

## Graphical abstract

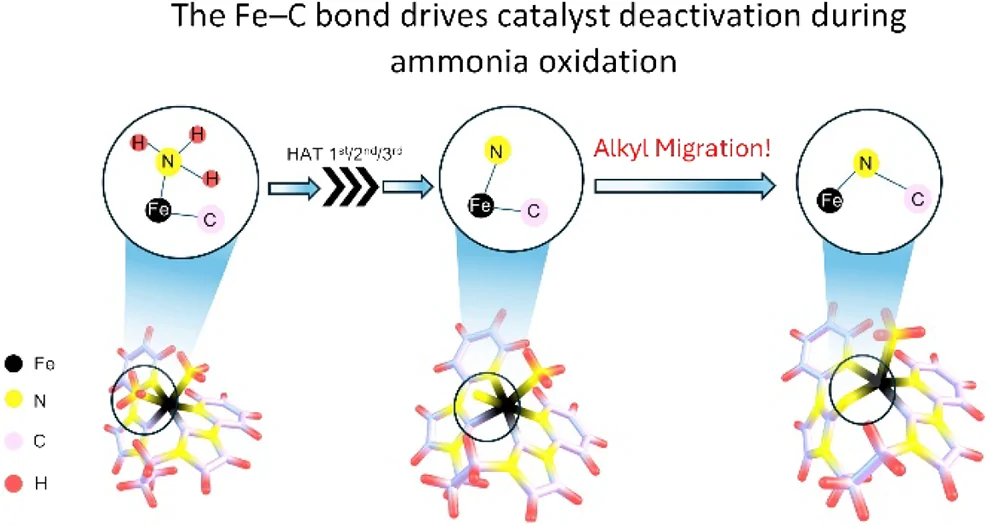

## 1 Introduction

Ammonia (NH_3_), as a pivotal nitrogen-containing compound, plays an essential role in both the global nitrogen cycle and various industrial applications. Since the development of the Haber–Bosch process, ammonia has served as a fundamental platform molecule for the production of nitrogen fertilizers, chemical intermediates, and high-energy materials. In recent years, driven by the global pursuit of carbon neutrality and the transformation of energy systems, ammonia is increasingly being recognized as a promising carbon-free energy carrier^1,2^ and hydrogen resource which can be used in hydrogen fuel cells.

However, the practical implementation of an “Ammonia Economy” faces several challenges (Figure 1). First, while atmospheric nitrogen (*N*_2_) provides an inexhaustible feedstock, its industrial fixation via the conventional Haber–Bosch process remains remarkably energy-intensive, requiring high temperatures (400–500 °C) and pressures (150–250 bar), hereby contributing significantly to global CO_2_ emissions.^1,3^ Second, the high toxicity and corrosive nature of ammonia necessitate advanced materials and rigorous protocols for its safe storage and distribution.^4,5^ Finally, the efficient extraction of energy or high-purity hydrogen from ammonia is often bottlenecked by the sluggish kinetics of the ammonia oxidation reaction and the high overpotentials associated with its decomposition.^6,7^

**Figure 1:**
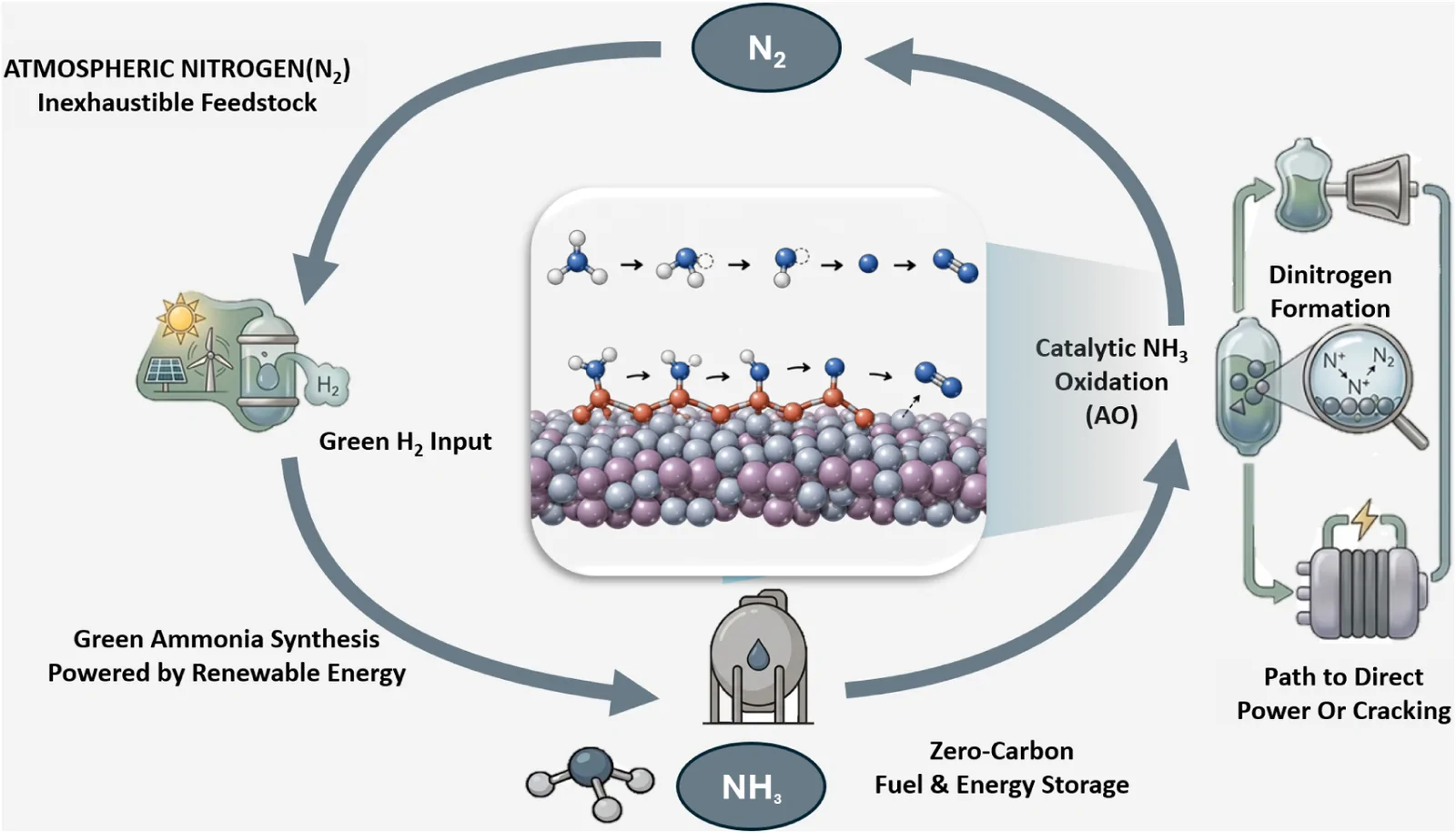
Vision of the “Ammonia Economy” cycle.

To address these limitations and transition toward a truly sustainable energy resource, researchers are increasingly focusing on the development of molecular transition-metal complexes as catalysts.^8,9^

Considerable research has been done on the development of catalysts for N_2_ reduction to NH_3_ in order to find an alternative route to the Haber–Bosch process. Until now, the most efficient molecular catalysts have been proposed by the groups of Peters^10^ and Nishibayasi.^12–13^ Unfortunately, the turnover number of these catalysts has not yet reached the level required for an industrially applicable catalyst. In our previous computational works, we considered various facets of N_2_ reduction, including the mechanism of the main^14^ and side reactions,^13^ the catalyst deactivation mechanism via an autocatalytic hydrogen evolution reaction,^11^ and potential routes for improving performance.^15^ However, not only ammonia production but also its storage is a crucial factor for establishing ammonia as a carbon-free energy resource.^16^

The present work focuses specifically on the catalytic oxidation of ammonia; the overall stoichiometry for the electrochemical six-electron, six-proton oxidation of ammonia is shown in eq 1:

1 $$2NH_{3}\to N_{2}+6e^{-}+6H^{+}$$

Scientific exploration of this reaction dates back to 1788, when French chemist Claude Louis Berthollet investigated the oxidation of ammonia using manganese dioxide. The primary motivation for these seminal investigations was to elucidate the chemical pathways of the nitrogen cycle and to seek alternative routes for the synthesis of nitric acid—a vital precursor for industrial saltpeter production.

Over the subsequent centuries, research has predominantly centered on developing efficient heterogeneous catalysts, particularly noble-metal-based systems (e.g., Pt, Ru, and Rh),^9,17,18^ utilized in industrial processes such as the Ostwald process. Despite their superior catalytic activity, these precious metals are not only prohibitively expensive but typically require harsh operating conditions and remain highly susceptible to deactivation via surface deactivation.^19^ Consequently, the burgeoning interest in electrocatalytic ammonia oxidation has made a strategic shift toward earth-abundant transition metals (e.g., Fe, Ni, Co).^20–23^ While these nonprecious metal systems offer significant cost advantages and scalability, they often grapple with challenges regarding poor selectivity, limited durability, or potential environmental toxicity. Therefore, the development of selective, durable, and environmentally benign catalysts based on abundant transition metals has emerged as a critical objective for realizing a sustainable and circular ammonia economy.

Driven by this trend, cost-effective, environmentally compatible, and redox-flexible iron-based systems have garnered significant attention for catalyzing the ammonia oxidation reaction under both thermal and electrochemical conditions. Moreover, recent studies have demonstrated that tailoring the coordination environment and oxidation state of iron can significantly influence its catalytic activity and selectivity, offering new avenues for designing efficient non-noble-metal ammonia oxidation catalysts.^10^ Despite these promising developments, the turnover number (TON) of most reported catalysts remains relatively low^10^ and far below the threshold required for practical or industrial applications.

Beyond ammonia oxidation specifically, recent studies have underscored how spin-state control and ligand design govern the efficiency of iron-mediated multielectron and multiproton transformations more broadly. Lee and coworkers demonstrated that a low-spin Fe­(II) complex bearing a redox-active imino-bipyridyl ligand undergoes rapid, ligand-centered two-electron transfer to achieve an exceptionally high turnover frequency for hydrogen evolution, whereas an analogous high-spin complex exhibits markedly slower electron transfer and correspondingly lower activity.^24^ This highlights that spin state—rather than metal oxidation state alone—can dictate catalytic performance. Complementary strategies incorporating proton-relay functionalities into the secondary coordination sphere have also proven effective for multiproton, multielectron catalysis. Kumar et al. reported a μ-oxo-bridged diiron complex in which an uncoordinated thiazolinium moiety and the bridging oxo group cooperatively shuttle protons during electrocatalytic hydrogen evolution, with the operative ECEC or EECC pathway governed by the strength of the exogenous acid.^25^ On the computational side, high-throughput screening of iron-based dual-atom catalysts has recently been used to identify electronic descriptors that minimize the limiting potential for nitrogen reduction to ammonia.^26^ These studies collectively illustrate that spin-state effects, redox-active/proton-responsive ligand design, and descriptor-based computational screening are emerging as central themes in the rational design of iron catalysts for small-molecule activation, providing a broader mechanistic context for the spin-crossover-mediated deactivation pathway identified in the present work.

In this context, Phearman and Bullock used thermodynamic predictions to design iron-based catalysts with ammonia ligand in *cis*-orientation (see Figure 2) but the complexes were unable to catalyze NH_3_ oxidation to N_2_ under typical electrochemical conditions, although the application of ^t^Bu_3_ArO• (2,4,6-Tri*tert*-butylphenoxyl) radical to abstract hydrogen atoms from NH_3_ led to the formation of a small amount of N_2_.^27^

**Figure 2:**
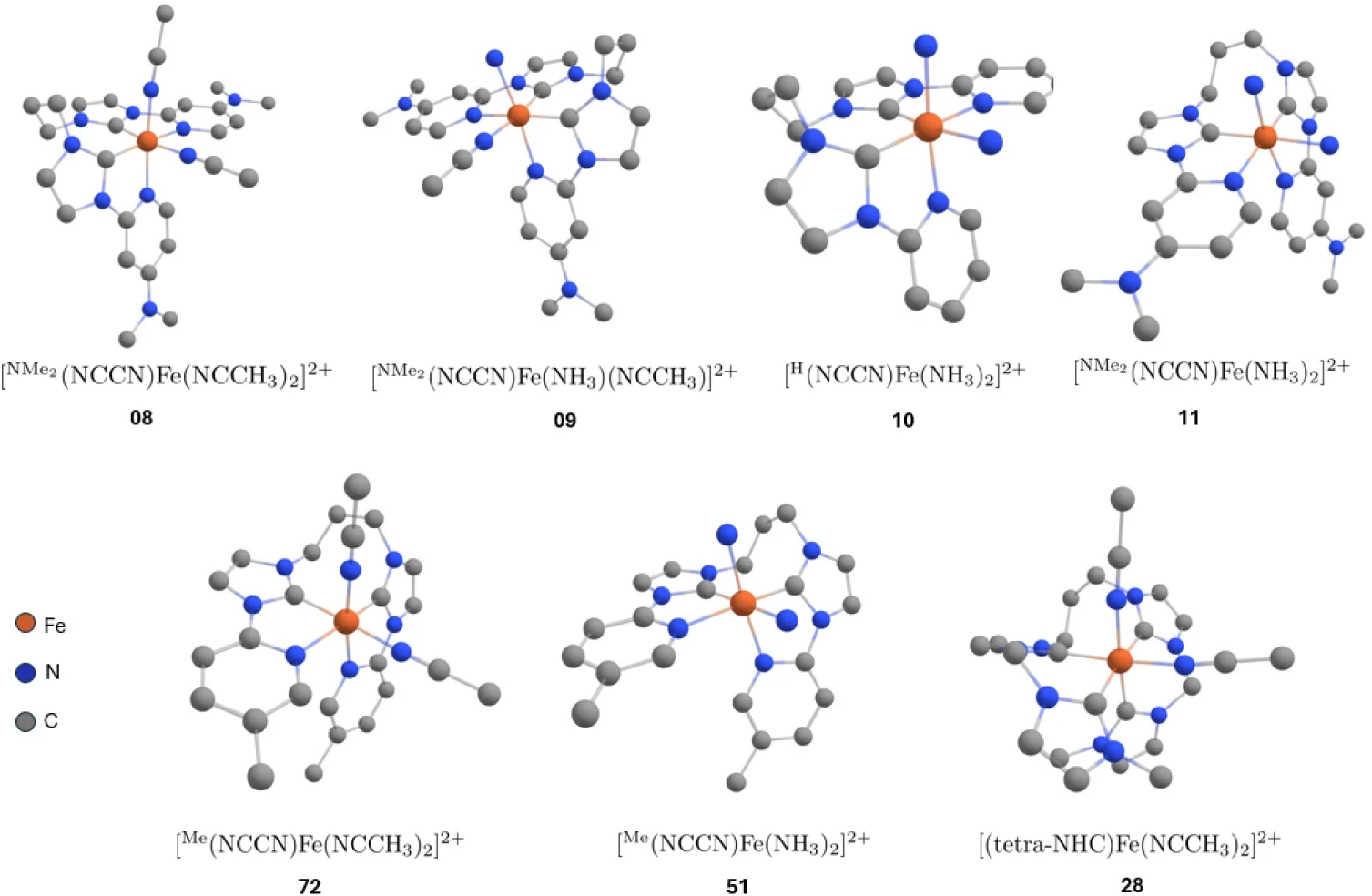
Fe complexes synthesized by Phearman and Bullock.^27^ Hydrogen atoms are omitted for clarity.

This apparent inconsistency between theory and experiment has prompted a closer examination of the intrinsic factors governing catalytic activity. Systematic investigations indicate that the performance is not straightforwardly correlated with the Fe^2+^/Fe^3+^ potential alone. Instead, subtle variations in the oxidation state, spin configuration, and ligand field geometry can dramatically alter activation barriers and the stability of potential intermediates, leading to divergent mechanistic outcomes.^9,12^

Given these complexities, computational chemistry provides an indispensable framework for complementing experimental observations. In particular, density functional theory (DFT) allows for the detailed investigation of the structure and reactivity of transient intermediates that are often too short-lived for experimental detection.^28^ By mapping the reaction energy landscape and evaluating the activation barriers of key elementary steps, computational methods offer a toolbox to gain insights into the fundamental bottlenecks of the ammonia oxidation reaction.

In this work, we study in detail the properties of the complexes (see Figure 2) synthesized by Phearman and Bullock.^27^ Following their established nomenclature, the complexes are identified by the last two digits of their CCDC identifiers (e.g., “***08***” for 2271708)

We systematically evaluate their spin-state energetics, redox potential, and ability to undergo hydrogen atom abstraction to yield N_2_ and suggest a potential catalyst deactivation route, to explain their observed poor catalytic performance. Our work may contribute to the development of more efficient, non-noble-metal catalysts with improved performance.

## 2 Methods

### 2.1 Computational Details

Computational analyses were performed on a series of iron complexes (see Figure 2) originally reported by Phearman and Bullock^27^ using the ORCA 6 software package.^29,30^ Crystallographic structures served as the starting point for geometry optimizations and subsequent frequency calculations, which were conducted at the PBE/def2-TZVP­(-f)^31,32^ level of theory. To ensure a rigorous description of the potential energy surface, the geometries of both Fe^2+^ and Fe^3+^ complexes were optimized in all relevant spin states to identify their ground states.

To refine the electronic energies and assess functional sensitivity, single-point calculations were performed in the gas phase using the def2-TZVP basis set. Seven density functionals representing different rungs of Jacob’s Ladder were systematically used: PBE (Generalized Gradient Approximation, GGA); TPSSh^33^ (meta-GGA); B3LYP*, B3LYP,^34^ and ωB97X^35^ (Hybrids); and PWPB95 and B2PLYP^36^ (Double-Hybrid).

In all calculations, the D3 dispersion correction^37^ with Becke-Johnson (BJ) damping^38^ and the resolution of identity (RI) approximation^39^ were employed to enhance both accuracy and computational efficiency.

Solvation free energies (Δ*G*_sol_) were calculated at the gas-phase geometries in acetonitrile to align with the experimental conditions, which is a validated protocol for transition metal complexes.^40,41^ Following the original SMD^42^ parametrization, Δ*G*_sol_ values were determined at the M05–2*X*/6–31G* ^43,44^ level, utilizing the LibXC implementation of the functional within ORCA 6.0. To evaluate the influence of solvation cavity parameters, we investigated the point charge (GEPOL-SES, GEPOL-SAS) and Gaussian charge (vdW cavity) surface representation in conjunction with and without the DRACO (Dynamic Radii Adjustment for Continuum Solvation) method. The SMD/DRACO^42,45^ protocol was ultimately selected for all final redox potential predictions, as it yielded the highest accuracy relative to the experimental benchmarks. Geometries are provided in the Supporting Information (SI).

### 2.2 Redox Potential Calculations

Solution-phase redox potentials (*E*_1/2_) were determined by evaluating the free energy changes associated with the reduction half-reactions. First, the total Gibbs free energy of a species in solution $\left(G_{\mathrm{sol}}^{*}\right)$ was calculated according to eq 1:

2 $$G_{\mathrm{sol}}^{*}=G_{\mathrm{therm}}+\Delta G_{\mathrm{sol}}+E_{\mathrm{el}}+\Delta G^{1\mathrm{atm}\to 1M}$$

Where:

- *E*_el_ is the single-point electronic energy obtained from gas-phase calculations.
- *G*_therm_ represents the thermal correction to the Gibbs free energy calculated in the gas phase (accounting for translational, rotational, and vibrational contributions at 298.15 K).
- Δ*G*_sol_ is the solvation free energy determined via the SMD protocol.
- Δ*G* ^1atm→1M^ is the standard-state correction term (kcal/mol at 298.15 K), which accounts for the volume change from the gas phase (atm) to the solution phase (M).

Subsequently, the Gibbs free energy change of the half-cell reaction was determined (eq 3) by calculating the difference in $G_{sol}^{*}$ between the reduced and oxidized species.

3 $$\Delta G_{\mathrm{sol}}^{*}=G_{\mathrm{sol},\mathrm{ox}}^{*}-G_{\mathrm{sol},\mathrm{re}}^{*}$$

To facilitate a direct comparison with experimental data, the calculated redox potentials were converted to a relative scale using the ferrocene/ferrocenium (Fc/Fc^+^) redox couple as the formal reference electrode. To ensure systematic error cancellation, the absolute potential of the reference electrode $\left(E_{ref}^{0}\right)$ was determined using the identical computational protocol as applied to the complexes. Based on the Nernst equation, the relative redox potential was derived using the following relationship (eq 4):

4 $$E^{0}=\frac{\Delta G_{\mathrm{sol}}^{*}}{nF}-E_{ref}^{0}$$

### 2.3 Bond Dissociation Free Energy

The N–H bond dissociation free energy (BDFE) was evaluated across the gas phase (vacuum), and the HAT reaction energy was evaluated in the presence of two reference radicals, TEMPO• and ^t^Bu_3_ArO•. To ensure energetic consistency and minimize systematic errors, the BDFE values for the HAT partners, TEMPO–H and ^t^Bu_3_ArO–H, were computed at the same level of theory as the parent species. The BDFE was then derived from the calculated Gibbs free energy change of the homolytic cleavage (eq 5) or the corresponding HAT reaction (eq 6).

5 $$\mathrm{Bond dissociation free energy}:{\mathrm{Fe‐NH}}_{x}\to Fe{\mathrm{‐NH}}_{x-1}^{\cdot }+H^{\cdot }$$

6 $$\mathrm{Hydrogen atom transfer}:Fe{\mathrm{‐NH}}_{x}+R^{\cdot }\to Fe{\mathrm{‐NH}}_{x-1}^{\cdot }+\mathrm{RH}$$

Where R is TEMPO• or ^t^Bu_3_ArO•, *x* = 1–3. And Fe indicates the whole complex without the NH_3_ ligand.

## 3 Results and Discussion

This study first examines the electronic spin states of the investigated complexes, followed by the establishment of a robust computational protocol for the accurate estimation of their redox potentials. Subsequently, the facility of hydrogen atom abstraction is evaluated through the calculation of bond dissociation free energies (BDFEs) and HAT reactions. Finally, we propose a putative deactivation mechanism to elucidate the structural degradation of the catalysts, providing a rationale for their observed experimental inactivities.

### 3.1 Spin-State Energetics and Electronic Structure

The catalytic performance of transition-metal complexes is intrinsically linked to the spin state of the metal center, a cornerstone of spin catalysis^46^ that governs the N_2_ evolution processes. Therefore, we systematically assessed the energetics of all accessible spin states: multiplicities (*M*) = 1,3,5 were considered for Fe^2+^ and *M* = 2,4,6 for the corresponding Fe^3+^ species.

Our calculations unambiguously identify the low-spin (LS) configuration—singlet (*M* = 1) for Fe^2+^ and doublet (*M* = 2) for Fe^3+^—as the ground state, independent of the employed density functional. The data highlight a pronounced sensitivity of the spin-state splitting to the Hartree–Fock exchange fraction in accordance with the established phenomenon that an increased HF admixture systematically leads to the overstabilization of the high-spin (HS) and intermediate-spin (IS) states as exact exchange inherently favors parallel-spin electrons.^47^

Quantitatively, the pure GGA functional (PBE) strongly favors the LS configuration (see Figure 3 for complex ***09*** and Table S1 in the SI for all numerical data), placing the HS state 30–40 kcal mol^–1^ higher in energy. The incorporation of exact exchange via hybrid functionals (e.g., TPSSh, B3LYP*, B3LYP, and ωB97X-D) narrows this HS–LS energy gap to a more moderate 10–20 kcal mol^–1^. Further inclusion of perturbative correlation effects via double-hybrid functionals (e.g., PWPB95 and B2PLYP) slightly refines the energetics while strictly preserving the overall energetic trend. Ultimately, the LS ground state is well-established for both the Fe^2+^ and Fe^3+^ oxidation states.

**Figure 3:**
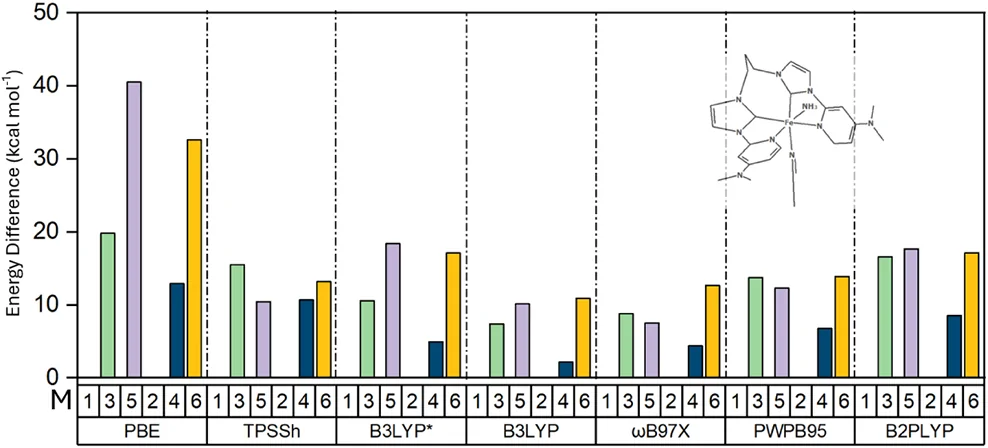
Functional benchmark on different spin states of complex ***09*** with a central Fe^2+^ (*M* = 1,3,5) and Fe^3+^ (*M* = 2,4,6) center.

### 3.2 Redox Potential Calculations: Protocol Development and Error Analysis

Predicting the redox potentials of transition metal complexes, especially Fe^2+^/Fe^3+^ systems, poses significant computational challenges. The primary difficulty lies in the immense absolute magnitude of the solvation free energies involved. For aqueous systems, the absolute hydration free energy of Fe^2+^ is approximately −440 to −450 kcal/mol, while that of Fe^3+^ is about −1040 to −1050 kcal/mol.^48^ The difference reaches about −600 kcal/mol, which nearly cancels the large gas-phase ionization energy (∼700 kcal/mol) and brings the solution-phase redox potential into a measurable range. Critically, an error of only 1 kcal/mol in solvation energy translates into ∼43 mV deviation in the calculated redox potential. For a system with total solvation energies exceeding 1000 kcal/mol, a mere 0.5% model error can thus cause >200 mV error—an unacceptable level for meaningful comparison with experiment.^48^

To navigate these severe accuracy requirements, robust experimental benchmarks are essential. Because all complexes studied here have experimentally measured redox potentials (Table S2 in the SI), they constitute an ideal test set for method development and validation.

Our initial attempts at calculating these potentials yielded significant deviations of up to 1 V (see Table S2 in the SI) from experimental values, confirming that standard protocols may be insufficient for such sensitive systems. As expected, the accurate treatment of the solvation free energy (Δ*G*_solv) is typically the primary bottleneck in redox potential prediction. To address this, we systematically investigated the computational protocol’s sensitivity to two decisive factors: (i) the cavity surface type and charge discretization scheme, and (ii) the performance of various DFT functionals.

#### 3.2.1 Sensitivity to Cavity Surface Type and Charge Discretization

We first benchmarked the redox potential across all six benchmark complexes using several combinations of cavity surface and charge discretization scheme (Table 1). Using the Solvent Accessible Surface (SAS) with the standard point-charge discretization, the calculated potentials showed a mean absolute error (MAE) of 0.890 V—an unacceptably poor result, consistent with the known tendency of the SAS cavity to poorly represent the compact, highly charged coordination environment around Fe centers. Switching to the Solvent Excluded Surface (SES) with point-charge discretization produced a dramatic improvement, reducing the MAE to 0.105 V without the DRACO correction. Incorporating the DRACO correction to refine the atomic radii used to construct the SES cavity further reduced the MAE to 0.032 V—a systematic improvement of 0.073 V (an ∼70% reduction in error) averaged across all six complexes. While this improvement appeared modest for an individual complex in our earlier, more limited comparison, the full six-complex benchmark demonstrates that the DRACO correction provides a substantial and non-negligible improvement for the SES cavity, well outside the scatter expected from the intrinsic DFT functional uncertainty.

**Table 1:** Impact of Cavity Surface Type, Charge Discretization, and DRACO Correction on Calculated Redox Potentials, Averaged over Six Benchmark Complexes^a^

Surface/Charge Scheme Surface Type	DRACO	MAE (V)
GEPOL-SAS, point charge	On	0.890
GEPOL-SES, point charge	Off	0.105
GEPOL-SES, point charge	On	**0.032**
vdW cavity, Gaussian charge	Off	0.028
vdW cavity, Gaussian charge	On	**0.013**

a Gas-phase single-point energies were determined at the PWPB95/def2-TZVP level; solvation free energies were evaluated at the M05–2*X*/6–31G* SMD level. See Table S1 for the complete per-complex breakdown (complexes 08, 09, 10, 72, 51, 28).

We additionally evaluated an alternative charge discretization scheme, in which the induced surface charge is represented using a Gaussian-smeared distribution on a van der Waals (vdW)-based cavity, rather than the discrete point-charge GEPOL tessellation. This Gaussian-charge scheme achieved an MAE of 0.028 V without DRACO and 0.013 V with DRACO—both slightly lower than the corresponding point-charge SES values, and again showing a clear, systematic improvement upon application of the DRACO correction. These results confirm that (a) the choice of cavity surface type is the single most important factor governing the accuracy of the calculated redox potentials for these Fe complexes, and (b) the Gaussian-smeared charge discretization scheme performs at least as well as, and in most cases modestly better than, the conventional point-charge scheme once a physically appropriate (SES/vdW-type) cavity and the DRACO correction are employed. Given this marginal advantage, all subsequent gas-phase functional benchmarking (Table 2) was carried out using the Gaussian-charge/DRACO protocol.

**Table 2:** Absolute Errors of Redox Potentials (V) Calculated with Different DFT Functionals in Conjunction with def2-TZVP Basis Set^a^,^b^

Complex	PBE	B3LYP	B3LYP*	TPSSh	ωB97X	B2PLYP	PWPB95
*08*	0.256	0.178	0.100	0.077	0.121	0.069	0.032
*09*	0.267	0.154	0.063	0.024	0.129	0.005	0.003
*10*	0.128	0.178	0.110	0.034	0.114	0.024	0.011
*72*	0.135	0.158	0.100	0.048	0.070	0.034	0.013
*51*	0.156	0.180	0.106	0.029	0.131	0.019	0.016
*28*	0.080	0.116	0.080	0.064	0.009	0.048	0.006
MAE	0.170	0.161	0.093	0.046	0.096	0.033	0.013

a PBE predicted the redox potential to be smaller than the experimental value in contrast to all other functionals.

b For complex ***51***, the experimental value is reported as a range (0.05–0.10 V); the midpoint (0.075 V) was used for error calculation.

Functional sensitivity. In parallel, we examined the performance of various DFT functionals for the gas-phase energetics, using the Gaussian-charge/DRACO protocol established above (Table 2). GGA-type PBE systematically yielded the largest deviations (MAE = 0.170 V), consistently underestimating the redox potentials relative to experiment (see Table 2, note a), in contrast to all other functionals tested. Hybrid functionals (B3LYP, ωB97X: MAE = 0.161, 0.096 V, respectively) performed markedly better, and the reduced-exchange B3LYP* variant offered a further improvement (MAE = 0.093 V), consistent with the known tendency of standard hybrid functionals to overstabilize higher-spin states in first-row transition-metal complexes. The meta-GGA hybrid TPSSh gave a comparably low MAE (0.046 V). Double-hybrid functionals (B2PLYP, PWPB95) gave the best overall agreement with experiment (MAE = 0.033, 0.013 V, respectively), with PWPB95 yielding the lowest error among all functionals tested.

### 3.3 Thermodynamic and Nitrogen Formation

Let us now turn to the energetic cost of N_2_ formation and a possible catalytic cycle. The reaction mechanism of ammonia oxidation has been recently proposed to follow a stepwise mechanism involving three HAT steps^49^ followed by coupling of the nitrides and regeneration of the catalyst (Scheme 1).

**Scheme 1:**
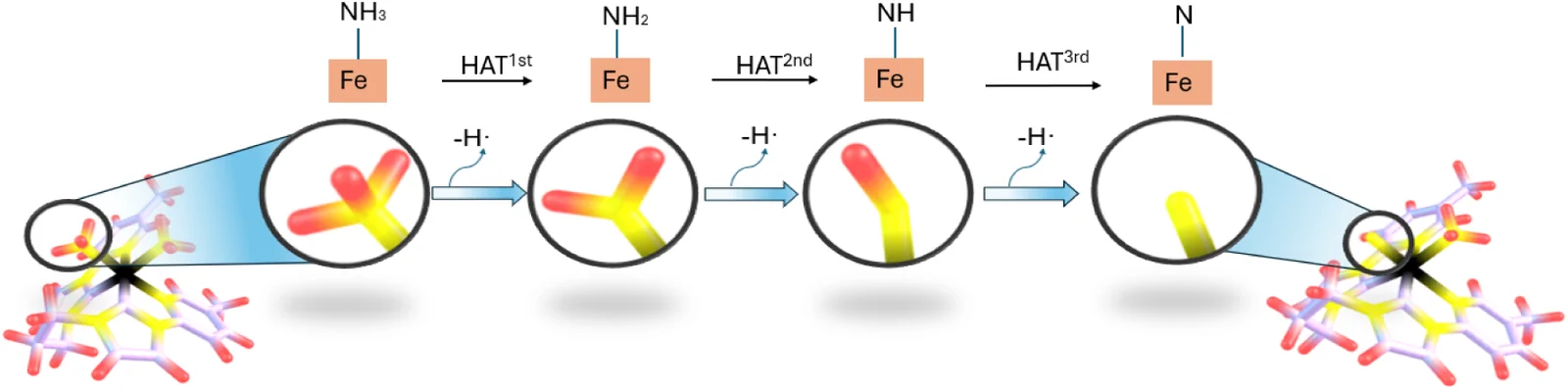
Hydrogen Atom Transfer Step

Two of the investigated complexes (***10*** and ***51***) feature two ammonia ligands in a *cis*-orientation, placing them in close spatial proximity. This specific arrangement raises a critical mechanistic question: is intramolecular dinitrogen production a viable pathway (eq 7), or does the reaction proceed via an intermolecular route involving the coupling of nitrido ligands from two separate mononuclear complexes (eq 8). Might other products involving lower oxidation states of nitrogen, e.g., hydrazine be byproducts (eq 9). To address this, we evaluated the thermodynamics of three pathways, utilizing TEMPO• and ^t^Bu_3_ArO• as hydrogen-abstracting agents.

The intramolecular pathways involve a single metal center where both NH_3_ ligands are activated to form a transient species—such as a bis-nitrido or a hydrazine-like intermediate—that releases N_2_ or H_2_N=NH_2_ from the same complex leading to a tetracoordinated iron complex. Conversely, the intermolecular pathway involves the coupling of two mononuclear metal-nitrido complexes, which are formed after three successive HAT steps; in this case, two pentacoordinated iron complexes are released.

7 $${\left[\left(L\right)M{\left(NH_{3}\right)}_{2}\right]}^{n+}+6R^{\cdot }\to {\left[\left(L\right)M\right]}^{n+}+N_{2}+6RH$$

8 $$2{\left[\left(L\right)M{\left(NH_{3}\right)}_{2}\right]}^{n+}+6R^{\cdot }\to 2{\left[\left(L\right)M\left(NH_{3}\right)\right]}^{n+}+N_{2}+6RH$$

9 $$2{\left[\left(L\right)M{\left(NH_{3}\right)}_{2}\right]}^{n+}+2R^{\cdot }\to 2{\left[\left(L\right)M\left(NH_{3}\right)\right]}^{n+}+{NH}_{2}{NH}_{2}+2RH$$

Our calculated Gibbs free energies (Table 3) demonstrate that dinitrogen production is a thermodynamically feasible process within the catalytic cycle, only when a highly reactive radical partner is employed. While the TEMPO•-mediated pathway is characterized by positive Gibbs free energies (5–35 kcal/mol) rendering the process thermodynamically uphill and thus unfavorable—the employment of the ^t^Bu_3_ArO• radical shifts the reaction to a highly exergonic regime (−55.7 to −85.2 kcal/mol). Our findings are in good accordance with experiments: ^15^N-labeled N_2_ was only detected via NMR when using ^t^Bu_3_ArO• as the oxidant, but not when TEMPO• was used.^27^

**Table 3:** Gibbs Free Energy of N_2_ and N_2_H_4_ Formation at the B3LYP/def2-TZVP Level of Theory^a^

		Intramolecular N_2_ Formation eq 7		Intermolecular N_2_ Formation eq 8		Intermolecular N_2_H_4_ Formation eq 9	
		(kcal mol^–1^)		(kcal mol^–1^)		(kcal mol^–1^)	
Complex	*n*	R^1^	R^2^	R^1^	R^2^	R^1^	R^2^
***09***	+2	n.a.	n.a.	5.7	–85.2	62.9	32.6
	+3	n.a.	n.a.	14.6	–76.3	71.8	41.5
***10***	+2	18.4	–72.5	9.0	–81.9	66.2	35.9
	+3	35.2	–55.7	26.6	–64.3	83.8	53.5
***51***	+2	17.3	–73.6	8.3	–82.6	65.4	35.1
	+3	33.3	–57.7	24.9	–66.0	82.1	51.8

a R^1^ = TEMPO•; R^2^ = ^t^Bu_3_ArO•.

Analysis of the data in Table 3 shows that ammonia oxidation is more favorable (by about 7–16 kcal/mol) when the iron center is in a lower oxidation state. Furthermore, the Gibbs free energy change of the intermolecular pathway is approximately 10 kcal/mol lower (more exergonic) than that of the intramolecular pathway. The formation of hydrazine is characterized by highly positive free energy values (ranging from +35.4 to +83.8 kcal/mol), making it highly unlikely.

### 3.4 HAT Reactions and Isomerization

The thermodynamic facility of ammonia oxidation is closely linked to the ease of hydrogen atom removal from the iron complexes. As established in the literature, a lower Bond Dissociation Free Energy (BDFE) correlates with more favorable oxidation.^49^ Consequently, we calculated the BDFEs and the reaction energies of the hydrogen atom transfer steps to organic radicals (TEMPO• and ^t^Bu_3_ArO•) (see Tables S3–S5 in the SI). While these evaluations may seem straightforward, the reaction landscape is significantly complicated by the frequent spin-state crossovers of the iron center during the HAT process. Figure 4 maps the relative energies of all accessible spin configurations along the sequential HAT pathway for a representative case, complex ***10*** (Fe^2+^) where we set the lowest-energy spin state as the thermodynamic baseline (0.0 kcal mol^–1^) for each intermediate. Comprehensive numerical data for all complexes are summarized in Table S6 in the SI.

**Figure 4:**
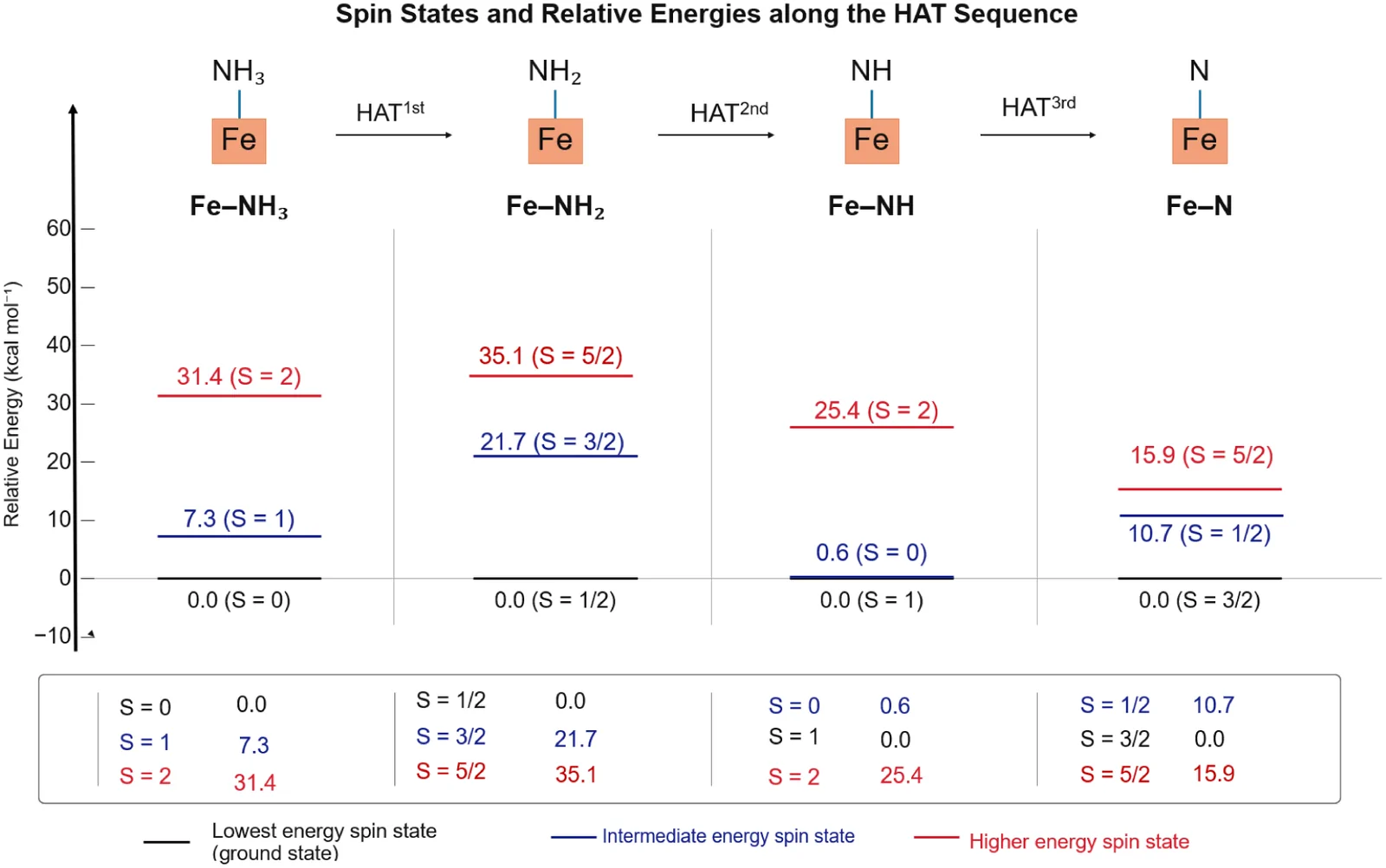
Relative Gibbs free energies of the various spin states of possible intermediates along the 3 HAT steps for complex ***10*** (Fe^2+^) at the PBE/def2-TZVP­(-f) level of theory.

The relative energies of these spin configurations follow a highly consistent pattern. Specifically, the most favorable ground spin states for the distinct intermediates are

- Ammonia (Fe–NH_3_) species: S = 0 for Fe^2+^, and S = 1/2 for Fe^3+^.
- Amido (Fe–NH_2_) species: S = 1/2 for Fe^2+^, and S = 1 for Fe^3+^.
- Imido (Fe=NH) species: S = 1 for Fe^2+^, and S = 1/2 for Fe^3+^.
- Nitrido (Fe≡N) species: S = 3/2 for Fe^2+^, and S = 2 for Fe^3+^.

Beyond thermodynamic evaluations, the proposed spin-state transitions are further corroborated by systematic analysis of the Löwdin charges (q) and spin populations (ρ) at the iron center across successive HAT steps. Our computational results (Table 4) reveal that successive HAT steps induce changes in the electron density around the iron center. Notably, the first two HAT steps trigger only marginal shifts in the Löwdin charge: for complex ***10*** (Fe^2+^), the charge changes modestly from −0.37 to −0.35 between the amido and imido intermediates, while for complex ***10*** (Fe^3+^), a comparably subtle variation from −0.22 to −0.24 is observed over the same steps.

**Table 4:** Charge (*q*) and Spin Population (ρ) on Selected Atoms of ***10*** over HAT at the PBE/def2-TZVP­(-f) Level of Complex ***10***

		Fe–NH_3_		Fe–NH_2_		Fe–NH		Fe–N	
		*q*	ρ	*q*	ρ	*q*	ρ	*q*	ρ
***10*** (Fe^2+^)	Fe	–0.44	0	–0.37	0.66	–0.35	1.04	–0.05	2.37
	N	–0.06	0	–0.13	0.37	–0.09	0.92	–0.002	0.37
***10*** (Fe^3+^)	Fe	–0.27	0.90	–0.22	1.33	–0.24	0.72	0.09	2.87
	N	–0.04	–0.01	–0.07	0.46	0.06	0.21	0.05	0.49

In stark contrast, the third and final HAT^third^ step (Fe–NH → Fe–N) triggers an abrupt and substantial electronic redistribution at the iron center. For complex ***10*** (Fe^2+^), the Löwdin charge shifts markedly from −0.35 to −0.05, accompanied by a dramatic increase in spin population from 1.04 to 2.37. A parallel transformation is observed for complex ***10*** (Fe^3+^), where the charge moves from −0.24 to +0.09 and the spin population surges from 0.72 to 2.87. This simultaneous increase in both formal positive charge and spin density at iron is consistent with the electronic structure analysis of Johnson et al.,^49^ who demonstrated that upon completion of the three-step HAT sequence, the metal center attains formally high-valent nitrido character—most metals reaching a formal M­(V) oxidation state, while the Fe intermediate may adopt a distinct Fe­(III)–N diradical character—ultimately yielding a highly polarized, high-spin iron–nitrido species. Analogous trends observed for complexes ***09*** and ***51*** are summarized in Tables S7–S9 in the SI.

The thermodynamic feasibility of ammonia oxidation is intrinsically dictated by the BDFEs of the N–H bonds within the iron complexes (Table 5). Across all evaluated environments, a distinct trend emerges: Fe^2+^ complexes consistently exhibit BDFE values approximately 7–20 kcal/mol lower than their Fe^3+^ counterparts during the initial HAT step. Specifically, for complex ***10*** in vacuum, the first hydrogen atom transfer (HAT^first^) requires 68.4–73.5 kcal/mol for Fe^2+^, compared to 78.6–90.2 kcal/mol for Fe^3+^ (at PBE and B3LYP levels). However, considering the presence of organic radicals drastically reduces the calculated energies. When TEMPO• or ^t^Bu_3_ArO• is employed, the energetic cost for HAT^first^ in the Fe^2+^ system plummets to 16.7–19.9 kcal/mol and a remarkably low 1.0–4.8 kcal/mol, respectively. This profound decrease, coupled with the highly exothermic nature of the third HAT step in radical environments (e.g., as low as −35.7 kcal/mol with ^t^Bu_3_ArO•), demonstrates that pairing the Fe^2+^ oxidation state with a potent hydrogen-atom acceptor substantially reduces the overall thermodynamic demand, thereby facilitating efficient hydrogen atom abstraction.

**Table 5:** HAT Reaction Free Energy Using the PBE and B3LYP (in Parentheses) Functionals in Conjunction with the def2-TZVP Basis Set^a^,^b^

			HAT^first^	HAT^second^	HAT^third^
Complex	Oxidation State		PBE (B3LYP)	PBE (B3LYP)	PBE (B3LYP)
***10***	Fe^2+^	Spin	1/2	1	3/2
		BDFE	68.4 (73.5)	81.3 (86.9)	53.2 (33.1)
		HAT (R^1^)	16.7 (19.9)	29.6 (33.3)	1.5 (−20.5)
		HAT (R^2^)	1.0 (4.8)	13.9 (18.1)	–14.2 (−35.7)
	Fe^3+^	Spin	1	1/2	2
		BDFE	78.6 (90.2)	83.3 (93.1)	29.7 (15.9)
		HAT (R^1^)	26.9 (36.6)	31.6 (39.5)	–22.0 (−37.7)
		HAT (R^2^)	11.2 (21.4)	15.9 (24.3)	–37.7 (−52.8)

a All energies in kcal mol^–1^.

b R^1^ = TEMPO•; R^2^ = ^t^Bu_3_ArO•.

To further elucidate the electronic origins of these thermodynamic trends, we examined the evolution of N–H BDFE values along the HAT pathway. For complex ***10*** (Fe^2+^), the BDFE rises substantially to 81.3–86.9 kcal/mol at the second HAT step, where the accumulation of positive charge state is in accordance with a formally d^4^ Fe^4+^ imido intermediate in which occupation of degenerate π* orbitals under approximate C_4v_ symmetry destabilizes the linear Fe=N—H geometry, driving a transition to a bent Fe=N—H arrangement and imposing a pronounced thermodynamic bottleneck at this stage of the reaction coordinate.^49^ For the Fe^3+^ precursors, formal charge accumulation is even more pronounced from the outset, and the consistently elevated BDFE values across all three steps reflect the compounding penalty of sustaining highly oxidized intermediates throughout. These “peaked” profiles diverge markedly from the nearly flat BDFE profiles of Group VI Mo and W complexes reported by Johnson et al.,^49^ whose d^2^ configuration sustains a stable linear M≡N—H triple bond at the imido stage without incurring π* destabilization, distributing the energetic cost of HAT more evenly across the reaction coordinate. Beyond these initial barriers, a key finding of this study is the exceptionally low energy of the product of the HAT^third^ reaction in complexes ***09***, ***10***, and ***51***. While the HAT^first^ and HAT^second^ sites generally highly endergonic, the HAT^third^ reaction displays remarkable exergonicity, with reaction energies reaching values as low as −52.8 kcal/mol for Fe^3+^ in complex ***10*** in the presence of ^t^Bu_3_ArO• (at the B3LYP level). Even with the more stable TEMPO• radical, the HAT^third^ step remains highly favorable (e.g., −37.7 kcal/mol for Fe^3+^ in complex ***10***, B3LYP). These highly negative reaction energies are in sharp contrast to previously reported systems in which the HAT^third^ step was only slightly less endergonic than the first two steps.^9,50^

A mechanistic rationale for this striking thermodynamic behavior emerges from the structure of the complexes. Upon the removal of the third hydrogen atom, one might intuitively expect the formation of a classical terminal nitrido species (Fe≡N). Although such nitrido configurations could indeed be located on the potential energy surface in the low-spin state for both Fe^2+^ and Fe^3+^ species, the ground state of the complexes was found to be a structural isomer in the high-spin state in which the nitrido ligand is inserted into the Fe–C bonds of the complexes (see Figure 5).

**Figure 5:**
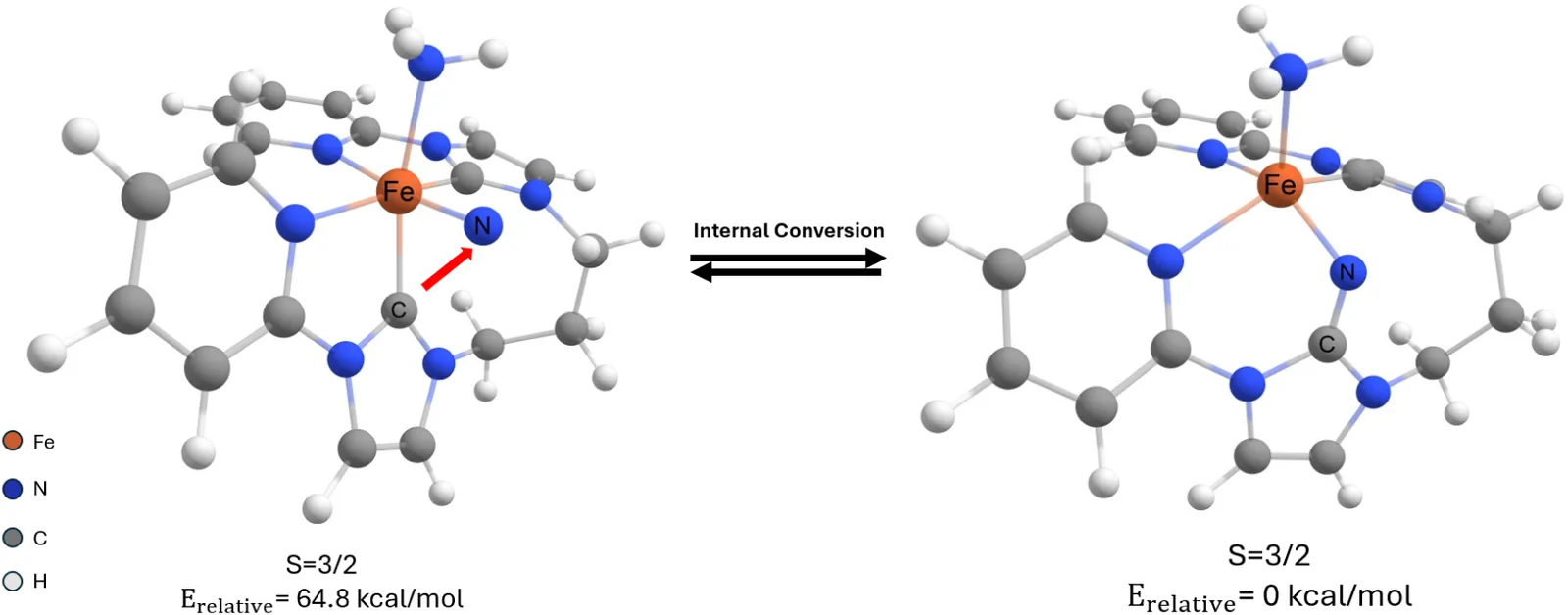
Structural and electronic relaxation of ***10*** (Fe^2+^) complex. The HAT^third^ product was calculated at B3LYP/def2-TZVP­(-f).

The relative energies of the identified species across multiple spin states are detailed in Table S10 in the SI. Across all investigated complexes (***09***, ***10***, and ***51***), the low-spin states typically associated with terminal nitrides are 9 to 50 kcal/mol higher in energy than the ground state with N inserted into the Fe–C bond, regardless of whether PBE or B3LYP functionals are employed. This substantial energetic penalty renders the formation of the reactive terminal nitrido intermediate necessary for N_2_ production thermodynamically prohibitive.

Recent examples of hydrocarbyl^51^ migration from iron­(IV) centers to multiply bound nitrogen ligands include the transfer of alkyl^52,53^ groups to imido ligands, as well as the migration of aryl groups to dinitrogen-derived hydrazido platforms. Given that the previously reported systems feature an iron­(IV) center, we investigated whether our imido species might undergo a similar migration to form new N–C bonds, and indeed, our calculations confirmed that the nitrido species can isomerize to a significantly more stable product featuring an Fe–N–C linkage.

According to our calculations, the two isomers exhibit different ground spin states in both the imido and nitrido species, indicating that the isomerization proceeds via a spin-state crossing (spin-flip) process.

Strictly speaking, the flipping of a spin is a spin-forbidden process under standard quantum-mechanical selection rules. However, such reactions can proceed via nonadiabatic transitions mediated by spin–orbit coupling (SOC). SOC acts as the critical perturbation that mixes the electronic wave functions of the two distinct spin states, enabling the system to cross from one surface to another. The fundamental likelihood of this surface crossing is heavily governed by the magnitude of the SOC and the geometric topography of the crossing seam, as established in foundational theoretical frameworks by Harvey and applied in computational studies of spin-forbidden reactivity.^54,55^

Consequently, we investigated the possibility of the different routes for isomerization: via a transition state in a given spin state and the nonadiabatic spin transition by locating the minimum energy crossing point (MECP) between the relevant interacting spin states.

The ground state of the imido ***10*** (Fe^2+^)–NH complex is triplet, and it lies 43.9 kcal/mol above the quintet ground state of the Fe–N–C isomer. The isomerization barrier on the triplet surface is 75.8 kcal/mol, which is insurmountable under the experimental conditions employed. However, were this reaction to proceed, the resulting triplet Fe–N–C isomer could readily relax to the quintet ground-state product via a low-lying MECP at just 4.39 kcal/mol.

For the nitrido complex ***10*** (Fe^2+^)–N, initial attempts to locate a transition state exclusively on the doublet potential energy surface were unsuccessful. Instead, we found that the isomerization proceeds through a series of spin-state crossings. The ground state of the nitride complex has a quartet configuration, while the doublet (*M* = 2) and sextet (*M* = 6) states lie 13.4 and 6.1 kcal/mol higher in energy, respectively. The doublet nitrido species can be easily converted to a sextet species via ^2,6^MECP lying 14.9 kcal/mol above the doublet, which then easily transforms into the isomerized product in the quartet state via ^4,6^MECP which lies 6.6 kcal/mol above the sextet state (Figure 6). Similar findings were found for the other systems as detailed in Table S11 in the SI.

**Figure 6:**
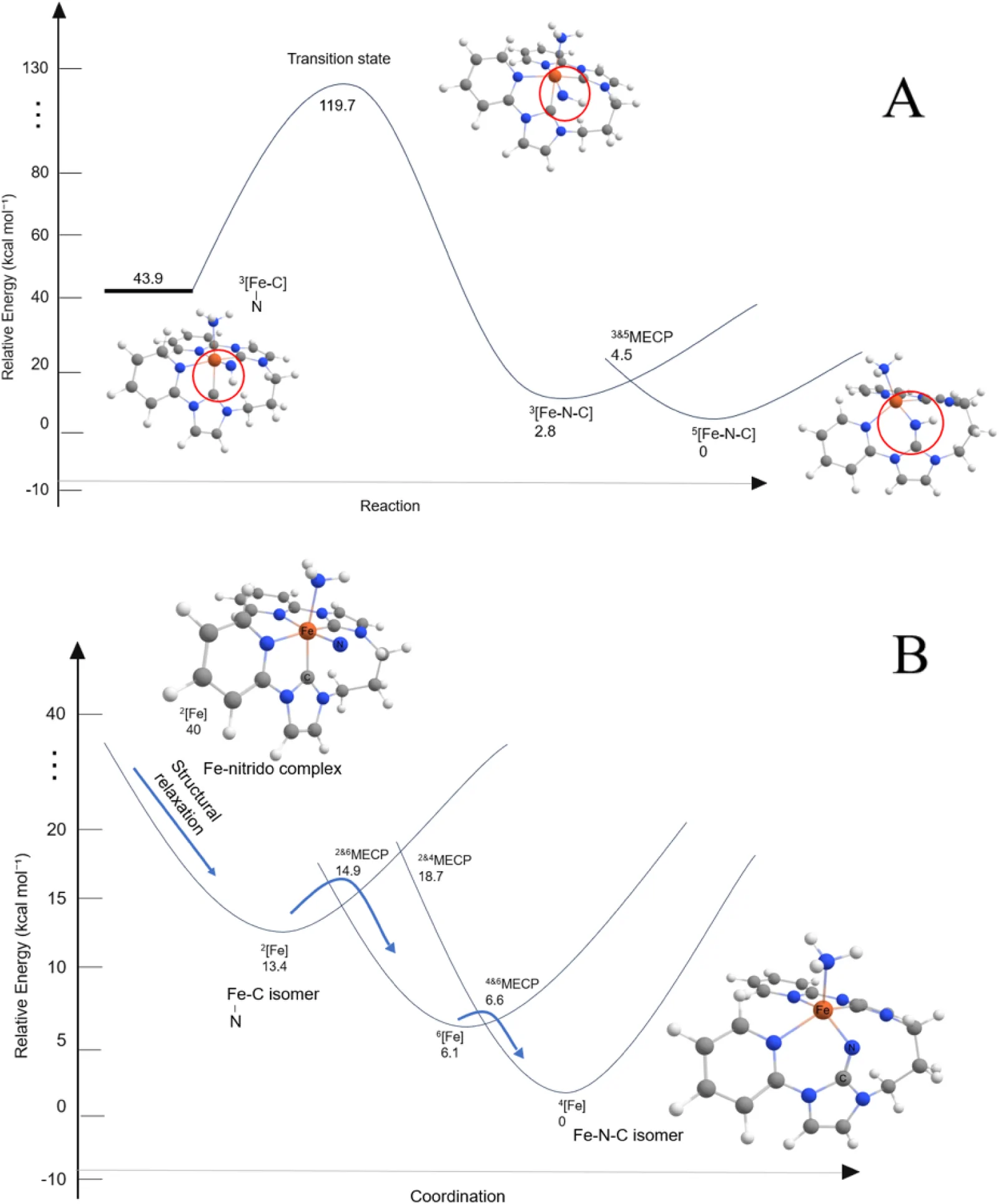
Reaction mechanism for the isomerization of the (A) imido and (B) nitrido complex ***10*** (Fe^2+^) at the B3LYP/def2-TZVP­(-f) level.

Overall, these findings suggest that a substantially more stable Fe–N–C isomer exists on the potential energy surface for both the imido and nitrido species. However, the two systems differ markedly in their propensity to isomerize; in the case of the imido species, isomerization cannot occur under ambient conditions due to the high activation energy. By contrast, the nitrido species is neither kinetically nor thermodynamically stable and readily isomerizes to the Fe–N–C form—similar to the alkyl migration mechanism reported in recent work.^51,52^ These findings provide a plausible explanation for the experimental lack of N_2_ with the exception of the tiny amount of N_2_ that was found with ^t^Bu_3_ArO• radical. Collectively, these results indicate that complexes featuring an Fe–C bond are unlikely to serve as efficient catalysts for ammonia oxidation. The combination of a high-valent iron center and a multiply bonded nitrogen ligand renders the Fe–C bond intrinsically susceptible to alkyl migration, providing a facile deactivation pathway that would compromise catalytic turnover.

## 4 Conclusions

Iron-based molecular catalysts represent a compelling earth-abundant alternative to precious metals for the AO reaction, which sits at the heart of any viable ammonia economy, yet their chronically low turnover numbers and catalyst deactivation. To address this, we applied DFT to map the full sequential HAT energy landscape for a series of Fe^2+^/Fe^3+^ complexes reported by Phearman and Bullock, while simultaneously developing and validating an SMD/DRACO computational protocol employing a van der Waals cavity with Gaussian-smeared surface charge discretization, which achieves Fe^2+^/^3+^ redox potential predictions with a mean absolute error as low as 0.013 V by leveraging the Gaussian-charge vdW surface representation and the DRACO charge correction as the two decisive accuracy-determining factors. Across the three HAT steps, the N–H bond dissociation free energy profile follows a characteristic “peaked” pattern, with a severe thermodynamic bottleneck emerging at the second step due to the destabilization of high-valent Fe–imido intermediates—a liability that is notably absent in Group VI congeners and that fundamentally distinguishes iron’s behavior in this chemistry. More critically, the third HAT step, despite being highly exergonic, does not yield the anticipated terminal nitrido species (Fe≡N). Instead, nitride insertion occurs into the adjacent Fe–C bond, generating an Fe–N–C isomer lying 9–50 kcal/mol below the parent nitrido species. Because this rearrangement is both thermodynamically favorable and kinetically facile, it effectively traps the catalyst in an off-cycle resting state from which further catalytic turnover is precluded. A similar low-energy isomer also exists for the imido intermediate; however, based on our DFT calculations, it is not kinetically accessible under ambient reaction conditions, owing to a prohibitively high isomerization barrier on the relevant spin surface. This asymmetry indicates that the terminal nitrido species, rather than the imido intermediate, represents the primary locus of catalyst deactivation via C–N bond formation, identifying alkyl migration to a multiply bonded iron–nitrogen ligand as a general and previously underappreciated deactivation pathway for high-valent iron catalysts in ammonia oxidation.

## Supplementary Material



## Notes

Energy data for different spin states, calculated redox potentials, bond dissociation energies, reaction energies, charge and spin population changes, MECP relative energies, and optimized geometries with absolute energies for all complexes (PDF)
