# Supplementary material for: Iron–Nitride Isomerization as a Deactivation Pathway in Ammonia Oxidation: A Computational Study of Sequential HAT Mechanisms
Source: Inorg Chem. 2026 Oct 13;65(42):24683–94. doi: 10.1021/acs.inorgchem.6c02749 (PMC13715577; doi:10.1021/acs.inorgchem.6c02749)
Supplement: Supplementary file 1 [file ic6c02749_si_001.pdf]

Supporting information for

Iron–Nitride Isomerization as a Deactivation Pathway in Ammonia Oxidation: A Computational Study of Sequential HAT Mechanisms

*Sheng Luo, Julianna Oláh**

Department of Inorganic and Analytical Chemistry, Faculty of Chemical Technology and Biotechnology, Budapest University of Technology and Economics, Műegyetem rkp. 3., 1111 Budapest, 1111, Hungary

E-mail: *olah.julianna@vbk.bme.hu*

1. All values are in kcal/mol

Table S1. Energy data of difference spin state for LS, IS and HS. Energies in kcal mol^{-1}.

Complex	Ion	State	PBE	TPSSh	B3LYP*	B3LYP	ωB97X	PWPB95	B2PLYP
08	Fe^{2+}	LS	0.0	0.0	0.0	0.0	0.0	0.0	0.0
		IS	23.4	17.6	12.4	8.4	9.1	15.1	19.3
		HS	45.4	29.4	21.6	12.9	9.7	16.4	23.1
	Fe^{3+}	LS	0.0	0.0	0.0	0.0	0.0	0.0	0.0
		IS	13.6	10.1	4.9	1.9	4.3	7.6	8.9
		HS	35.2	23.1	17.3	11.0	12.7	13.5	17.6
09	Fe^{2+}	LS	0.0	0.0	0.0	0.0	0.0	0.0	0.0
		IS	19.8	15.5	10.6	7.4	8.8	13.8	16.6
		HS	40.5	26.0	18.4	10.2	7.5	12.3	17.7
	Fe^{3+}	LS	0.0	0.0	0.0	0.0	0.0	0.0	0.0
		IS	12.9	10.7	5.0	2.2	4.4	6.8	8.6
		HS	32.6	23.9	17.1	10.9	12.7	13.9	17.1
10	Fe^{2+}	LS	0.0	—	0.0	0.0	0.0	0.0	0.0
		IS	11.5	8.8	4.3	1.5	3.2	8.7	9.5
		HS	38.2	23.5	16.1	7.9	5.6	10.5	14.7
	Fe^{3+}	LS	—	0.0	—	0.0	0.0	0.0	0.0
		IS	14.0	10.9	5.2	2.3	4.3	6.9	8.4
		HS	34.6	25.7	19.1	13.2	14.4	15.5	18.4
11	Fe^{2+}	LS	0.0	0.0	0.0	0.0	0.0	0.0	0.0
		IS	9.3	6.7	2.6	−0.2	1.7	6.9	7.7

Complex	Ion	State	PBE	TPSSh	B3LYP*	B3LYP	ωB97X	PWPB95	B2PLYP
		HS	37.2	23.1	16.1	8.1	5.9	10.4	14.8
	Fe^{3+}	LS	0.0	0.0	0.0	0.0	0.0	0.0	0.0
		IS	4.6	4.7	−0.7	−2.8	0.8	3.8	3.5
		HS	31.9	23.0	16.5	10.9	12.7	13.7	16.8
72	Fe^{2+}	LS	0.0	0.0	0.0	0.0	0.0	0.0	0.0
		IS	23.8	17.4	12.2	8.2	8.7	14.3	19.0
		HS	45.0	29.2	21.3	12.5	9.3	15.2	21.6
	Fe^{3+}	LS	0.0	0.0	0.0	0.0	0.0	0.0	0.0
		IS	16.2	11.8	5.9	2.5	4.0	6.5	9.1
		HS	37.6	27.4	20.3	13.8	14.7	16.0	19.8
51	Fe^{2+}	LS	0.0	0.0	0.0	0.0	0.0	0.0	0.0
		IS	11.2	8.6	4.2	1.4	3.1	8.5	9.4
		HS	38.0	23.4	16.1	8.0	5.7	10.4	14.8
	Fe^{3+}	LS	0.0	0.0	0.0	0.0	0.0	0.0	0.0
		IS	14.0	10.8	5.2	2.3	4.3	6.8	8.6
		HS	34.8	25.9	19.3	13.4	14.6	15.8	18.7
28	Fe^{2+}	LS	0.0	0.0	0.0	0.0	0.0	0.0	0.0
		IS	21.4	18.6	12.1	15.1	16.6	18.5	20.7
		HS	49.2	40.3	30.2	21.6	18.7	32.8	35.8
	Fe^{3+}	LS	0.0	0.0	0.0	0.0	0.0	0.0	0.0
		IS	17.7	16.8	10.3	7.1	9.0	15.9	16.2
		HS	51.2	48.5	39.7	33.9	37.2	44.7	44.3

Table S2. Calculated Redox Potentials (V) for Six Benchmark Complexes (08, 09, 10, 72, 51, 28), Evaluated Across All Combinations of Solvation Cavity Surface Type, Charge Discretization Scheme, and DRACO Correction, Using Seven Density Functionals (def2-TZVP basis set). The bottom row of each section reports the mean absolute error (MAE) relative to the experimental value.

Complex	Exp (V)	PBE	B3LYP	B3LYP*	TPSSh	ωB97X	B2PLYP	PWPB95
**GEPOL-SAS + DRACO**								
08	0.27	0.614	1.048	0.970	0.947	0.991	0.939	0.902
09	0.05	0.552	0.973	0.882	0.795	0.948	0.814	0.822
10	0.10	1.170	1.476	1.408	1.332	1.412	1.273	1.308
72	0.51	1.157	1.450	1.392	1.341	1.362	1.326	1.305
51	0.075	1.009	1.344	1.270	1.194	1.296	1.145	1.180
28	0.02	0.765	0.960	0.924	0.908	0.835	0.892	0.850
MAE		**0.707**	**1.038**	**0.970**	**0.915**	**0.970**	**0.894**	**0.890**
**GEPOL-SES, no DRACO**								
08	0.27	0.077	0.511	0.433	0.411	0.455	0.402	0.365
09	0.05	-0.148	0.273	0.183	0.096	0.248	0.114	0.122
10	0.10	0.081	0.386	0.318	0.242	0.322	0.184	0.219
72	0.51	0.472	0.766	0.707	0.656	0.678	0.641	0.621
51	0.075	0.033	0.369	0.295	0.218	0.320	0.170	0.205
28	0.02	0.039	0.234	0.198	0.182	0.109	0.167	0.124

Complex	Exp (V)	PBE	B3LYP	B3LYP*	TPSSh	ωB97X	B2PLYP	PWPB95
MAE		0.085	0.252	0.185	0.130	0.184	0.109	0.105
Gaussian (vdW), no DRACO								
08	0.27	0.025	0.459	0.381	0.359	0.403	0.351	0.313
09	0.05	-0.233	0.188	0.098	0.010	0.163	0.029	0.037
10	0.10	-0.018	0.287	0.219	0.143	0.223	0.085	0.120
72	0.51	0.407	0.700	0.642	0.591	0.613	0.576	0.556
51	0.075	-0.098	0.238	0.164	0.088	0.189	0.039	0.074
28	0.02	-0.019	0.177	0.141	0.125	0.052	0.109	0.067
MAE		0.160	0.171	0.103	0.062	0.103	0.051	0.028
GEPOL-SES + DRACO								
08	0.27	0.021	0.455	0.378	0.355	0.399	0.347	0.310
09	0.05	-0.175	0.246	0.156	0.069	0.221	0.088	0.095
10	0.10	-0.023	0.282	0.214	0.138	0.218	0.080	0.115
72	0.51	0.400	0.693	0.635	0.583	0.605	0.569	0.548
51	0.075	-0.057	0.278	0.204	0.128	0.230	0.079	0.114
28	0.02	-0.049	0.146	0.110	0.094	0.021	0.079	0.037
MAE		0.151	0.179	0.112	0.057	0.111	0.043	0.032
Gaussian (vdW) + DRACO								
08	0.27	0.014	0.448	0.370	0.347	0.391	0.339	0.302
09	0.05	-0.217	0.204	0.113	0.026	0.179	0.045	0.053
10	0.10	-0.028	0.278	0.210	0.134	0.214	0.076	0.111
72	0.51	0.375	0.668	0.610	0.558	0.580	0.544	0.523

Complex	Exp (V)	PBE	B3LYP	B3LYP*	TPSSh	ωB97X	B2PLYP	PWPB95
51	0.075	-0.081	0.255	0.181	0.104	0.206	0.056	0.091
28	0.02	-0.060	0.136	0.100	0.084	0.011	0.068	0.026
MAE		0.170	0.161	0.093	0.046	0.096	0.033	0.013

Table S3. BDEs (kcal/mol) under Vacuum at PBE and B3LYP/ def2-TZVP level. Energies in kcal mol^{-1}.

Compound	Oxidation State	HAT^{1st} E (kcal mol^{-1}), PBE (B3LYP)	HAT^{2nd} E (kcal mol^{-1}), PBE (B3LYP)	HAT^{3rd} E (kcal mol^{-1}), PBE (B3LYP)
51	Fe^{2+}	67.7 (73.2)	83.5 (88.8)	35.2 (26.2)
10		68.4 (73.5)	81.3 (86.9)	37.2 (29.2)
09		68.5 (74.2)	84.7 (89.3)	33.6 (24.8)
51	Fe^{3+}	82.0 (92.7)	87.3 (95.6)	21.7 (10.2)
10		78.6 (90.2)	83.3 (93.1)	29.7 (15.9)
09		76.0 (89.1)	83.6 (90.9)	31.7 (17.9)

Values in parentheses calculated at the B3LYP level; unparenthesized values at the PBE level.

HAT = hydrogen atom transfer. Energies in kcal mol^{-1}.

Table S4. Reactions energy (kcal/mol) with TEMPO• at PBE and B3LYP/ def2-TZVP level

Compound	Oxidation State	HAT^{1st} E (kcal mol^{-1}), PBE (B3LYP)	HAT^{2nd} E (kcal mol^{-1}), PBE (B3LYP)	HAT^{3rd} E (kcal mol^{-1}), PBE (B3LYP)
51	Fe^{2+}	16.0 (19.6)	31.8 (35.2)	-16.5 (-27.4)
10		16.7 (19.9)	29.6 (33.3)	-14.3 (-24.4)
09		16.8 (20.5)	33.0 (35.7)	-18.1 (-28.8)
51		30.3 (39.1)	35.6 (42.0)	-30.0 (-43.4)

10	Fe^{3+}	26.9 (36.6)	31.6 (39.5)	-22.0 (-37.7)
09		24.3 (35.5)	31.9 (37.3)	-20.0 (-35.7)

Values in parentheses calculated at the B3LYP level; unparenthesized values at the PBE level.

HAT = hydrogen atom transfer. Energies in kcal mol^{-1}.

Table S5.Reactions energy (kcal/mol) with $^{t}Bu_3ArO$• at PBE and B3LYP / def2-TZVP level of theory

Compound	Oxidation State	**HAT^{1st}** *E* (kcal mol^{-1}), PBE (B3LYP)	**HAT^{2nd}** *E* (kcal mol^{-1}), PBE (B3LYP)	**HAT^{3rd}** *E* (kcal mol^{-1}), PBE (B3LYP)
51	Fe^{2+}	0.3 (4.4)	16.1 (20.1)	-32.2 (-42.6)
10		1.0 (4.8)	13.9 (18.1)	-30.0 (-39.5)
09		1.1 (5.4)	17.3 (20.5)	-33.8 (-44.0)
51	Fe^{3+}	14.6 (23.9)	19.9 (26.9)	-45.7 (-58.5)
10		11.2 (21.4)	15.9 (24.3)	-37.7 (-52.8)
09		8.6 (20.3)	16.2 (22.1)	-35.7 (-50.9)

Values in parentheses calculated at the B3LYP level; unparenthesized values at the PBE level.

HAT = hydrogen atom transfer. Energies in kcal mol^{-1}.

Table S6. Calculated relative energies (in kcal mol^{-1}) of various spin states for the sequential HAT pathway intermediates across all complexes.

Complex	M	Fe–NH_3	M	Fe–NH_2	M	Fe–NH	M	Fe–N
09	1	0.0	2	0.0	1	0.2	2	26.6
	3	15.8	4	24.7	3	0.0	4	0.0
	5	33.2	6	37.4	5	22.3	6	18.0
	2	0.0	1	5.3	2	0.0	1	17.8
	4	10.2	3	0.0	4	7.8	3	6.2
	6	27.9	5	13.5	6	31.5	5	0.0
10	1	0.0	2	0.0	1	0.6	2	10.7
	3	7.3	4	21.7	3	0.0	4	0.0
	5	31.4	6	35.1	5	25.4	6	15.9
	2	0.0	1	5.9	2	0.0	1	23.5
	4	10.9	3	0.0	4	7.6	3	45.7
	6	29.4	5	16.8	6	34.5	5	0.0
51	1	0.0	2	0.0	1	2.4	2	10.7
	3	7.0	4	21.7	3	0.0	4	0.0
	5	31.2	6	35.2	5	23.7	6	16.0
	2	0.0	1	3.7	2	0.0	1	23.2
	4	10.8	3	0.0	4	6.3	3	44.4
	6	29.5	5	12.6	6	21.8	5	0.0

Table S 7. Charge (q) and Spin Pop (ρ) change over HAT at the PBE(B3LYP)/def2-TZVP(-f) level of Complex 09

		Fe–NH_3		Fe–NH_2		Fe–NH		Fe–N	
		q	*ρ*	*q*	*ρ*	*q*	*ρ*	*q*	*ρ*
**09(Fe^{2+})**	Fe	−0.45 (−0.41)	0 (0)	−0.36 (−0.30)	0.68 (0.75)	−0.36 (−0.32)	1.00 (0.93)	−0.06 (-0.05)	2.36 (2.59)
	N	−0.06 (−0.08)	0 (0)	−0.14 (−0.18)	0.34 (0.29)	−0.09 (−0.08)	0.96 (1.07)	-0.001 (−0.05)	0.36 (0.27)
**09(Fe^{3+})**	Fe	−0.33 (−0.18)	0.68 (0.99)	−0.27 (−0.16)	1.12 (1.39)	−0.27 (−0.15)	0.64 (1.60)	0.05 (0.16)	2.76 (2.99)
	N	−0.04 (−0.05)	−0.01 (−0.01)	−0.09 (−0.07)	0.41 (0.49)	0.01 (0.01)	0.09 (−0.61)	0.04 (0.04)	0.45 (0.52)

Table S 8. Charge (q) and Spin Pop (ρ) change over HAT at the PBE(B3LYP)/def2-TZVP(-f) level of Complex 10

		Fe–NH_3		Fe–NH_2		Fe–NH		Fe–N	
		q	*ρ*	*q*	*ρ*	*q*	*ρ*	*q*	*ρ*
**10(Fe^{2+})**	Fe	−0.44 (−0.40)	0 (0)	−0.37 (−0.30)	0.66 (0.73)	−0.35 (−0.30)	1.04 (0.98)	-0.05 (0.05)	2.37 (2.58)
	N	−0.06 (−0.08)	0 (0)	−0.13 (−0.18)	0.37 (0.31)	−0.09 (−0.09)	0.92 (1.03)	−0.002 (−0.05)	0.37 (0.29)
**10(Fe^{3+})**	Fe	−0.27 (−0.16)	0.90 (1.04)	−0.22 (−0.13)	1.33 (1.45)	−0.24 (−0.14)	0.72 (1.61)	0.09 (0.18)	2.87 (3.06)
	N	−0.04 (−0.05)	−0.01 (−0.01)	−0.07 (−0.04)	0.46 (0.56)	0.06 (0.04)	0.21 (−0.50)	0.05 (0.04)	0.49 (0.49)

Table S9. Charge (q) and Spin Pop (ρ) change over HAT at the PBE(B3LYP)/def2-TZVP(-f) level of Complex 51

		Fe–NH_3		Fe–NH_2		Fe–NH		Fe–N	
		q	*ρ*	*q*	*ρ*	*q*	*ρ*	*q*	*ρ*
**51(Fe^{2+})**	Fe	−0.44 (−0.40)	0 (0)	−0.36 (−0.30)	0.66 (0.73)	−0.36 (−0.32)	1.01 (0.94)	-0.05 (0.05)	2.38 (2.58)
	N	−0.06 (−0.08)	0 (0)	−0.14 (−0.18)	0.36 (0.31)	−0.09 (−0.09)	0.95 (1.06)	−0.005 (−0.05)	0.37 (0.29)
**51(Fe^{3+})**	Fe	−0.27 (−0.16)	0.88 (1.04)	−0.24 (−0.15)	1.34 (1.46)	−0.27 (−0.14)	0.54 (1.62)	0.09 (0.18)	2.86 (3.06)
	N	−0.07 (−0.08)	−0.01 (−0.01)	−0.10 (−0.06)	0.46 (0.55)	0.04 (0.03)	0.43 (−0.53)	0.05 (0.04)	0.48 (0.49)

Table S10.Relative spin-state energies (kcal/mol) and isomerization accessibility for complexes 09, 10, and 51 at PBE and B3LYP/def2-TZVP(-f) levels of theory

		PBE		B3LYP	
**Compound**	**Spin**	**E (kcal mol⁻¹)**	**Isomerization**	**E (kcal mol⁻¹)**	**Isomerization**
**09(Fe^{2+})**	2	26.6		45.9	
	4	0.0	✓	0.0	✓
	6	18.0	✓	6.9	✓
**09(Fe^{3+})**	1	17.8	✓	28.6	✓
	3	6.2	✓	4.1	✓
	5	0.0	✓	0.0	✓
**10(Fe^{2+})**	2	10.7	✓	13.9	✓
	4	0.0	✓	0.0	✓
	6	15.9	✓	3.9	✓
**10(Fe^{3+})**	1	23.5		50.0	
	3	45.7		69.3	
	5	0.0	✓	0.0	✓

**51(Fe^{2+})**	2	10.7	✓	14.7	✓
	4	0.0	✓	0.0	✓
	6	16.0	✓	4.9	✓
**51(Fe^{3+})**	1	23.2		49.9	
	3	44.4		3.1	✓
	5	0.0	✓	0.0	✓

Energies relative to the lowest-energy spin state (0.0 kcal/mol) for each compound/oxidation state.

✓ indicates spin states accessible via thermal isomerization; blank entries are not accessible.

Table S11. MECP relative energy(kcal mol^{-1}) for complex ***10*** and ***51*** with def2-TZVP basis set.

		^{2}[Fe]	^{4}[Fe]	^{6}[Fe]	$^{2\&4}$MECP	$^{2\&6}$MECP	$^{4\&6}$MECP
B3LYP	***10***(Fe^{3+})	13.40	0.00	6.08	18.66	14.90	6.55
	51(Fe^{3+})	21.01	45.40	0.00	53.31	10.36	4.83
PBE	***10***(Fe^{3+})	13.42	0.00	6.37	19.08	14.97	6.81
	51(Fe^{3+})	46.64	3.07	0.00	18.26	17.73	3.37
		^{1}[Fe]	^{3}[Fe]	^{5}[Fe]	$^{1\&3}$MECP	$^{1\&5}$MECP	$^{3\&5}$MECP
B3LYP	***10***(Fe^{2+})	9.37	0.00	17.60	10.64	19.41	18.22
	51(Fe^{2+})	21.01	45.40	0.00	53.31	10.36	4.83

PBE	*10*(Fe^{2+})	9.42	0.00	17.83	10.77	19.53	18.50
	51(Fe^{2+})	20.77	44.31	0.00	52.35	10.63	4.67

2. Optimized geometry, its energy and Spin.

CCSD identifier	E	charge	Spin
2271708	-2858.680173724753	+2	0

Fe	1.91919993582706	9.91270515786011	2.98262489884175
N	2.61553872300827	11.35918978777684	4.07595267246428
N	3.04468142257730	8.74897347860114	3.94515827529252
N	0.84334412695388	11.32879834925678	2.06282874417342
N	-0.79783943613132	14.73715832198482	0.23561271122579
N	2.77069540125226	11.47640730681124	0.85632284874521
N	4.15766562170018	9.81598293155637	0.91315572366285
N	1.36760184357721	7.62979706549719	0.99673952400841
N	0.17635415477604	7.75161623785915	2.79417178075407
N	0.43124890446203	9.44978479824223	4.29780916000181
N	-2.62319691757721	7.97038313135709	6.77517381749897
C	3.07185818069640	12.22967177558777	4.69617870818750
C	3.64785636727935	13.31535645354955	5.46434991491591
H	3.02147777768468	14.21443408728584	5.37842048531124
H	4.65563842640621	13.54979482550373	5.09282023310357
H	3.71996027539174	13.03561330172030	6.52492117893416
C	3.67572646306862	8.06714914455852	4.64635743011520
C	4.47685848074834	7.22283443339284	5.51047604454112
H	4.14258683695694	6.17794866599963	5.43986031367813
H	4.38165160480127	7.55039768346701	6.55563623740653
H	5.53695362119475	7.27426199632154	5.22293714043414
C	-0.40205285806414	11.76553231041714	2.36080121007837
H	-0.94522077474780	11.18032188618408	3.10047961876537
C	-0.98433933720058	12.87183652173149	1.78192316889558
H	-1.99773239347923	13.13625445749664	2.07193245850662
C	-0.26680621819779	13.64023992361981	0.82112506831783
C	-2.15288807861296	15.16778343150042	0.57174608849047
H	-2.89773459140833	14.40669692489178	0.29183935005689
H	-2.38075475111229	16.08653772012710	0.02431735824121
H	-2.24694768369980	15.38015466151503	1.64737890676695
C	-0.03812385248738	15.47724733361053	-0.76662602358155
H	0.89695862793641	15.87853494044807	-0.34518761968155
H	-0.63833520258850	16.32033941539704	-1.11930604578587
H	0.20376627982343	14.84381936390904	-1.63478427107273
C	1.04194876251485	13.18334471985449	0.50906534067823
H	1.66503612313140	13.70151856997719	-0.21459154971208
C	1.52238001800028	12.05236825678888	1.13698809156280
C	3.07835119950742	10.33091875011152	1.56162550530543
C	3.64583756254642	11.66637727092970	-0.21048428551817
H	3.59798000088467	12.51470172674498	-0.88161605629635
C	4.51083633529308	10.61987680883514	-0.17409776525621
H	5.35416059465016	10.38774587848712	-0.81459762137100
C	4.51587931139858	8.40445129839016	1.03612095914201
H	4.28982758076391	8.11097726238381	2.06422972438358
H	5.59544890362858	8.28872747043833	0.87358505687683

C	3.72667772448621	7.54920101645148	0.02858970302760
H	4.18143262192871	7.64858413404830	-0.96918535659500
H	3.82684830987557	6.49009547643920	0.30977944716736
C	2.25059541872728	7.92209150650355	-0.13954227565005
H	2.14269296635226	8.98714996186914	-0.38128893011260
H	1.84488027727597	7.36580702360311	-0.99405262727384
C	1.16719248302318	8.40895671791399	2.09559874502188
C	0.52551640033983	6.51705181367628	1.01997351832171
H	0.52400847526820	5.78146660914213	0.22407878104024
C	-0.22489217616042	6.58949509512024	2.14809217922854
H	-0.99570763145551	5.92802435751879	2.52182836288499
C	-0.25739526481223	8.32220487021881	4.00211030344211
C	-1.27005073560661	7.78590307741252	4.77279547667707
H	-1.77070202847272	6.87725122995686	4.45125267915822
C	-3.34217925715683	6.75224657429143	6.41584151279412
H	-3.87128778842400	6.86696024657053	5.45654023777528
H	-4.08451347566024	6.53517115141403	7.18861758893944
H	-2.65974894744826	5.89055459080244	6.34716969698720
C	-2.97913850239715	8.67816241519973	8.00297847927303
H	-2.12599034220565	8.72925371801654	8.69657412147937
H	-3.79066876110256	8.14051985614751	8.50109841616648
H	-3.32699991196439	9.70032615009142	7.78872902506204
C	-1.64011395057538	8.44372198534940	5.97706870270811
C	-0.91548990967187	9.62955836291485	6.28988857164707
H	-1.12879301358931	10.19995124172975	7.19013359869232
C	0.07992525438239	10.07247936914690	5.44649762351654
H	0.63828355690917	10.97802138347144	5.68073163253140

CCSD identifier	E	charge	Spin
2271708	-2858.275319113925	+3	1/2

Fe	1.85920203668090	9.94419537293750	2.96692868660944
N	2.61516675854505	11.39510667269257	4.08634439800507
N	3.00399783718928	8.75950661501470	3.97078567331211
N	0.83449639640671	11.35604271508109	2.06454063375512
N	-0.80907895417242	14.75685360779726	0.23698214039226
N	2.73732402664093	11.46759507614818	0.81321581985991
N	4.12030359061209	9.80839468410766	0.89891144933886
N	1.37012510421063	7.61402118629836	0.98187190375527
N	0.17306395151933	7.74047437482249	2.77911990023666
N	0.38082559629189	9.46992643750324	4.26875268877547
N	-2.62371191661326	7.93807093980902	6.76364028193272
C	3.12953491676425	12.23071418107877	4.70639128181161
C	3.77300630947152	13.26899284339622	5.48112623329162
H	3.16074341004432	14.18235032533117	5.46999784019852
H	4.76075131507902	13.49808254733381	5.05489105521727
H	3.90229180078547	12.93991565703278	6.52238826727032
C	3.64623306825329	8.10188305414192	4.68022038516934
C	4.46020232352534	7.28448632093288	5.55235742110291
H	4.27644047478267	6.21884376161902	5.35115439938931
H	4.21772095004899	7.49448159314872	6.60423912929361
H	5.52603255035046	7.49838304177384	5.38344296674474
C	-0.39434192315125	11.82536708619440	2.40601744532156
H	-0.92660590702108	11.26054459676896	3.16747464911605
C	-0.97160987341222	12.93035259107964	1.82778935398048
H	-1.97382708803915	13.21245765447743	2.14020344578547
C	-0.27332658689316	13.67065978062624	0.82548666672690
C	-2.13517839570701	15.24366435052303	0.63108005800595
H	-2.91658200163213	14.51657503888346	0.36318818444953
H	-2.34048292035334	16.18010574608053	0.10650237282372
H	-2.17417232353172	15.43962044840246	1.71170279526013
C	-0.08573575583946	15.46348102311653	-0.82255117150318
H	0.84024207056951	15.91487620414732	-0.43435966824589
H	-0.71862398687919	16.26324768512292	-1.21467009596130
H	0.15872268958084	14.78157922479977	-1.65022896072729
C	1.02214532760175	13.18880324504522	0.48024747732135
H	1.63389482463088	13.69334474472268	-0.26269591271236
C	1.50271150837060	12.06153611762987	1.10449094114405
C	3.03779918629636	10.32930528150303	1.52122293017119
C	3.63289725922413	11.66085768557578	-0.23363940430492

H	3.59815322494465	12.50617625751775	-0.91015090899365
C	4.49906303110591	10.61373814643871	-0.17627593606697
H	5.35530174819655	10.38039375087570	-0.80023223239550
C	4.50561399216604	8.40182575158153	1.04145335332339
H	4.28053427633599	8.11262881394698	2.07082273253254
H	5.58795091592316	8.30818879380395	0.88761428687678
C	3.73779496793070	7.53041592317340	0.03215960851054
H	4.20265829555665	7.62304305758783	-0.96124719824943
H	3.84780733942175	6.47522431599092	0.32292895724336
C	2.26185777283759	7.88715404785441	-0.16065133145979
H	2.13825707182069	8.94284422052840	-0.43259919021989
H	1.86569460275631	7.30234976800046	-0.99985755956026
C	1.14544050266667	8.40018462332625	2.06497420304268
C	0.55538610669436	6.48735041889228	1.02509815107639
H	0.56804351062906	5.73743554991079	0.24167926800984
C	-0.20110312436905	6.56348963328719	2.15328472497783
H	-0.96231336636678	5.89181986937601	2.53037507462547
C	-0.27601346229463	8.31879010812435	3.98557496305171
C	-1.27083327695709	7.76584527960681	4.76109471229006
H	-1.75077608151727	6.84126548486947	4.45287359125178
C	-3.32094169597647	6.69626402875671	6.42132318281327
H	-3.85231085367416	6.79428742172923	5.46264460347910
H	-4.05694860089073	6.47568817868111	7.19819668433075
H	-2.61729797566210	5.85220348059110	6.36583772236643
C	-3.00044630386721	8.64565495773349	7.99112091348097
H	-2.14069231323623	8.73449758137038	8.67131871647314
H	-3.78547796930437	8.08175311355083	8.50086436963002
H	-3.38730308344314	9.64969413815879	7.76224885432094
C	-1.65772414945211	8.42937473407506	5.96361017345593
C	-0.96406199398957	9.64014678076343	6.26313254862296
H	-1.18993302237865	10.21330417965952	7.15867318491809
C	0.01435724937480	10.10108518115224	5.41406187328986
H	0.54926201478780	11.02308689638345	5.63748224083774

CCSD identifier	E	charge	Spin
2271709	-2782.545567799280	+2	0

Fe	4.37971970846872	10.01032054571112	1.96713943217261
N	5.15070702555796	9.17956794256222	0.40171700061048
N	5.73266432386119	8.85775080716784	3.01180043868452
H	5.52940019534114	7.87601378345965	2.79583939400483
H	5.70378489483729	8.93767879554575	4.03203043942205
H	6.69915203873300	9.02547454677797	2.71861368215226
N	3.21733962883908	11.00832302952747	0.69845071459996
N	1.39455662632494	13.09355141609832	-2.46222558181195
N	5.04396826395831	12.37060947222155	0.68839152977741
N	6.43394928264522	12.24644362870766	2.34475165155380
N	3.69001099001798	11.61695380703106	4.49398809153450
N	2.70835385368596	9.71827727107374	4.17141563146755
N	3.04720333879706	8.49558260112200	2.27495366723892
N	0.27089511227397	5.56272770994995	3.45240233558319
C	5.61933183039501	8.72962383485725	-0.56489810381493
C	6.20524014165748	8.18113264164927	-1.77169645260343
H	5.50352165945827	8.28226985345803	-2.61197577306965
H	6.43791826250917	7.11542898429426	-1.63464161023546
H	7.13383020201780	8.71433474827276	-2.02155796858656
C	1.98989084236928	10.67218810004069	0.23834216017423
H	1.51160994843601	9.82944457937566	0.73433246966245
C	1.34972841392435	11.33240734907996	-0.78792017965480
H	0.35674730285352	10.99988588017255	-1.07869197040941
C	1.98348812064328	12.42650685526459	-1.44371603500990
C	0.05685909601105	12.71653196998196	-2.91214578988751
H	0.03335433839268	11.67338871902304	-3.26256535412328
H	-0.23332172131998	13.36077734885205	-3.74665434642008
H	-0.68622339124893	12.83968602214249	-2.10909228589411
C	2.07421791986036	14.21880122367417	-3.09518364560976

H	2.25075002200321	15.03714296817658	-2.37890092016120
H	1.44850366146109	14.60526494204201	-3.90423604277229
H	3.03805365925444	13.90982177615086	-3.52901432183987
C	3.27230645210328	12.78011333418342	-0.96142242344929
H	3.83073018772563	13.60668862000161	-1.39191618239578
C	3.81466879671679	12.06219224683496	0.08532255318231
C	5.41339557023667	11.56242241090711	1.74773407977698
C	5.81845944633004	13.52690843285388	0.63270463256996
H	5.70968718357176	14.28593622418478	-0.13189791173771
C	6.68474834992836	13.44811543902459	1.67485210750757
H	7.46876581533338	14.13017762330903	1.98421623364135
C	6.79445438001596	12.01970123471899	3.74239678689037
H	7.85351093111207	12.26773896935367	3.89151791507446
H	6.67154358243076	10.95156152603161	3.94126516739220
C	5.90817862722178	12.85264898301316	4.68422351903513
H	6.05171153907351	12.49463289774373	5.71487633652891
H	6.24853945145262	13.89931109946800	4.66926273485861
C	4.41972328491410	12.87899963873119	4.32531670736094
H	3.91738626046239	13.61579798258070	4.96491287509610
H	4.27348190163815	13.21186927070069	3.28995727684368
C	2.88268666770380	11.36580765882231	5.60399636609624
H	2.80982311331995	12.06185555857444	6.43165006735145
C	2.26336617080864	10.17527253363374	5.40603707824196
H	1.55708650020306	9.64000593975569	6.02739822870847
C	3.59920908673146	10.60096153933110	3.58448104746155
C	2.36924169527792	8.56405978693585	3.44891618936879
C	1.44841910971097	7.63504731990065	3.89074506294919
H	0.95056613161266	7.77807109429894	4.84559845125175
C	-0.42448030290645	5.67493633159350	4.73114560494430
H	0.28684464184973	5.69369938032522	5.57154883496561
H	-1.07701282648981	4.80733651874442	4.86057074609678
H	-1.04955807406289	6.58112497059000	4.77041777244653
C	-0.00766977545342	4.42172347102850	2.58232271322301
H	-0.41326421098158	4.74794082035127	1.61242805225051
H	-0.75166137354310	3.78151584488268	3.06392016962637
H	0.89861590505773	3.82200924928577	2.40771746435277
C	1.16022915307447	6.50703227252164	3.07504073718140
C	1.86246410524537	6.43640683539844	1.83656311929980
H	1.70178220410169	5.61394501068022	1.14450022588587
C	2.76467799576161	7.42085766966154	1.49760208824330
H	3.30003673069094	7.37286110657809	0.54972831514394

CCSD identifier	E	charge	Spin
2271709	-2782.140788471873	+3	1/2

Fe	4.32104425697081	10.01857111896514	1.92935072527091
N	5.15528029320034	9.15587344787206	0.34993914616605
N	5.67446929236638	8.86076662571064	2.96477907500736
H	5.47576941051979	7.87641965055490	2.75466064433338
H	5.65321692207670	8.94839307457561	3.98499907493940
H	6.63948722673722	9.03340836620527	2.66606138247064
N	3.20875601790976	11.00603658101852	0.65873984525337
N	1.37539521703827	13.11294006753896	-2.47223865063508
N	5.00205662342412	12.41525003745707	0.70436324112044
N	6.40161085790155	12.25025513932808	2.34388805270463
N	3.69509026689146	11.62697894215386	4.50458622379425
N	2.69115655305295	9.74011860154253	4.16496875443339
N	2.99055157777767	8.51776700621338	2.24712643012091
N	0.26624880474526	5.57039667253008	3.48318494544819
C	5.68895164660820	8.69554264937688	-0.57424209707444
C	6.35331022856059	8.12362627709016	-1.72433168931921
H	5.69625776583092	8.18219208956434	-2.60449838588415
H	6.60017302879255	7.06860496997713	-1.53492907510583
H	7.28193950061310	8.67400648357940	-1.93631349833192

C	2.00405539422353	10.62744371785640	0.15324610480951
H	1.54448135107291	9.75652230111006	0.61474034577392
C	1.36601414614139	11.29419400829198	-0.86435600346466
H	0.39252496095650	10.93392456666410	-1.18733600163961
C	1.97145673078664	12.43648263725609	-1.47297950689052
C	0.05137550460228	12.71259369836633	-2.96109820718802
H	0.08658333815656	11.71185620085376	-3.41651394056938
H	-0.27688255456548	13.42581361769545	-3.72100945205746
H	-0.68408004289640	12.71652493410174	-2.14400481968345
C	2.03073163679032	14.27189177745431	-3.08364551473245
H	2.14096455399411	15.09025442062516	-2.35573458122084
H	1.41860985267137	14.63101505097119	-3.91441176728809
H	3.01962562137751	13.99806668723877	-3.47949773128179
C	3.24370503044944	12.82014414177491	-0.95752422681682
H	3.78222806751371	13.67415171302773	-1.35909611246021
C	3.78806669138868	12.09948755988961	0.07917936790783
C	5.37387412899123	11.59734689928577	1.74533860838220
C	5.79609566731578	13.55691008170614	0.64772781076626
H	5.69333260980992	14.32789935717818	-0.10630634811084
C	6.67218648765551	13.44972773234748	1.68083320020664
H	7.47106162831786	14.11507652921646	1.99119003820703
C	6.80293428187786	11.99579285580312	3.73051717054741
H	7.86526302009616	12.24303244568292	3.85052253610251
H	6.69226432099788	10.92423816506249	3.91667903325213
C	5.94146802712656	12.81912256139369	4.70292844770298
H	6.09534245532031	12.43575991777739	5.72255782876598
H	6.29782261814945	13.86028773702476	4.71184720567741
C	4.45029194617931	12.88668037012283	4.36546344476706
H	3.96887032571262	13.60541347026248	5.03987031013590
H	4.28653470556002	13.25742282656362	3.34618301690733
C	2.90855986942747	11.36261990387647	5.61952675714075
H	2.85256290686170	12.04452998085416	6.46125473631797
C	2.27321744588912	10.17748011918071	5.40990585648995
H	1.56916483386728	9.64272617942011	6.03546692413459
C	3.57340878357730	10.62808870250795	3.58951061432030
C	2.33879133806830	8.58125216647500	3.43677037945609
C	1.43508504223813	7.65143002291643	3.89716322837648
H	0.95459486316273	7.78850841531291	4.86201095368336
C	-0.39432217620348	5.66155857710604	4.78841738357457
H	0.34627676399716	5.70439970050925	5.60045773359645
H	-1.01070395361613	4.77188521503591	4.93838990362010
H	-1.04833496249355	6.54535292212659	4.83910716837444
C	-0.03493391591209	4.42502007665899	2.61737473109733
H	-0.43645059447960	4.76009596333723	1.65009798091720
H	-0.78826470186568	3.80097372418585	3.10405493405624
H	0.86448049588394	3.81472932530089	2.44726910092251
C	1.13484520898581	6.51610099254611	3.08452863500462
C	1.81037441783294	6.45185041545460	1.82742101894450
H	1.64281161008902	5.62664539598309	1.14006770214498
C	2.69643683176853	7.43973773388758	1.46935470859935
H	3.21273289612958	7.39398868346412	0.51114614800784

CCSD identifier	E	charge	Spin
22717110	-2438.665404686801	+2	0

Fe	-0.81721389048564	6.48358148818689	8.76635375828988
N	-2.36132799813685	5.57455527694401	7.62406952586142
H	-2.43135366305279	5.83420409148422	6.63427107068688
H	-3.28194733396429	5.77524011925157	8.02990583564731
H	-2.27592090348717	4.55286945381016	7.63654380534569
N	0.30838732771501	6.30422437692652	7.04459090261661
H	-0.11788520520600	6.84237256463275	6.28107666122731
H	0.40287435911945	5.34247050499554	6.70332829051701

```
H 1.26019922478316 6.67315370038525 7.13218373801522
N -2.12882912982623 6.45773856330242 10.23736489651430
N -1.27408314743631 4.35403975951098 10.46369503981386
N 0.67840985615169 3.95073772098757 9.61303241246728
N 1.65844142847126 7.25614578097209 10.39604285609989
N 0.64713066304523 8.78351553645558 9.24586099701348
N -1.29159477038504 8.35684858378486 8.11182296524777
C -2.98700973633687 7.44176745580391 10.59500820351112
H -2.84189413711658 8.40210334180236 10.10463115055182
C -3.99228870738035 7.25520438459633 11.53430140351015
H -4.64791878242049 8.08615068645605 11.79087193363506
C -4.14259204206486 6.00163463899157 12.13276346372408
H -4.92991074077015 5.82403853408381 12.86507897058613
C -3.26648437508464 4.97685553494503 11.78616655663610
H -3.34832726540468 3.98776404174764 12.23491215663298
C -2.27311285207264 5.25095896434675 10.85203259769833
C -0.34683147556366 4.85141507165438 9.55897309312139
C -0.82578438072049 3.17578277234138 11.06246206102437
H -1.40296008392722 2.61086500331368 11.78427290301080
C 0.39654606135942 2.93044222226384 10.52778433881254
H 1.08207548770570 2.10900588003839 10.70520600505552
C 2.03951184683301 4.30213650815604 9.20627773395493
H 1.96207553411176 4.97189509627647 8.34565834363160
H 2.56613062339801 3.39686331955412 8.87759894061563
C 2.80025085164117 4.98102515571539 10.35709775231829
H 3.10173848032289 4.21831857836864 11.09093855049794
H 3.73064103700105 5.41234618085052 9.95839701574990
C 2.00505370346630 6.03395427395635 11.13322886066489
H 1.07371699473733 5.60936882770443 11.52917415238777
H 2.59560421620828 6.35171620134042 12.00182571049274
C 2.38520472025039 8.44107303319952 10.53224551072215
H 3.28098097135394 8.49929412130024 11.14012002503404
C 1.75636744585785 9.40371003521274 9.81444168629434
H 1.99977498960581 10.44976172103631 9.67732494236116
C 0.56736144595865 7.44358566498863 9.59697578150808
C -0.37610200188606 9.30234526717960 8.45064180199463
C -0.45984821459790 10.63633938067033 8.05899246860011
H 0.30397408347663 11.35305376392805 8.35675892335442
C -1.54631524429200 11.02769356787428 7.28269454196735
H -1.64375541578327 12.06416975146634 6.96010026818044
C -2.50772855515018 10.07613524625551 6.93126835879880
H -3.37689497489907 10.34315691761318 6.33179252078838
C -2.34225560439313 8.76721270946439 7.36226712307804
H -3.08587972072941 8.01016162387279 7.11457239412979
```

CCSD identifier	E	charge	Spin
22717110	-2438.215947505930	+3	1/2

```
Fe -0.84369653302367 6.47069740711365 8.75453187702229
N -2.37045419641200 5.53147415667441 7.63028995585347
H -2.41605049418517 5.76240984194090 6.63072872357983
H -3.29941962937581 5.75059919017845 8.00878763031728
H -2.29851446509435 4.50780976100051 7.67043577597393
N 0.28826307610805 6.32603031137880 7.03894349440264
H -0.11924067938706 6.89353667756614 6.28555876810737
H 0.36371882767529 5.37038338221328 6.67118274670640
H 1.25032672699998 6.66585726406795 7.14139254809335
N -2.15383987281050 6.44845300858153 10.24075097521670
N -1.25159655468133 4.37175780751553 10.52870706027849
N 0.68050943665422 3.95281671834503 9.64056135008568
N 1.65811181278345 7.25139260264796 10.41834588642348
N 0.61877791712945 8.78200080658744 9.28807460373449
N -1.29876781135444 8.37583123485404 8.08869935327735
```

C	-3.03008682954783	7.43231869321026	10.55820969014482
H	-2.90869507376082	8.37736193447863	10.03343827218430
C	-4.02755776689987	7.25566297600619	11.50760086767002
H	-4.70094371795451	8.08101072707759	11.73819737022266
C	-4.14963477027366	6.01958432503648	12.15057725442473
H	-4.93270123739016	5.85335460779048	12.89163348721983
C	-3.25449947978500	4.99771318687515	11.84037849219876
H	-3.31886655332957	4.02469408782378	12.32683985983045
C	-2.27094271855842	5.25994720962618	10.89478320701406
C	-0.34071610321859	4.84496880558214	9.60718050920694
C	-0.81458127539383	3.18677475083068	11.10403638602656
H	-1.38163311178308	2.62343404588833	11.83702911918810
C	0.40281733917284	2.93087239295353	10.54448104014108
H	1.08578799881079	2.10335566758468	10.71212043283931
C	2.03740701799201	4.27780722873307	9.17955212822580
H	1.94243485489007	4.91486575937351	8.29622636185195
H	2.54170557525817	3.35486673502387	8.86702964422722
C	2.83026944808212	4.98073749427580	10.29544586438319
H	3.18563565098374	4.22821725208170	11.01573256354516
H	3.73392716683551	5.42690399363034	9.85421192765664
C	2.05871402444217	6.01313089025092	11.12135360618911
H	1.15449715069633	5.58256717331473	11.56979006486682
H	2.69200082694541	6.33554641530611	11.95697052701887
C	2.36049270433149	8.43790863385712	10.57113442199150
H	3.25895770410348	8.50525871984511	11.17671089896615
C	1.70561287345057	9.40803628500491	9.86976371672858
H	1.92649310124289	10.46439643471850	9.76581086967105
C	0.57248122820914	7.44538573532386	9.62241913571851
C	-0.39891685908146	9.31359161182676	8.46811624863854
C	-0.47008319462462	10.64922266958974	8.09133513263839
H	0.27493990848007	11.37105474446166	8.42404640822367
C	-1.52822528106441	11.04282344547427	7.27135392428433
H	-1.61677884891151	12.08325155191748	6.95566795224976
C	-2.46788055826084	10.09216048113666	6.86750380533455
H	-3.31176242075272	10.36236684181546	6.23282595478423
C	-2.31940695434696	8.77793681185452	7.29620456839980
H	-3.04999138001499	8.02085950975382	7.01229953702126

CCSD identifier	E	charge	Spin
2271711	-2706.409077855387	+2	0

Fe	2.20639311547831	-1.16995492605854	9.10659760025307
N	1.24171099141988	0.25610634251685	10.35070837116558
H	0.85128952064410	1.02593152084712	9.79845721746529
H	1.93622123058542	0.68482465267410	10.97145102306743
H	0.48198429030615	-0.07137555120808	10.95621109435681
N	0.33501509556435	-2.02122027241801	8.91943246853646

H	0.01499252041552	-2.33636575408634	9.84260713624182
H	0.31761771015767	-2.85830242560618	8.32940259945635
H	-0.38774407499773	-1.39343597774416	8.55449792546151
N	3.83439942880309	-0.10132125600874	9.47017187754362
N	6.82605630289279	2.76070628940429	10.20996637404153
N	3.00479176488373	1.06808961355167	7.69941701252576
N	1.58644593270500	0.07730245081791	6.39084986771518
N	3.33444009321915	-2.95956460652336	6.90358724791631
N	3.18691238845873	-3.75379410840781	8.91149050076955
N	3.11806219777700	-5.59842987276819	13.41706585602699
N	2.49239915494426	-2.46945001354654	10.66708366274339
C	4.75824986329617	-0.26840692954197	10.44952947683646
H	4.67276097280837	-1.18765888115568	11.02653101440114
C	5.75975264726185	0.63732120824709	10.72198956955095
H	6.46179633379493	0.41015832094425	11.51994944562197
C	5.86695029669905	1.84085543666523	9.96556457705213
C	7.81032681653798	2.53807687907632	11.26706911612475
H	7.32589482060319	2.44642052999858	12.25114232536411
H	8.49167008874175	3.39244552351706	11.30171531457685
H	8.40548709851329	1.63224128397639	11.07510719346179
C	6.89951660150507	3.97761885046280	9.40706264386219
H	7.08447459234597	3.74732574888511	8.34584560277012
H	7.72570236123408	4.59593784384274	9.76843704217058
H	5.97252512414321	4.56622336413229	9.48976938921478
C	4.90578194608749	2.01513568804215	8.93317810972288
H	4.91445940058959	2.89649427132779	8.29761920045005
C	3.95352352214501	1.03646611834019	8.73145478707243
C	2.15893838363679	-0.03179074070145	7.63086435196395
C	2.95891363015939	1.82486616877583	6.52973751954271
H	3.52723951253258	2.73465424426457	6.38210754012146
C	2.07392293082589	1.19888461313703	5.71316159008626
H	1.73332850702144	1.46494615588661	4.71871931455531
C	1.01544920071367	-1.08007200382513	5.70627254588018
H	0.23731464872112	-0.74444193284430	5.00810056081080
H	0.53398790475977	-1.70430938213409	6.46398784914553
C	2.10136509975935	-1.87395458323035	4.95994428184313
H	1.68071271553061	-2.84171480243917	4.64773885349541
H	2.36812625954340	-1.33918019496120	4.03550907335918
C	3.40719823721147	-2.07911549996505	5.73319741807070
H	4.14896330063616	-2.52147311589405	5.05590042449921
H	3.82067977335745	-1.11750941004753	6.06336856637351
C	3.75164439172696	-4.29083014177021	6.86380121937308
H	4.08715298966613	-4.76080201741127	5.94647350387021
C	3.66213623462766	-4.79380242603577	8.11995288996309
H	3.90017389745161	-5.77923789268467	8.49905330118134
C	2.97505995694999	-2.59678532615590	8.17391959910940
C	2.94983866163670	-3.69387010302504	10.29065179964409
C	3.17327565200726	-4.75700715033276	11.14434013900601
H	3.54344914236717	-5.69804108890409	10.74756557366882
C	3.60635566039433	-6.89686320157465	12.96146469331211
H	2.91061864670909	-7.35488071422083	12.24115926376513
H	3.69308385363516	-7.56713644667622	13.82081508461842
H	4.60029082318036	-6.80993870666025	12.49498960964374
C	2.85195595029328	-5.39121451856595	14.83964648841420
H	3.49722265156068	-4.60307699056125	15.25690199124537
H	3.05730946013831	-6.32020851223418	15.37828251777325
H	1.79927847541009	-5.12110635505735	15.01266719832614
C	2.92165270627537	-4.59620684797683	12.53369724393253
C	2.45550650355221	-3.31080118161968	12.93726347339325
H	2.24638305489455	-3.08764461124275	13.98014142419577
C	2.26537553574131	-2.32290052806782	11.99694297243703
H	1.92153549780816	-1.34053311744057	12.32219547983962

CCSD identifier	E	charge	Spin

2271711	-2706.002004446087	+3	1/2

Fe	2.25562023482693	-1.14531955187873	9.14442141598866
N	1.24284297680864	0.27902439852376	10.33963932791475
H	0.82211995137799	1.02656807312082	9.77675108320162
H	1.90809606792284	0.74679384348103	10.96441776339248
H	0.49249827201334	-0.07341127752943	10.94422458161488
N	0.38793596926302	-2.01362665000857	8.97698908976164
H	0.08244526764278	-2.35071984764984	9.89756434106526
H	0.36537897821615	-2.83605001937806	8.36616789296227
H	-0.34416937680298	-1.38270312265228	8.63335164851278
N	3.84959966035991	-0.07789183235790	9.47374616324052
N	6.84164354296452	2.77576962934034	10.19952022064746
N	3.05715013687136	1.05616294150595	7.65791306225240
N	1.60974042202014	0.06624517390653	6.39163523325295
N	3.32360280989567	-2.97880703565288	6.88828830779463
N	3.21547000968957	-3.74670037031486	8.91015028924218
N	3.09637714221690	-5.61118122699971	13.40927112579049
N	2.55368823955445	-2.45081865963923	10.68811463126491
C	4.74726843382541	-0.22198936919161	10.49063027349873
H	4.64263098736805	-1.12282555433290	11.09120014765309
C	5.74495504278731	0.68280379983492	10.75570710783729
H	6.42841250177601	0.47394273017870	11.57495470033401
C	5.88303314926148	1.86487668489088	9.96161914778344
C	7.79618786961147	2.59022011226950	11.29945878560138
H	7.27496029038047	2.54419840010411	12.26649865596528
H	8.48236597272635	3.44018686311953	11.31826539078481
H	8.38442379881093	1.67244858842553	11.15499746712039
C	6.95787026001562	3.97337223094713	9.36149881512125
H	7.14269884309843	3.69925126740335	8.31223001067791
H	7.80057583967076	4.57438422934656	9.71143057468574
H	6.04675154683550	4.58675529187555	9.42676362455377
C	4.94393454301648	2.01770014674629	8.89844932837237
H	4.97197455769850	2.88440277513336	8.24316159402447
C	3.99387226296290	1.04469033713644	8.70098576059351
C	2.20061101063336	-0.02311226559588	7.61147763035548
C	2.99253526883916	1.80747715644901	6.48741366882073
H	3.56687191230246	2.71016628027254	6.31733050432187
C	2.08376015366945	1.18048900975090	5.69531036088448
H	1.72412387786890	1.43845813027537	4.70460343065000
C	0.97731611765638	-1.07943395714756	5.73164694738424
H	0.19825641926273	-0.71817225742285	5.04843275073643
H	0.48435629255874	-1.67632094457204	6.50354606303361
C	2.02220184867844	-1.91259660118384	4.96888394966498
H	1.57145399686238	-2.87770020359249	4.69380364662364
H	2.26531271472650	-1.40935821764532	4.02079870936695
C	3.35718848827046	-2.12086784558459	5.68810794223565
H	4.05941548835552	-2.59817928448669	4.99362387305550
H	3.80861322074135	-1.16440864345080	5.97942156588878
C	3.71680454816433	-4.31016316024412	6.85330682991613
H	4.02548389822400	-4.79799369813654	5.93474158179647
C	3.65299870392894	-4.79725317541087	8.12393158593747
H	3.89428488178495	-5.78152194582431	8.50636482292063
C	3.00819965065243	-2.61064899543778	8.15947878117104
C	2.98410369471754	-3.68170263230331	10.30445112901262
C	3.18592285423827	-4.75314989967984	11.14430069923298
H	3.53677236245648	-5.70029908412968	10.74379204738769
C	3.55102108894011	-6.92682462465416	12.94912716383105
H	2.84416908565502	-7.35557619646721	12.22324865971093
H	3.60893750421736	-7.60250847222798	13.80592291784115
H	4.55184055741969	-6.86228245095773	12.49633436684355
C	2.82534186250249	-5.41576734211224	14.83846255969010
H	3.49367097518529	-4.65305123465830	15.26432878053651
H	2.99872832486485	-6.35766153941497	15.36445235523293
H	1.77954183467368	-5.11704787069846	15.00104992230863
C	2.93130984712225	-4.59982394468235	12.54172564616339
C	2.49554428004253	-3.30262796782460	12.95366217635875
H	2.28933680671650	-3.08264240638336	13.99804965668340
C	2.32984922381044	-2.30476951726519	12.02413072272613
H	2.00746199956995	-1.31757919725696	12.35621698916827

CCSD identifier	E	charge	Spin
2292272	-2669.500831323870	+2	0

Fe	3.82188112391897	5.15467736291153	2.56593846395361
N	5.03983666816233	5.51444494387874	1.08962141513680
N	3.63752964042005	7.02754790143490	2.75954431125321
N	4.12075731731645	3.22563444334053	2.14351833823477
N	2.43320253787462	3.68455251919727	0.67567389596887
N	1.19984362200068	5.42795995068527	1.02289806053277
N	1.45083229286845	4.78766382713101	4.49558044842021
N	3.43342349276551	5.03726459035633	5.31340566276042
N	5.28442636606716	5.23608070132707	3.98515264055511
C	5.71045944928587	5.73395491404981	0.16633559572081
C	6.53882765860656	6.00636399768569	-0.99058309898048
H	5.90835296394698	6.24031003418465	-1.86041768417383
H	7.15830585123940	5.13005128690078	-1.22860180972730
H	7.19867998129193	6.86303766065355	-0.79304127565020
C	3.65130220355609	8.18282535597764	2.89371215812972
C	3.65703027030394	9.62364229176648	3.04719276788233
H	3.32616999195681	10.10782669162391	2.11709786914664
H	4.67077555618284	9.97620727108862	3.28563259662565
H	2.98194336257687	9.92346411373394	3.86147972587346
C	5.04476335301339	2.40287926243283	2.68209028182423
H	5.59905974018855	2.80178582894614	3.52995450320302
C	5.29933881034120	1.11398757807597	2.21224866409158
C	6.32305497312395	0.23793267015276	2.87100467268903
H	7.06242203115109	-0.12062513661565	2.14078449605821
H	5.84953065849129	-0.65130767607783	3.31260199374865
H	6.85803241944014	0.76782334272924	3.66889370208892
C	4.55462233563967	0.67875887777410	1.10433605885891
H	4.72387389844831	-0.31642378016818	0.69012667592810
C	3.59741659547518	1.50783876414912	0.53303116141494
H	3.01104596469906	1.18247173210783	-0.32528286637061
C	3.40457964313914	2.76726251922485	1.08956376912542
C	2.37974713084544	4.86364314248414	1.38876598164111
C	1.30244201396628	3.52235285000818	-0.12356594158079
H	1.14937650019669	2.67586292708249	-0.78138929497002
C	0.53082440136826	4.61705974371493	0.10119982926638
H	-0.42562329443239	4.89693577305039	-0.32584422481776
C	0.53829388529910	6.42990416047820	1.85861099418970
H	-0.07995438868253	7.07872825846334	1.22481812100141
H	1.32717479203292	7.03944342575595	2.30568821741977
C	-0.32421583094321	5.75574668048842	2.94100539453965
H	-0.59890519556014	6.51103237618934	3.69229058021599
H	-1.26684779074140	5.40658924214048	2.49243366467145
C	0.30107933065523	4.53195151079598	3.61700122945827
H	-0.46105170046454	4.04966740518912	4.24200152894298
H	0.60914299335358	3.78673213608471	2.87271693015604
C	1.33098803090632	4.83376482874223	5.88582597153915
H	0.37342200583986	4.73316230173646	6.38351876368691
C	2.57437699435975	4.99060988204763	6.40528321799017
H	2.90346251539346	5.05892694024047	7.43434795492997
C	2.75202463649411	4.91609640054631	4.12108861390699
C	4.82375269621121	5.19242226386524	5.25368181118118
C	5.65328778732530	5.28433434759515	6.36729698986974
H	5.24437046475266	5.24161953134649	7.37564829787445
C	7.01876066339007	5.42852773106322	6.15502582088560
H	7.69271349169960	5.50107973408556	7.01019222716143
C	9.00079095665795	5.63745347195292	4.58290235824350
H	9.21987800283129	5.64350853065546	3.50796126726332

H 9.57329469474958 4.81932804763885 5.04344105900410
H 9.37957669942867 6.57573033688375 5.01370109391972
C 7.53317350121197 5.48077108401524 4.84906723911231
C 6.61382539900244 5.38122904676777 3.80394369028976
H 6.94529983935985 5.41579104823253 2.76634641868444

CCSD identifier	E	charge	Spin
2292272	-2669.065000516609	+3	1/2

Fe 3.82976119027852 5.12396622253644 2.55672735978976
N 5.05547227639708 5.48185292266945 1.03136956281028
N 3.68276627329080 7.03453271594127 2.78209770308773
N 4.11458922626116 3.20144686282788 2.13798416730615
N 2.39425817052008 3.64511689166371 0.70548744327708
N 1.18462293724804 5.41234323837121 1.04896783211654
N 1.43431388034683 4.76210631723221 4.49685761211152
N 3.42582784623818 5.01655840698644 5.31015738398895
N 5.29714015257330 5.21452929683200 4.00053025489398
C 5.69854595256485 5.71761807266312 0.09371888561474
C 6.49955677699918 6.01352025909884 -1.07079356783017
H 5.84591520702341 6.29657533869237 -1.90997060221384
H 7.08510702814092 5.12820726111185 -1.36052788417707
H 7.18611335369027 6.84620995325351 -0.85788133236157
C 3.72674819227064 8.18789747501495 2.90963719927547
C 3.77026262446722 9.62449421790936 3.05892703060465
H 3.36411956294188 10.10943986151561 2.15843091128677
H 4.80860213856514 9.95750670328504 3.20479394556350
H 3.17251248939862 9.93267059734821 3.92978019007888
C 5.05585819593914 2.39138148403240 2.66902709931835
H 5.62414541468091 2.79718468512285 3.50343720790280
C 5.30447763846175 1.09748709434003 2.20207217190638
C 6.33828783001415 0.22735413560348 2.84422250653977
H 7.07058129878933 -0.11976550145546 2.10040948391962
H 5.87218662307268 -0.67319322523707 3.27223606674747
H 6.87697298569998 0.74744262869059 3.64518877208881
C 4.53848325135944 0.65237024972104 1.10848339597362
H 4.70387149761415 -0.34610370137438 0.69948522292759
C 3.56598972555675 1.47050287465283 0.54771782791341
H 2.96402286745736 1.13222362300072 -0.29523026894100
C 3.37887090779245 2.73145838371389 1.09937254100933
C 2.34583405971782 4.82217414994937 1.41684157045150
C 1.29258747953738 3.52039358684502 -0.12266184948695
H 1.13046271205925 2.68396180103280 -0.79316389395196
C 0.53117653237409 4.63412495920972 0.10231906607568
H -0.41583219093640 4.92967546646169 -0.33854520346217
C 0.53232909774329 6.43870712218363 1.87243232685375
H -0.07809777785849 7.08157046180271 1.22631086730377
H 1.32770021008841 7.04820258360355 2.30879471689201
C -0.33527482251193 5.77537109439069 2.95730638378397
H -0.59453061415676 6.53530196729149 3.70897211311716
H -1.28736206099734 5.44575307537325 2.51422134917349
C 0.26191161607262 4.53508196209887 3.62804463336351
H -0.50381989024460 4.08188573282098 4.26946706277772
H 0.53851049666687 3.76866520355618 2.89333757317053
C 1.32186628563085 4.80865947602198 5.88060161447190
H 0.36624064977106 4.71093455787161 6.38503513272044
C 2.57353793818575 4.96080780384200 6.39798370197888
H 2.90422616612026 5.01217007587728 7.42868889124216

C	2.73450495376930	4.89256964706802	4.12972008003088
C	4.82454209709032	5.17971484748607	5.26162383076823
C	5.64058827523998	5.29881599019797	6.37989453167897
H	5.22895391104742	5.25988279400482	7.38765515782671
C	7.00586696281352	5.46980214805521	6.17438261841339
H	7.67053632636245	5.56394277473822	7.03520287833301
C	8.99756096751799	5.70253848427803	4.62007339250118
H	9.23415532259797	5.70614373455176	3.54935882707236
H	9.57758480878006	4.89971116276681	5.09861048507775
H	9.35126596355650	6.64942605529968	5.05448545996138
C	7.53319986868288	5.52146384767475	4.87268154362453
C	6.62292515499995	5.38906070434201	3.81850769439534
H	6.96266598462504	5.41444938353904	2.78360132131116

CCSD identifier	E	charge	Spin
2295151	-2517.227793223763	+2	0

Fe	6.89970802823497	4.48942321100790	8.63804366430442
N	8.16391391913255	5.79804059463632	7.54553115147846
H	8.02081968603600	5.69055613395052	6.53607443557600
H	9.14920921853858	5.56396893159126	7.70858502937594
H	8.08030380853570	6.80265773196809	7.73384709347752
N	5.48858877711126	5.98489474187108	8.44907717850546
H	5.27091349127371	6.23681391401271	7.47993508636509
H	5.81719277749509	6.84288016246973	8.90725480769759
H	4.59390328834768	5.76735446549929	8.89830347480658
N	8.43677764401030	3.25381985128667	8.59748462731809
N	7.48793745420088	2.73889336145368	6.58612445891566
N	5.39997429222825	3.23285841275768	6.28113954208209
N	4.78915856992571	2.53723128947672	9.69664179531928
N	5.69730449148001	3.94003638755826	11.06906269398577
N	7.38280683548710	5.34566236356512	10.42657496498668
C	9.40631364064329	3.11434654078492	9.53224470350382
H	9.24198452515509	3.64840299592811	10.46683446180082
C	10.55025403867812	2.33710025733917	9.35189248187322
C	11.57479072287451	2.20373219104259	10.43889299092929
H	12.57021459534928	2.50238121701106	10.08011655621307
H	11.65391741122193	1.15856440384707	10.77228116662074
H	11.32489472834355	2.81971401565130	11.31157207187908
C	10.69450219089062	1.68464319899262	8.11572808070417
H	11.57792261797369	1.07505996066614	7.91990229638034
C	9.71196307672067	1.80596631558917	7.14112464859473
H	9.80750998017696	1.30138605189351	6.18042437789737
C	8.59606428539312	2.58533734027342	7.42578505488783
C	6.45925298195016	3.51569952338862	7.09689275457022
C	7.06941889182349	1.99153588793594	5.48517677062596
H	7.72096421354127	1.33195526150917	4.92511251630562
C	5.76174331784136	2.30319697718370	5.30077930329543
H	5.06288056651443	1.95909263326323	4.54637224514636
C	4.01661993023617	3.43734629508867	6.71003432302764
H	3.38371525241468	3.60224123468348	5.82839721494831
H	3.98869243539190	4.35022071523310	7.31145102596112
C	3.50561131645254	2.23371343356671	7.52028348394349
H	2.55564222322350	2.51058705648071	8.00155131018481
H	3.27378924590059	1.40793330850786	6.83055125590467
C	4.48896212066977	1.67133558849844	8.54971175493557
H	4.07414856122815	0.74247404914929	8.96123302131856
H	5.44234350203570	1.40398215806158	8.07669196505107

C	4.17704363493842	2.36975246143210	10.94028478356009
H	3.40032103980997	1.63146397837978	11.10390684944207
C	4.74262527334928	3.24644856222244	11.80650852147973
H	4.55116692889077	3.41755556715466	12.85832845986285
C	5.74313826579588	3.51310650086277	9.75103777112112
C	6.60576315524189	4.92850949008884	11.45558757009591
C	6.72002273870130	5.42741698539877	12.75075460211271
H	6.07247804081465	5.06390659063093	13.54738652949986
C	7.68272236842695	6.39625724251292	13.00114085024403
H	7.79256563403445	6.80210528225654	14.00797111418605
C	9.57526891872482	7.88659384733631	12.19711808764840
H	10.14630517642698	8.09816826637216	11.28452186433319
H	10.28122655624040	7.55953766781819	12.97407424656743
H	9.12882249657245	8.82899066863689	12.54657403394075
C	8.51773522660887	6.84934767773777	11.96510753722722
C	8.31716716580428	6.28582923905194	10.70549739028566
H	8.95359672493612	6.59416980743231	9.87507994769469

CCSD identifier	E	charge	Spin
2295151	-2516.786815697090	+3	1/2

Fe	6.93616650997066	4.49307856127465	8.62378556520232
N	8.20394803553893	5.76074963689133	7.50484654657099
H	8.09897733531216	5.60785975842492	6.49502100499726
H	9.19006530707622	5.56108546242664	7.70837107075879
H	8.08869048228532	6.77146460313641	7.64312968026105
N	5.54066006097754	6.00189575496781	8.47330579419182
H	5.33355519274967	6.28433969084747	7.50833981408324
H	5.86893334642460	6.84550934862883	8.95901310753150
H	4.63682065819181	5.77790835545321	8.90261573086037
N	8.45670467951064	3.22462958776258	8.58905757436907
N	7.46662855259898	2.66222257931297	6.60890165986781
N	5.39611517495654	3.21724088611433	6.28847667337622
N	4.79604383017564	2.51894123520790	9.69709333588263
N	5.74591634312694	3.90233102166555	11.06985342846749
N	7.40961162332093	5.35908831203376	10.44262874478251
C	9.43572120988429	3.11192071165672	9.51889430216947
H	9.29255224018160	3.67909887428258	10.43693186735493
C	10.56870038941941	2.31291075699782	9.34621660269082
C	11.60867387623938	2.19710407561772	10.41515797019149
H	12.60002747010819	2.47052704178648	10.02426220494235
H	11.68448642938882	1.15651400064860	10.76585146373422
H	11.38775213059153	2.83438332624329	11.27958133583167
C	10.68327259133693	1.61966697576500	8.12519095072883
H	11.55766659231889	0.99388608007257	7.93599769888037
C	9.68939172454899	1.71776954712291	7.15927185500000
H	9.76858136419909	1.17927633390262	6.21512041512745
C	8.58791757049508	2.51843876008767	7.43544642665340
C	6.45823514123230	3.46695757526385	7.09566026941703
C	7.04535369686283	1.94488822505329	5.49820368035360
H	7.68104490835680	1.26707181002164	4.93935495766473
C	5.74129919141777	2.29452662167370	5.30462631731314
H	5.03807220309535	1.96715410335959	4.54488277652347
C	4.00971541194203	3.48183247956569	6.69429923162758

H	3.39729296462263	3.63971512860846	5.79768345717672
H	4.00138702768390	4.41595296869545	7.26284767826337
C	3.45863776014735	2.31013096868213	7.52759314250913
H	2.53384948723583	2.63873645799042	8.02451827709605
H	3.16477514037523	1.49351820768537	6.85069901920385
C	4.42625483034089	1.68344400014242	8.53515023763071
H	3.96072197737680	0.78128008707022	8.95047604385492
H	5.35708537752111	1.35706717520576	8.05494880211334
C	4.21386669790739	2.34161821353035	10.94306530434548
H	3.43178975478525	1.60951656674808	11.11777417723653
C	4.81442122631967	3.20400048214692	11.81422433228011
H	4.65504431943010	3.34743634850473	12.87687621697201
C	5.75049359928866	3.48652346873122	9.75660125292856
C	6.64838027197657	4.91239406823035	11.46466204856313
C	6.74690008954284	5.40585632865042	12.76056689056016
H	6.11854820391573	5.01864291793862	13.56194194890931
C	7.67227900319511	6.41385863411412	13.00972915053387
H	7.76803889909407	6.81870497825691	14.01907838456075
C	9.49340580291008	7.98925411556350	12.20829995678669
H	10.05835342031908	8.23397992374541	11.30088803212011
H	10.20645303070854	7.69067783910140	12.99096190101365
H	9.00095348380440	8.90651235024485	12.56534842027683
C	8.48418653552407	6.91033145918711	11.97432281592075
C	8.30486562407941	6.33792747995057	10.71001548446179
H	8.92531319805908	6.67284873800491	9.87793496727414

CCSD identifier	E	charge	Spin
2320328	-2703.917127046502	+2	0

Fe	10.46234653776589	3.99989381099531	5.78362006852105
N	9.98566924652595	5.07640567518771	7.32514854257221
N	12.03686051787290	3.59039723301159	6.83116386155876
N	8.47427276244926	4.65211342479696	3.55260262682934
N	7.56138748101033	4.15990669492844	5.43527315759831
N	8.22254498402720	2.58231916501110	7.07899904809971
N	9.83631235371840	1.17940555752270	6.90319222165204
N	10.88162181769705	1.89314379715981	3.59109443486700
N	12.43077117708450	3.37525057529692	3.69773393042367
N	12.43150434726294	5.67154152003165	4.30756158254551
N	10.78574797801271	6.92110938893901	4.86821034705266
C	9.91781465183505	5.69891102441743	8.30686575154531
C	9.82651564438122	6.48428759627823	9.52227334524725
H	8.93756530936519	7.13138837228442	9.49671195582389
H	10.71717773084092	7.11955032416156	9.63366308761451
H	9.75355396916386	5.82548827386912	10.39948654162831
C	12.90632227574272	3.34058648900820	7.56447868189271
C	13.98796518416914	3.01934589326867	8.47581910911788
H	14.54773756797072	3.92700286050199	8.74354004476900
H	14.68209699475457	2.30505051457840	8.00992612599813
H	13.58945010600207	2.56853836152145	9.39616049860081
C	9.39872955048171	4.96823295688533	2.46994580081980
H	9.43337936638441	6.05276755954777	2.29981253126628
H	9.06307588005481	4.47266391658904	1.55111382094088
H	10.39574132358378	4.61192319026448	2.72830616903854

C	8.79451437735293	4.30717338432345	4.84047990709261
C	7.09853941228410	4.72246331220197	3.36866940237989
H	6.65581326077846	4.98151307779810	2.41400518850724
C	6.51912167812612	4.40607206313868	4.55345194297132
H	5.47589526806660	4.33544374876120	4.83851763596832
C	7.39449955517108	3.74417655633911	6.81582140815230
H	7.66070832610236	4.56492590081472	7.49079214908066
H	6.34545654031233	3.47207552319347	6.97568927536282
C	9.50436699639054	2.47364672978023	6.60352973521475
C	7.77059448771228	1.40099392487188	7.65368721313192
H	6.78983565669354	1.30208563313456	8.10461719476173
C	8.79230156910579	0.51627386853116	7.54182590100705
H	8.86809813911300	-0.51735466542372	7.85956879663170
C	10.90759349248412	0.44844534994158	6.23067129688807
H	11.25910000352625	-0.35385108184154	6.89257602212818
H	11.73705388297433	1.14437939346566	6.06478676073846
C	10.38835551317599	-0.12929035360541	4.90824543404450
H	11.20669081267819	-0.67492109682124	4.41448076793212
H	9.59117352204590	-0.86112668110423	5.10685077471906
C	9.83497612469742	0.94562286355627	3.96667200020580
H	9.44790976632701	0.48520110568357	3.04842100137073
H	9.01764792031436	1.51131372916050	4.42827630039634
C	11.79855266764302	1.59272453079009	2.59098451764809
H	11.67240945985786	0.74715464246774	1.92485656561480
C	12.77440902254126	2.53181879220339	2.65121782406489
H	13.66253344159694	2.67602906476026	2.04700323398661
C	11.25001632064459	3.00626550730476	4.30498217147457
C	13.24737621474287	4.48992079891853	4.13990475596559
H	13.73686122833695	4.23637389506111	5.08857714815413
H	14.01293372950104	4.68759740457064	3.38167111765317
C	12.71458677658057	6.92699370924055	3.79027325452822
H	13.61547835820904	7.14832933109734	3.22983981587797
C	11.67313250242159	7.71749210961101	4.15360566690620
H	11.48696254984114	8.76875741919064	3.96688457384051
C	11.24048024357638	5.63385499013700	4.98000088482156
C	9.52767116574737	7.45370166810877	5.37795727370504
H	8.87548579795322	6.62569827457721	5.66017432157241
H	9.04241535533903	8.05089191632324	4.59593252450433
H	9.70808210187677	8.09361048368026	6.25189395497258

CCSD identifier	E	charge	Spin
2320328	-2703.496115286613	+3	1/2

Fe	10.44942009022481	3.99784963509963	5.75276394305508
N	9.98287150840171	5.16831067727688	7.28859256089437
N	12.02805829628758	3.52528303997780	6.85472308112545
N	8.49122109795507	4.67382995166540	3.49620985057768
N	7.55381262924681	4.28794076735846	5.39817484853029
N	8.13128613850632	2.70669715689405	7.07429644427337
N	9.68409953466854	1.22470212726038	6.94785245670961
N	10.86675794113149	1.78744038176383	3.65626477392597
N	12.45056775636362	3.23892510206505	3.72466263698749
N	12.50971164594865	5.55755265094399	4.24996568105643
N	10.90825481242166	6.89425853031605	4.76125829099109
C	9.94282019784537	5.81724877490947	8.25235640898426
C	9.87997587949026	6.62935903113899	9.44784578513890
H	9.04463992112540	7.34280871715054	9.38366877898107
H	10.81670304227615	7.19391714933119	9.56895738190677
H	9.73460993521494	5.99206316262743	10.33275459974670

C	12.87843463095366	3.27164016981258	7.60531592762812
C	13.93742631676261	2.95142679402090	8.53711237348982
H	14.49751391642295	3.86159960375492	8.79922135078289
H	14.63217032719684	2.22341925939305	8.09267453452526
H	13.51243410035899	2.52216808112243	9.45670781066460
C	9.42970765189122	4.89711461249223	2.39577401430104
H	9.00002072508896	4.48445095151884	1.47603938081504
H	10.37334206616637	4.39541186407466	2.61068763762806
H	9.60267370722989	5.97197610914258	2.25565405055581
C	8.78334434875920	4.35037023846053	4.79103115954092
C	7.12690578833021	4.82907713329996	3.31691824555899
H	6.69428421853727	5.08930405031254	2.35673722019077
C	6.53054505702008	4.57775035961005	4.51408509161514
H	5.48334436360891	4.57255038500278	4.79726474111842
C	7.35242969649134	3.89929492102260	6.78548507519181
H	7.64235828672512	4.71812722721172	7.45288052773804
H	6.29152540428956	3.67236615074252	6.93576089066384
C	9.40067878098056	2.51342899915825	6.60068693790043
C	7.64514309446704	1.58488086443717	7.71836199326903
H	6.66203750084759	1.54062080665256	8.17547373950881
C	8.63272878636817	0.64807888316024	7.63803914865126
H	8.66218710510128	-0.37400671592148	8.00120205115133
C	10.74576451967104	0.41930404857288	6.33552478644866
H	11.03004841205242	-0.37437735077036	7.03761438879552
H	11.61568816914054	1.06639435363632	6.18018893551854
C	10.24098257821099	-0.17517244857569	5.01553773291868
H	11.04993089484343	-0.77405783647794	4.57149379399753
H	9.40648025571256	-0.86597852086148	5.20640987422853
C	9.76737893061937	0.88887704802533	4.02026428689379
H	9.40397660505475	0.41344436111483	3.10045777394665
H	8.94879069742180	1.49592197927007	4.42344175985741
C	11.79716322483873	1.41949960809041	2.69202656210769
H	11.66383897822504	0.54582969136058	2.06340438357673
C	12.79531559637085	2.33681316718704	2.72861587324837
H	13.70179833736450	2.42721877893538	2.14002069742823
C	11.25479123455703	2.91891663273318	4.31521573283727
C	13.29668594503958	4.34567584917880	4.12939741178067
H	13.77963696003743	4.11942576955686	5.08840411842077
H	14.06977977760796	4.49679915527445	3.36814923151425
C	12.87396870723496	6.79240987920849	3.75627607923163
H	13.80431213682926	6.97334859718535	3.22814231808273
C	11.85603254665192	7.63899144001079	4.08548108789476
H	11.73256146099928	8.69892593501758	3.88853927041321
C	11.30047575249184	5.58914082426867	4.88462838742359
C	9.66473285603193	7.50212527317356	5.23594163372964
H	8.96459401889903	6.71919041487794	5.52952022860768
H	9.22816366643167	8.10453844176553	4.43050264821169
H	9.87120037110205	8.15516744776566	6.09346776072321

CCSD identifier/Name	E	charge	Spin
Ferricenium	-1650.040273591985	+1	1

C	0.71357818325750	0.98172607031761	-1.66159678208529
C	1.13942417476010	-0.38449964855949	-1.74004970547950
C	-0.02164316196557	-1.20820357572391	-1.78534057919729
C	-1.16966924279107	-0.36789478446925	-1.72899438197763
C	-0.72405144752695	0.99152689072050	-1.65426279826554
H	1.36194364558995	1.85246125218018	-1.61858767762751

H	2.16831686178393	-0.73504393891736	-1.73575962299975
H	-0.02974801232071	-2.29544511761016	-1.79951187179432
H	-2.20340086962474	-0.70364460344408	-1.71220920342862
H	-1.35997170775462	1.87097577898866	-1.60301797947539
Fe	-0.00000762267629	-0.00598771658828	0.00000007749438
C	-1.13918024713329	-0.38500030040053	1.74299743994417
C	-0.71362419550003	0.98091224511293	1.66367374436699
C	0.72398382454932	0.99102614491331	1.65289425339686
C	1.16987055081831	-0.36877159117309	1.72578213154854
C	0.02157177563322	-1.20890568541755	1.78493129358469
H	-2.16814365126150	-0.73541943299226	1.73988584700491
H	-1.36228913724504	1.85148512027002	1.62164475678964
H	1.35961756394208	1.87064980717728	1.60133128727621
H	2.20351554929223	-0.70463741716793	1.70752044915849
H	0.02931472917317	-2.29615556821658	1.79868495376595

CCSD identifier/Name	E	charge	Spin
Ferrocene	-1650.287725698058	0	1

C	0.71681042556425	0.98735398501780	-1.63978347821983
C	1.16009884091174	-0.37649842743503	-1.64096106982110
C	-0.00009878430920	-1.21936019163507	-1.64143660517375
C	-1.16039260767719	-0.37662236635977	-1.64075323119308
C	-0.71723439984588	0.98728725690876	-1.63967667539856
H	1.35522850664889	1.86602984544477	-1.61222773631536
H	2.19317253108720	-0.71217505764335	-1.61684591536902
H	-0.00005355282997	-2.30556730627329	-1.61690599737206
H	-2.19345446098275	-0.71232395143591	-1.61638637657295
H	-1.35573012319634	1.86589696622715	-1.61176463500983
Fe	-0.00004833537291	-0.00099830711382	0.00000027889052
C	-1.16006844590047	-0.37651901818591	1.64100651461642
C	-0.71681192731384	0.98734647755118	1.63983945134793
C	0.71723461553671	0.98727176094862	1.63965785756526
C	1.16038268676544	-0.37663483416170	1.64069246185275
C	0.00011079699354	-1.21941286140854	1.64141703869155
H	-2.19314669287774	-0.71218514014603	1.61680066439415
H	-1.35523325492682	1.86601717451409	1.61222656287488
H	1.35572618841960	1.86588534152835	1.61178569470728
H	2.19344423032362	-0.71233566910811	1.61623982506498
H	0.00006491498212	-2.30562181823419	1.61707452243982

CCSD identifier	E	charge	Spin

Nitrogen	-109.51945795	0	0

N -0.690863000 0.072638000 -1.910262000
N 0.268831000 0.513907000 -1.637777000

CCSD identifier	E	charge	Spin
Hydrazine	-111.85116916	0	0

N -0.699448000 0.231034000 -2.322719000
N 0.154240000 0.623521000 -1.225281000
H -1.527502000 -0.150972000 -1.885540000
H -0.269830000 -0.516998000 -2.864801000
H 0.983165000 1.003496000 -1.662554000
H 0.442322000 -0.189557000 -0.683219000

CCSD identifier	E	charge	Spin
$[^{NMe2}(NCCN)Fe(NCCH_3)$	-2727.10135386	+2	0

Fe	4.227936000	10.075926000	1.830633000
N	5.150274000	9.130182000	0.373366000
N	3.118031000	11.079474000	0.534656000
N	1.301507000	13.245582000	-2.537387000
N	4.917930000	12.478138000	0.617767000
N	6.342116000	12.265224000	2.212356000
N	3.714062000	11.566737000	4.518775000
N	2.706178000	9.709527000	4.151291000
N	2.973950000	8.509644000	2.220427000
N	0.299999000	5.551322000	3.466253000
C	5.775184000	8.582333000	-0.418814000
C	6.570772000	7.890859000	-1.415427000
H	6.001394000	7.791662000	-2.340578000
H	6.836412000	6.897065000	-1.052337000
H	7.483841000	8.452442000	-1.617114000
C	1.917976000	10.724656000	0.038788000
H	1.463202000	9.851970000	0.483625000
C	1.280492000	11.407049000	-0.964000000
H	0.316200000	11.057184000	-1.294015000
C	1.888080000	12.550594000	-1.547373000
C	-0.010541000	12.846606000	-3.042864000
H	0.018566000	11.839468000	-3.464153000
H	-0.309796000	13.533467000	-3.827521000
H	-0.764550000	12.880680000	-2.253642000
C	1.958406000	14.417578000	-3.108308000
H	2.099422000	15.198484000	-2.356820000
H	1.337090000	14.820042000	-3.901243000
H	2.928376000	14.155960000	-3.537421000

C	3.152426000	12.919005000	-1.021884000
H	3.687648000	13.774488000	-1.398293000
C	3.695823000	12.170471000	-0.009182000
C	5.317842000	11.634296000	1.613292000
C	5.693027000	13.636544000	0.602116000
H	5.561862000	14.430395000	-0.109730000
C	6.584275000	13.501170000	1.607289000
H	7.372561000	14.157948000	1.930453000
C	6.773327000	11.879917000	3.554530000
H	7.843668000	12.051099000	3.658938000
H	6.598352000	10.809294000	3.643021000
C	5.999334000	12.663275000	4.624454000
H	6.189639000	12.205212000	5.597198000
H	6.395278000	13.679901000	4.679186000
C	4.493869000	12.802814000	4.385031000
H	4.088346000	13.504685000	5.111364000
H	4.289522000	13.215926000	3.400015000
C	3.026699000	11.232743000	5.681008000
H	3.044485000	11.855670000	6.557548000
C	2.391147000	10.063872000	5.453573000
H	1.762425000	9.478016000	6.097495000
C	3.525037000	10.634238000	3.559526000
C	2.347766000	8.562857000	3.412494000
C	1.459302000	7.628059000	3.875902000
H	0.998867000	7.754985000	4.840840000
C	-0.344674000	5.637216000	4.774456000
H	0.395787000	5.654642000	5.577248000
H	-0.974564000	4.765576000	4.916130000
H	-0.974007000	6.527324000	4.847516000
C	0.005587000	4.408417000	2.602244000
H	-0.429162000	4.733358000	1.654939000
H	-0.711440000	3.765646000	3.101640000
H	0.906306000	3.825025000	2.400845000
C	1.155403000	6.504008000	3.064165000
C	1.808442000	6.452606000	1.803383000
H	1.636740000	5.638976000	1.118068000
C	2.679617000	7.445808000	1.444779000
H	3.179336000	7.412497000	0.487561000

CCSD identifier	E	charge	Spin
$[^{NMe2}(NCCN)Fe(NCCH_3)$	-2726.68715094	+3	1/2

Fe	3.939430000	10.154343000	1.761237000
N	5.131457000	9.153646000	0.499540000
N	2.967679000	11.189678000	0.439023000
N	1.271296000	13.383928000	-2.659736000
N	4.809537000	12.526161000	0.557844000
N	6.218135000	12.204657000	2.148599000
N	3.726285000	11.537256000	4.566558000
N	2.696013000	9.680219000	4.202868000
N	2.804695000	8.587814000	2.189367000
N	0.376978000	5.472800000	3.518587000
C	5.866518000	8.573060000	-0.164285000
C	6.793972000	7.836806000	-0.995636000
H	6.307918000	7.563881000	-1.934636000
H	7.108537000	6.928871000	-0.477053000
H	7.669080000	8.453130000	-1.209383000
C	1.748349000	10.866038000	-0.068939000
H	1.256807000	10.015090000	0.378747000
C	1.157644000	11.560376000	-1.079761000
H	0.188177000	11.240324000	-1.425043000

C	1.815421000	12.685415000	-1.668694000
C	-0.064744000	13.045801000	-3.173406000
H	-0.057067000	12.068030000	-3.657725000
H	-0.356218000	13.791078000	-3.904800000
H	-0.799135000	13.051705000	-2.367963000
C	1.983891000	14.516234000	-3.265849000
H	2.096258000	15.332758000	-2.549983000
H	1.410354000	14.878801000	-4.111356000
H	2.964006000	14.205190000	-3.629053000
C	3.093123000	13.020309000	-1.122039000
H	3.652809000	13.864062000	-1.490391000
C	3.594721000	12.269513000	-0.103814000
C	5.131558000	11.686672000	1.573258000
C	5.710017000	13.585838000	0.493252000
H	5.657902000	14.368140000	-0.242068000
C	6.593077000	13.381257000	1.496934000
H	7.452484000	13.955997000	1.796816000
C	6.695932000	11.768001000	3.465185000
H	7.777901000	11.878687000	3.505289000
H	6.469702000	10.706655000	3.549607000
C	6.029424000	12.581485000	4.580684000
H	6.268422000	12.114052000	5.537744000
H	6.464111000	13.582900000	4.610863000
C	4.519245000	12.773766000	4.438356000
H	4.168808000	13.445874000	5.218653000
H	4.259246000	13.231594000	3.486760000
C	3.120232000	11.160784000	5.749958000
H	3.193183000	11.751798000	6.646803000
C	2.468615000	9.991586000	5.525030000
H	1.881512000	9.387048000	6.192183000
C	3.474161000	10.630042000	3.600869000
C	2.288909000	8.557476000	3.442972000
C	1.489416000	7.571702000	3.935205000
H	1.124826000	7.624025000	4.947291000
C	-0.152319000	5.460947000	4.888914000
H	0.660520000	5.457513000	5.616789000
H	-0.738642000	4.560344000	5.031026000
H	-0.800370000	6.321840000	5.060931000
C	0.042336000	4.350149000	2.629246000
H	-0.486951000	4.705306000	1.744406000
H	-0.603755000	3.662051000	3.162256000
H	0.944170000	3.815574000	2.328015000
C	1.147188000	6.468105000	3.091641000
C	1.687166000	6.504919000	1.769489000
H	1.482676000	5.716829000	1.063386000
C	2.481313000	7.540993000	1.382713000
H	2.894469000	7.570912000	0.384796000

CCSD identifier	E	charge	Spin
$[^{N}(NCCN)Fe$	-2326.37084796	+2	0

Fe	-0.876654000	6.639045000	9.049847000
N	-2.300968000	6.453999000	10.398518000
N	-1.337248000	4.385943000	10.595444000
N	0.594870000	4.037303000	9.726138000
N	1.788977000	7.349261000	10.386030000
N	0.731388000	8.862201000	9.281007000
N	-1.289485000	8.471929000	8.275563000
C	-3.232330000	7.373433000	10.707728000
H	-3.109303000	8.345991000	10.255602000
C	-4.284405000	7.099675000	11.561206000

H	-5.008525000	7.869319000	11.785951000
C	-4.387123000	5.827708000	12.112174000
H	-5.204311000	5.580835000	12.776532000
C	-3.429700000	4.868357000	11.807035000
H	-3.484597000	3.872067000	12.220968000
C	-2.403088000	5.224859000	10.955988000
C	-0.419911000	4.911497000	9.731391000
C	-0.889579000	3.178115000	11.125323000
H	-1.459124000	2.579331000	11.812626000
C	0.327439000	2.964383000	10.576138000
H	1.009840000	2.141696000	10.700342000
C	1.899859000	4.384584000	9.160457000
H	1.711946000	5.059619000	8.327424000
H	2.365900000	3.487588000	8.756584000
C	2.810056000	5.031838000	10.212585000
H	3.172121000	4.262447000	10.898122000
H	3.690125000	5.429894000	9.703584000
C	2.164352000	6.110202000	11.085651000
H	1.272214000	5.735149000	11.582161000
H	2.865184000	6.397499000	11.866807000
C	2.586322000	8.488690000	10.371308000
H	3.543993000	8.523700000	10.860269000
C	1.926263000	9.442463000	9.677178000
H	2.205464000	10.454176000	9.446481000
C	0.641019000	7.567439000	9.718045000
C	-0.324761000	9.381715000	8.506362000
C	-0.372523000	10.675369000	8.025792000
H	0.418097000	11.380351000	8.236451000
C	-1.471394000	11.041897000	7.257196000
H	-1.540790000	12.046441000	6.862017000
C	-2.470866000	10.111279000	7.000044000
H	-3.333674000	10.363637000	6.400711000
C	-2.343486000	8.838558000	7.526863000
H	-3.097243000	8.081092000	7.354227000

CCSD identifier	E	charge	Spin
$[^{N}(NCCN)Fe]$	-2325.89692812	+3	1/2

Fe	-0.874576000	6.926647000	9.624000000
N	-2.545679000	6.461266000	10.616407000
N	-1.454054000	4.443672000	10.707609000
N	0.543811000	4.215084000	9.918782000
N	2.068460000	7.465713000	10.310945000
N	0.957417000	8.920512000	9.141151000
N	-1.252231000	8.615642000	8.583706000
C	-3.567546000	7.291850000	10.909580000
H	-3.462443000	8.320596000	10.595370000
C	-4.690611000	6.857642000	11.588621000
H	-5.490073000	7.552627000	11.805325000
C	-4.763554000	5.526514000	11.983527000
H	-5.632172000	5.157640000	12.514302000
C	-3.704291000	4.662196000	11.704853000
H	-3.736941000	3.627685000	12.016432000
C	-2.620219000	5.173058000	11.031176000
C	-0.446482000	5.111894000	10.071411000
C	-1.094127000	3.139380000	10.949585000
H	-1.729326000	2.416659000	11.432550000
C	0.169511000	3.000045000	10.446157000
H	0.806972000	2.131387000	10.416188000
C	1.850711000	4.521001000	9.306387000
H	1.664850000	5.220560000	8.493964000
H	2.227997000	3.602912000	8.860291000
C	2.866449000	5.068533000	10.309795000
H	3.133545000	4.293846000	11.032002000
H	3.778683000	5.295463000	9.753945000
C	2.423622000	6.281738000	11.126822000
H	1.556951000	6.058755000	11.745424000
H	3.226709000	6.588744000	11.793713000
C	2.914706000	8.498591000	10.027460000
H	3.938882000	8.526467000	10.364736000
C	2.215007000	9.423908000	9.281844000
H	2.544210000	10.363932000	8.871359000
C	0.851986000	7.692088000	9.759719000
C	-0.181126000	9.433316000	8.467420000
C	-0.201428000	10.612778000	7.761031000
H	0.671257000	11.246563000	7.688727000
C	-1.392311000	10.967015000	7.124512000
H	-1.445144000	11.887832000	6.557524000
C	-2.496263000	10.128772000	7.215299000
H	-3.426978000	10.369329000	6.720049000
C	-2.390864000	8.961414000	7.953107000
H	-3.222451000	8.275345000	8.040971000

CCSD identifier	E	charge	Spin
$[^{H}(NCCN)Fe(NH_3)$	-2382.98046210	+2	0

Fe	-0.896872000	6.532252000	8.874985000
N	-2.190987000	5.627080000	7.491760000
H	-2.083200000	5.973353000	6.539938000
H	-3.172278000	5.754354000	7.730755000
H	-2.048635000	4.621412000	7.438707000
N	-2.286379000	6.427375000	10.285565000
N	-1.308935000	4.381561000	10.577599000
N	0.607724000	3.988007000	9.689395000
N	1.712841000	7.298897000	10.390199000
N	0.665045000	8.812723000	9.290362000
N	-1.296679000	8.434873000	8.161951000
C	-3.203179000	7.363140000	10.583208000
H	-3.109332000	8.303527000	10.061726000
C	-4.207995000	7.149243000	11.508445000
H	-4.919287000	7.934641000	11.719608000
C	-4.279255000	5.918541000	12.151690000
H	-5.057541000	5.719038000	12.875662000
C	-3.336809000	4.943602000	11.859595000
H	-3.361721000	3.978464000	12.344238000
C	-2.356918000	5.243210000	10.929938000
C	-0.415389000	4.856072000	9.656553000
C	-0.831442000	3.219198000	11.186490000
H	-1.378000000	2.668224000	11.929898000
C	0.372331000	2.977741000	10.626283000
H	1.068000000	2.174596000	10.795819000
C	1.915942000	4.343384000	9.137902000
H	1.732010000	5.012170000	8.298831000
H	2.402242000	3.450456000	8.748691000
C	2.795337000	5.012161000	10.203484000
H	3.175368000	4.248446000	10.885577000
H	3.667520000	5.445070000	9.709651000
C	2.100559000	6.059285000	11.077305000
H	1.209104000	5.652352000	11.549161000
H	2.776025000	6.349123000	11.879767000
C	2.495623000	8.448863000	10.420005000
H	3.439889000	8.487184000	10.933909000
C	1.840721000	9.405249000	9.729363000
H	2.111989000	10.424237000	9.524135000
C	0.573724000	7.505457000	9.696998000
C	-0.367210000	9.346349000	8.501809000
C	-0.427135000	10.675490000	8.119625000
H	0.338777000	11.375576000	8.418239000
C	-1.503295000	11.085078000	7.346170000
H	-1.580406000	12.116391000	7.029399000
C	-2.477669000	10.159439000	6.990505000
H	-3.331537000	10.442643000	6.392150000
C	-2.334808000	8.853112000	7.418150000
H	-3.078638000	8.110136000	7.167836000

CCSD identifier	E	charge	Spin
$[^{H}(NCCN)Fe(NH_3)$	-2382.51997024	+3	1/2

Fe	-0.979133000	6.664622000	9.054490000
N	-1.964894000	5.636935000	7.529970000
H	-1.837967000	6.076232000	6.617318000
H	-2.971955000	5.587375000	7.685526000
H	-1.656788000	4.671624000	7.422442000
N	-2.432194000	6.406015000	10.387653000
N	-1.357156000	4.413626000	10.675861000
N	0.561865000	4.086345000	9.760796000
N	1.806086000	7.327724000	10.356590000
N	0.749017000	8.827942000	9.219913000
N	-1.320403000	8.510595000	8.265535000
C	-3.396758000	7.308434000	10.659548000
H	-3.325226000	8.262378000	10.158396000
C	-4.420734000	7.030765000	11.543843000
H	-5.175794000	7.778899000	11.741813000
C	-4.455645000	5.785811000	12.166456000
H	-5.249904000	5.541974000	12.860481000
C	-3.457637000	4.853602000	11.902066000
H	-3.455714000	3.885993000	12.383917000
C	-2.463137000	5.207413000	11.014101000
C	-0.448621000	4.955278000	9.821344000
C	-0.907528000	3.186344000	11.155732000
H	-1.466826000	2.570251000	11.837815000
C	0.299186000	2.985548000	10.577008000
H	0.982743000	2.157459000	10.663581000
C	1.863921000	4.406344000	9.156257000
H	1.674888000	5.094138000	8.333888000
H	2.287217000	3.497275000	8.733122000
C	2.811803000	5.012250000	10.195591000
H	3.164608000	4.231147000	10.872375000
H	3.694532000	5.387475000	9.674444000
C	2.202546000	6.100650000	11.079512000
H	1.326490000	5.744834000	11.616929000
H	2.930600000	6.407296000	11.827246000
C	2.606010000	8.444788000	10.302881000
H	3.573071000	8.487178000	10.776177000
C	1.942780000	9.397146000	9.587898000
H	2.238261000	10.400317000	9.334573000
C	0.651801000	7.538907000	9.685746000
C	-0.313569000	9.382904000	8.468144000
C	-0.324042000	10.675685000	7.989343000
H	0.499125000	11.351388000	8.171144000
C	-1.434195000	11.091403000	7.258093000
H	-1.474445000	12.099637000	6.866036000
C	-2.482260000	10.204475000	7.039151000
H	-3.357957000	10.496579000	6.476178000
C	-2.388815000	8.926296000	7.557346000
H	-3.188014000	8.211481000	7.414766000

CCSD identifier	E	charge	Spin
$[^{Me}(NCCN)Fe$	-2404.99471485	+2	0

Fe	7.021411000	4.233357000	8.793762000
N	8.619561000	3.098600000	8.612436000
N	7.581095000	2.592018000	6.634344000
N	5.523980000	3.137282000	6.350166000
N	4.627135000	2.585215000	9.773814000
N	5.584732000	3.958487000	11.124701000
N	7.410049000	5.209870000	10.531588000
C	9.636515000	3.009806000	9.489409000
H	9.493457000	3.522876000	10.429130000
C	10.802824000	2.302419000	9.233482000
C	11.908566000	2.227692000	10.243894000
H	12.822281000	2.676151000	9.849272000
H	12.136118000	1.189498000	10.491303000
H	11.645644000	2.746082000	11.164743000
C	10.897426000	1.669517000	7.990780000
H	11.789665000	1.110924000	7.737244000
C	9.859180000	1.745477000	7.075373000
H	9.927670000	1.257977000	6.113631000
C	8.733950000	2.463565000	7.427053000
C	6.575454000	3.354289000	7.152796000
C	7.152935000	1.900642000	5.504531000
H	7.786708000	1.255327000	4.923929000
C	5.856898000	2.246244000	5.330787000
H	5.156787000	1.954303000	4.567692000
C	4.169314000	3.530223000	6.741813000
H	3.598176000	3.784523000	5.850622000
H	4.264280000	4.435339000	7.339045000
C	3.467539000	2.411924000	7.523575000
H	2.542466000	2.815052000	7.940480000
H	3.174453000	1.618740000	6.832177000
C	4.297516000	1.737338000	8.617903000
H	3.742552000	0.886123000	9.007053000
H	5.234107000	1.351683000	8.221321000
C	3.898421000	2.580835000	10.957651000
H	3.029262000	1.961473000	11.092690000
C	4.496294000	3.443823000	11.810013000
H	4.240523000	3.717319000	12.817210000
C	5.671735000	3.430253000	9.865240000
C	6.538813000	4.922375000	11.511390000
C	6.585369000	5.523673000	12.753948000
H	5.875434000	5.268670000	13.527078000
C	7.572382000	6.471584000	12.979906000
H	7.628948000	6.959737000	13.944708000
C	9.553056000	7.837941000	12.188939000

H	10.210712000	7.917934000	11.324873000
H	10.160973000	7.595647000	13.062124000
H	9.106098000	8.818212000	12.365901000
C	8.487616000	6.804535000	11.977420000
C	8.357650000	6.133836000	10.767754000
H	9.036376000	6.338823000	9.948906000

CCSD identifier	E	charge	Spin
$[^{Me}(NCCN)Fe$	-2404.52946436	+3	1/2

Fe	7.081416000	3.720285000	9.135536000
N	8.862292000	2.945192000	8.670065000
N	7.710825000	2.496161000	6.734093000
N	5.609378000	2.957103000	6.543190000
N	4.301416000	2.656697000	9.890501000
N	5.277854000	4.114371000	11.171882000
N	7.362057000	4.964611000	10.702058000
C	9.948222000	2.884581000	9.469454000
H	9.831998000	3.288654000	10.465555000
C	11.156360000	2.328468000	9.064969000
C	12.340815000	2.276999000	9.979322000
H	13.172569000	2.846316000	9.558788000
H	12.683347000	1.247577000	10.101345000
H	12.111167000	2.679445000	10.964248000
C	11.207766000	1.818023000	7.762560000
H	12.125583000	1.375247000	7.394601000
C	10.092844000	1.855883000	6.932007000
H	10.134591000	1.446398000	5.932457000
C	8.939800000	2.419029000	7.427341000
C	6.647612000	3.040513000	7.394616000
C	7.339525000	2.076337000	5.479213000
H	8.011718000	1.623596000	4.770780000
C	6.009878000	2.372604000	5.363094000
H	5.340927000	2.223336000	4.531421000
C	4.238691000	3.395753000	6.865062000
H	3.759950000	3.693111000	5.934250000
H	4.328747000	4.287606000	7.481625000
C	3.411540000	2.309247000	7.553583000
H	2.436250000	2.742720000	7.784789000
H	3.224705000	1.486241000	6.860342000
C	4.027723000	1.687325000	8.805703000
H	3.352428000	0.938788000	9.215062000
H	4.970122000	1.189459000	8.588160000
C	3.465904000	2.907863000	10.940910000
H	2.508576000	2.424601000	11.055147000

C	4.079959000	3.835257000	11.755447000
H	3.732837000	4.279835000	12.672981000
C	5.426201000	3.403794000	9.998174000
C	6.319236000	4.994287000	11.558160000
C	6.285222000	5.809662000	12.666596000
H	5.440357000	5.815501000	13.340992000
C	7.375144000	6.643331000	12.892628000
H	7.370922000	7.296640000	13.756830000
C	9.635439000	7.564132000	12.226640000
H	10.379125000	7.461261000	11.438623000
H	10.114310000	7.351974000	13.185416000
H	9.308849000	8.605961000	12.256020000
C	8.465043000	6.657398000	12.015445000
C	8.403800000	5.786776000	10.928277000
H	9.207816000	5.751846000	10.204919000

CCSD identifier	E	charge	Spin
$[^{Me}(NCCN)Fe(NH_3)$	-2461.60331326	+2	0

Fe	7.012246000	4.397054000	8.658310000
N	8.037384000	5.865821000	7.566122000
H	7.838016000	5.811563000	6.570343000
H	9.047636000	5.776266000	7.651790000
H	7.810706000	6.818709000	7.844952000
N	8.589220000	3.199223000	8.562425000
N	7.546863000	2.598362000	6.617619000
N	5.498468000	3.159654000	6.292314000
N	4.711650000	2.584097000	9.717320000
N	5.664184000	3.953506000	11.064608000
N	7.408187000	5.321570000	10.469058000
C	9.593318000	3.121286000	9.452788000
H	9.470055000	3.705300000	10.352960000
C	10.725187000	2.340648000	9.262235000
C	11.810877000	2.276095000	10.295339000
H	12.770102000	2.575924000	9.869793000
H	11.926348000	1.257219000	10.669748000
H	11.596577000	2.925008000	11.143352000
C	10.803122000	1.617068000	8.068391000
H	11.668554000	0.998698000	7.866720000
C	9.778167000	1.681057000	7.140369000
H	9.827413000	1.122952000	6.216544000
C	8.684658000	2.477780000	7.429914000
C	6.554553000	3.416309000	7.081646000
C	7.100696000	1.828011000	5.542940000
H	7.721541000	1.127299000	5.015279000

C	5.813739000	2.183290000	5.343580000
H	5.106247000	1.845549000	4.606762000
C	4.144076000	3.536758000	6.698479000
H	3.551290000	3.770941000	5.815763000
H	4.234437000	4.448155000	7.286909000
C	3.481549000	2.411730000	7.506258000
H	2.570261000	2.803149000	7.962652000
H	3.168558000	1.617883000	6.824503000
C	4.365029000	1.744486000	8.563154000
H	3.843272000	0.874877000	8.957793000
H	5.294475000	1.384062000	8.128580000
C	4.010635000	2.537810000	10.917764000
H	3.160575000	1.894557000	11.061628000
C	4.606404000	3.398642000	11.769871000
H	4.367069000	3.649120000	12.786731000
C	5.741311000	3.454136000	9.790345000
C	6.593847000	4.930456000	11.461026000
C	6.674557000	5.438637000	12.746359000
H	6.006189000	5.098085000	13.523276000
C	7.640237000	6.393964000	13.011901000
H	7.722105000	6.807760000	14.009049000
C	9.566383000	7.855893000	12.258339000
H	10.176278000	8.032103000	11.373338000
H	10.225970000	7.546279000	13.070757000
H	9.114086000	8.805053000	12.552054000
C	8.509813000	6.822300000	12.004615000
C	8.343098000	6.245135000	10.753799000
H	8.996632000	6.530304000	9.940644000

CCSD identifier	E	charge	Spin
$[^{Me}(NCCN)Fe(NH_3)$	-2461.15096786	+3	1/2

Fe	7.116329000	4.237734000	8.807479000
N	7.811367000	5.790283000	7.600381000
H	7.427628000	5.777812000	6.656766000
H	8.824535000	5.761828000	7.486625000
H	7.591464000	6.709873000	7.984157000
N	8.736439000	3.114137000	8.561207000
N	7.609384000	2.498366000	6.673492000
N	5.561971000	3.095930000	6.395993000
N	4.587351000	2.618759000	9.747047000
N	5.538382000	4.029175000	11.075933000
N	7.426539000	5.236006000	10.553331000
C	9.777132000	3.068931000	9.419354000
H	9.674309000	3.644056000	10.328217000
C	10.921184000	2.321607000	9.172414000
C	12.056064000	2.281080000	10.149221000
H	12.980588000	2.618745000	9.676859000
H	12.224276000	1.258714000	10.493941000
H	11.865635000	2.906764000	11.019383000
C	10.960322000	1.604718000	7.968689000

H	11.833145000	1.010534000	7.726351000
C	9.893020000	1.634425000	7.083269000
H	9.918761000	1.069903000	6.161724000
C	8.793625000	2.396792000	7.420289000
C	6.614638000	3.258205000	7.201213000
C	7.173968000	1.851439000	5.521111000
H	7.796407000	1.204253000	4.928605000
C	5.885579000	2.230761000	5.351122000
H	5.180914000	1.973746000	4.578115000
C	4.203147000	3.531330000	6.750764000
H	3.674142000	3.807661000	5.840617000
H	4.302659000	4.429095000	7.358332000
C	3.454224000	2.422444000	7.495533000
H	2.522162000	2.839285000	7.881650000
H	3.170490000	1.634843000	6.794297000
C	4.243237000	1.738220000	8.611797000
H	3.652227000	0.922897000	9.023143000
H	5.171325000	1.304229000	8.246651000
C	3.844149000	2.662581000	10.902584000
H	2.960949000	2.062354000	11.045288000
C	4.441152000	3.550809000	11.747152000
H	4.162360000	3.853288000	12.741294000
C	5.640283000	3.463261000	9.829277000
C	6.503260000	4.976404000	11.494134000
C	6.508625000	5.583101000	12.733100000
H	5.756531000	5.356772000	13.475118000
C	7.513163000	6.501936000	13.005548000
H	7.535362000	6.992319000	13.971195000
C	9.582410000	7.790048000	12.317626000
H	10.270900000	7.873362000	11.478463000
H	10.152090000	7.504387000	13.204022000
H	9.159374000	8.777914000	12.512330000
C	8.491845000	6.799551000	12.049990000
C	8.396465000	6.129166000	10.836137000
H	9.130364000	6.305757000	10.060479000

CCSD identifier	E	charge	Spin
$[^{NMe2}(NCCN)Fe(NCCH_3)(NH_2\cdot)]$	-2781.91298023	+2	1/2

Fe	4.400674000	10.002167000	1.988217000
N	5.221560000	9.177785000	0.419315000
N	5.652784000	9.121279000	3.013981000
H	5.525980000	8.875087000	3.996168000
H	6.306941000	8.479321000	2.564448000
N	3.175608000	10.991995000	0.651562000
N	1.300741000	13.069079000	-2.483160000
N	4.940208000	12.436995000	0.693106000
N	6.413521000	12.275758000	2.268613000
N	3.778395000	11.570918000	4.543640000
N	2.696883000	9.739556000	4.160440000
N	3.011335000	8.537928000	2.245517000

N	0.225308000	5.615483000	3.383169000
C	5.764191000	8.713956000	-0.497310000
C	6.445209000	8.136296000	-1.638022000
H	5.818582000	8.222398000	-2.536910000
H	6.657356000	7.073509000	-1.453850000
H	7.395271000	8.660035000	-1.816063000
C	1.975096000	10.607712000	0.159551000
H	1.527369000	9.731466000	0.624793000
C	1.319261000	11.260341000	-0.861086000
H	0.350276000	10.884249000	-1.178486000
C	1.904065000	12.405371000	-1.473647000
C	-0.006234000	12.633866000	-2.972364000
H	0.034365000	11.600152000	-3.347534000
H	-0.311837000	13.282559000	-3.797735000
H	-0.770890000	12.698131000	-2.183085000
C	1.929468000	14.248758000	-3.070383000
H	2.038130000	15.056754000	-2.329580000
H	1.302983000	14.618276000	-3.886590000
H	2.920031000	14.006414000	-3.485147000
C	3.163714000	12.812590000	-0.954255000
H	3.683922000	13.681304000	-1.347783000
C	3.724512000	12.089115000	0.076898000
C	5.369029000	11.622535000	1.708693000
C	5.715465000	13.590499000	0.616727000
H	5.563225000	14.367400000	-0.121662000
C	6.637184000	13.486047000	1.610894000
H	7.436099000	14.160047000	1.898600000
C	6.897754000	11.945617000	3.608015000
H	7.959851000	12.211035000	3.682165000
H	6.796887000	10.856413000	3.701386000
C	6.079322000	12.687714000	4.681137000
H	6.267182000	12.213312000	5.655870000
H	6.444823000	13.722925000	4.767734000
C	4.573467000	12.802684000	4.421844000
H	4.150461000	13.507035000	5.149020000
H	4.377156000	13.227348000	3.428885000
C	2.973785000	11.314986000	5.654993000
H	2.947329000	11.980795000	6.509703000
C	2.292785000	10.165399000	5.419451000
H	1.571124000	9.638612000	6.030170000
C	3.616436000	10.601328000	3.602970000
C	2.334662000	8.604138000	3.416318000
C	1.405596000	7.679521000	3.845065000
H	0.907752000	7.814050000	4.801027000
C	-0.471144000	5.714974000	4.663436000
H	0.238688000	5.707288000	5.505198000
H	-1.137762000	4.855732000	4.774637000
H	-1.081853000	6.629763000	4.715996000
C	-0.038945000	4.470812000	2.511936000
H	-0.422184000	4.795906000	1.532988000
H	-0.796366000	3.836543000	2.980027000
H	0.868696000	3.867212000	2.359749000
C	1.118677000	6.558372000	3.017900000
C	1.831033000	6.490206000	1.784264000
H	1.679050000	5.667208000	1.091067000
C	2.740020000	7.471114000	1.458292000
H	3.294054000	7.422348000	0.521751000

CCSD identifier	E	charge	Spin
$[^{NMe2}(NCCN)Fe(NCCH_3)(NH_2\cdot)]$	-2781.49562932	+3	1

Fe	4.316484000	10.039810000	1.976956000
N	5.178912000	9.191923000	0.385091000
N	5.572339000	9.143316000	2.979620000
H	5.441556000	8.826672000	3.940548000
H	6.333179000	8.642061000	2.519537000
N	3.161653000	11.027471000	0.646886000
N	1.340748000	13.110941000	-2.510854000
N	4.942324000	12.459866000	0.698583000
N	6.374877000	12.284210000	2.309487000
N	3.724588000	11.617695000	4.555695000
N	2.651563000	9.773318000	4.182957000
N	2.932355000	8.582693000	2.246772000
N	0.277032000	5.578788000	3.471820000
C	5.763228000	8.713179000	-0.496493000
C	6.495595000	8.110386000	-1.587780000
H	5.895654000	8.141783000	-2.508864000
H	6.729391000	7.062179000	-1.349663000
H	7.436307000	8.655394000	-1.755419000
C	1.962970000	10.642913000	0.135512000
H	1.504837000	9.769776000	0.594542000
C	1.325825000	11.298362000	-0.891126000
H	0.364675000	10.918484000	-1.228010000
C	1.928888000	12.439622000	-1.501600000
C	-0.011101000	12.779095000	-2.970260000
H	0.010214000	11.934194000	-3.676715000
H	-0.428655000	13.650708000	-3.483760000
H	-0.663261000	12.535905000	-2.123604000
C	2.030117000	14.206133000	-3.197555000
H	2.013545000	15.124190000	-2.588634000
H	1.515968000	14.408893000	-4.141387000
H	3.068169000	13.932228000	-3.425503000
C	3.194702000	12.834324000	-0.976642000
H	3.721284000	13.702532000	-1.363258000
C	3.735852000	12.121527000	0.066767000
C	5.332847000	11.653738000	1.732574000
C	5.750286000	13.591502000	0.619988000
H	5.637654000	14.363217000	-0.131731000
C	6.647640000	13.475962000	1.635398000
H	7.459083000	14.133084000	1.930082000
C	6.844934000	11.962211000	3.659690000
H	7.909542000	12.214788000	3.737269000
H	6.735882000	10.876839000	3.771959000
C	6.026254000	12.730275000	4.712963000
H	6.207526000	12.274285000	5.697448000
H	6.399073000	13.763754000	4.784318000
C	4.524777000	12.854713000	4.440878000
H	4.095651000	13.555610000	5.167297000
H	4.329791000	13.279286000	3.448062000
C	2.942749000	11.350547000	5.673576000
H	2.918945000	12.013779000	6.531615000
C	2.265566000	10.191716000	5.444749000
H	1.553301000	9.664001000	6.067034000
C	3.550972000	10.648921000	3.621864000
C	2.291500000	8.629170000	3.439997000
C	1.404469000	7.679984000	3.896253000
H	0.933304000	7.795731000	4.868493000
C	-0.376531000	5.644809000	4.781679000
H	0.369055000	5.674064000	5.590148000
H	-0.992494000	4.752771000	4.918018000
H	-1.029351000	6.528067000	4.850996000
C	0.007884000	4.425774000	2.606856000
H	-0.393764000	4.750976000	1.636207000
H	-0.733538000	3.784664000	3.089461000
H	0.923496000	3.837719000	2.442875000
C	1.124610000	6.547736000	3.074201000
C	1.797085000	6.501467000	1.815592000

H	1.649332000	5.673171000	1.127532000
C	2.665547000	7.506582000	1.461932000
H	3.192774000	7.471675000	0.509772000

CCSD identifier	E	charge	Spin
$[^{H}(NCCN)Fe(NCCH_3)(NH_2\cdot)]$	-2438.03262900	+2	1/2

Fe	-0.782072000	6.498916000	8.748168000
N	-2.234121000	5.575897000	7.538389000
H	-2.225471000	5.962168000	6.588651000
H	-3.196375000	5.657366000	7.882740000
H	-2.056891000	4.571438000	7.438003000
N	0.358740000	6.310640000	7.319881000
H	0.175083000	5.694943000	6.525484000
H	1.182464000	6.884177000	7.137273000
N	-2.206407000	6.478981000	10.227986000
N	-1.274140000	4.418508000	10.556050000
N	0.639599000	3.957835000	9.654474000
N	1.704638000	7.265656000	10.363483000
N	0.636584000	8.817219000	9.303107000
N	-1.304181000	8.410150000	8.159798000
C	-3.117381000	7.435508000	10.514962000
H	-3.008027000	8.377701000	9.981971000
C	-4.133977000	7.249280000	11.443911000
H	-4.834643000	8.058867000	11.643687000
C	-4.231314000	6.024904000	12.108188000
H	-5.021287000	5.847423000	12.838025000
C	-3.299390000	5.027351000	11.831373000
H	-3.341514000	4.060711000	12.331403000
C	-2.307100000	5.301563000	10.896197000
C	-0.366003000	4.865430000	9.620746000
C	-0.835487000	3.240086000	11.161888000
H	-1.401379000	2.705904000	11.915206000
C	0.365855000	2.957806000	10.592538000
H	1.043817000	2.128997000	10.764413000
C	1.974184000	4.256408000	9.129561000
H	1.819065000	4.887330000	8.246554000
H	2.453799000	3.320973000	8.815526000
C	2.827265000	4.973436000	10.192131000
H	3.224370000	4.232707000	10.903396000
H	3.701243000	5.418331000	9.693493000
C	2.103471000	6.021791000	11.042746000
H	1.207377000	5.599968000	11.516234000
H	2.769479000	6.324470000	11.860438000
C	2.432148000	8.451708000	10.502372000
H	3.352675000	8.496201000	11.073309000
C	1.767090000	9.429930000	9.839126000
H	1.998493000	10.480620000	9.717415000
C	0.583009000	7.473441000	9.623024000
C	-0.398956000	9.347080000	8.525873000
C	-0.495948000	10.689750000	8.166267000
H	0.256691000	11.409653000	8.483947000
C	-1.582843000	11.082949000	7.391194000
H	-1.689365000	12.125057000	7.089382000
C	-2.531274000	10.129920000	7.009054000
H	-3.395216000	10.400482000	6.403668000
C	-2.353305000	8.813480000	7.412608000
H	-3.077548000	8.047425000	7.136358000

CCSD identifier	E	charge	Spin
$[^{H}(NCCN)Fe(NCCH_3)(NH_2\cdot)]$	-2437.56651326	+3	1

Fe	-0.802320000	6.498646000	8.742809000
N	-2.302911000	5.565565000	7.588011000
H	-2.300157000	5.896274000	6.615939000
H	-3.253079000	5.712824000	7.946725000
H	-2.181448000	4.547085000	7.539099000
N	0.290728000	6.307275000	7.282869000
H	0.160622000	5.584455000	6.570367000
H	1.094994000	6.895968000	7.060191000
N	-2.197534000	6.474748000	10.240090000
N	-1.256904000	4.412597000	10.557515000
N	0.667611000	3.976102000	9.652855000
N	1.689061000	7.271129000	10.407724000
N	0.621968000	8.822660000	9.324068000
N	-1.310525000	8.429287000	8.149872000
C	-3.104389000	7.437921000	10.529296000
H	-2.997031000	8.381807000	9.999309000
C	-4.116499000	7.245893000	11.461741000
H	-4.815208000	8.056037000	11.670475000
C	-4.216358000	6.015480000	12.118390000
H	-5.007452000	5.836801000	12.848188000
C	-3.287581000	5.013866000	11.836799000
H	-3.335082000	4.045650000	12.334779000
C	-2.296812000	5.290844000	10.903586000
C	-0.344455000	4.870781000	9.635867000
C	-0.827676000	3.219471000	11.123974000
H	-1.394066000	2.661839000	11.862052000
C	0.383540000	2.950278000	10.551127000
H	1.057996000	2.113107000	10.705848000
C	2.007092000	4.276257000	9.127695000
H	1.863626000	4.899709000	8.238206000
H	2.486717000	3.339882000	8.817355000
C	2.850252000	4.994512000	10.197855000
H	3.258825000	4.250859000	10.899554000
H	3.720383000	5.451852000	9.704002000
C	2.119602000	6.018170000	11.071522000
H	1.237247000	5.586491000	11.560883000
H	2.794455000	6.329592000	11.878245000
C	2.393248000	8.453736000	10.561914000
H	3.305710000	8.513097000	11.147728000
C	1.721203000	9.437660000	9.887600000
H	1.941966000	10.495397000	9.793730000
C	0.583388000	7.480701000	9.641495000
C	-0.405863000	9.362335000	8.519731000
C	-0.477348000	10.696689000	8.139722000
H	0.271255000	11.419467000	8.462750000
C	-1.541117000	11.086441000	7.323364000
H	-1.629386000	12.125075000	7.001363000
C	-2.483216000	10.134939000	6.928288000
H	-3.328110000	10.401953000	6.293449000
C	-2.333794000	8.820870000	7.360763000
H	-3.060489000	8.060545000	7.077042000

CCSD identifier	E	charge	Spin
$[^{Me}(NCCN)Fe(NCCH_3)(NH_2\cdot)]$	-2516.59599537	+2	1/2

Fe	6.875289000	4.499335000	8.622732000
N	8.067786000	5.828693000	7.516371000
H	7.803188000	5.840178000	6.526411000
H	9.071134000	5.619701000	7.537668000
H	7.956232000	6.790248000	7.853719000
N	5.522142000	5.724373000	8.411402000
H	5.543474000	6.475363000	7.719130000
H	4.717227000	5.837516000	9.027956000
N	8.508710000	3.249455000	8.605951000
N	7.511277000	2.623563000	6.646285000
N	5.457648000	3.190209000	6.260714000
N	4.717655000	2.599992000	9.682258000
N	5.697057000	3.936969000	11.069362000
N	7.416006000	5.313973000	10.444757000
C	9.508041000	3.179349000	9.513544000
H	9.369821000	3.767474000	10.419443000
C	10.655277000	2.400895000	9.345544000
C	11.717729000	2.348189000	10.402331000
H	12.695544000	2.635871000	9.989994000
H	11.823327000	1.326942000	10.796929000
H	11.489462000	3.014954000	11.242994000
C	10.760507000	1.667279000	8.152263000
H	11.640208000	1.048666000	7.967182000
C	9.744540000	1.719238000	7.204962000
H	9.811699000	1.152580000	6.277107000
C	8.636267000	2.514029000	7.475894000
C	6.499799000	3.445703000	7.089828000
C	7.101591000	1.866995000	5.548092000
H	7.744682000	1.165271000	5.031332000
C	5.811811000	2.226267000	5.312442000
H	5.119887000	1.889240000	4.548574000
C	4.078087000	3.525534000	6.620187000
H	3.496978000	3.684900000	5.703213000
H	4.127785000	4.470675000	7.173553000
C	3.450007000	2.405333000	7.470564000
H	2.534885000	2.796121000	7.939771000
H	3.130510000	1.582505000	6.812346000
C	4.362113000	1.766854000	8.522687000
H	3.857370000	0.880979000	8.927901000
H	5.297171000	1.411412000	8.071116000
C	4.089837000	2.456166000	10.922587000
H	3.272883000	1.760047000	11.074754000
C	4.700332000	3.293456000	11.798384000
H	4.514064000	3.469726000	12.850308000
C	5.721206000	3.515487000	9.754421000
C	6.631911000	4.901680000	11.463114000
C	6.756642000	5.391807000	12.761386000

H	6.106547000	5.035348000	13.559095000
C	7.733522000	6.346764000	13.010563000
H	7.849296000	6.749192000	14.018357000
C	9.635118000	7.827804000	12.207417000
H	10.229653000	8.010649000	11.303758000
H	10.317471000	7.516649000	13.011301000
H	9.186852000	8.782896000	12.518619000
C	8.573175000	6.795308000	11.975605000
C	8.363690000	6.236788000	10.714543000
H	8.994573000	6.542253000	9.878576000

CCSD identifier	E	charge	Spin
$[^{Me}(NCCN)Fe(NCCH_3)(NH_2\cdot)]$	-2516.13261927	+3	1

Fe	6.942266000	4.516706000	8.621736000
N	8.035852000	5.647192000	7.616513000
H	9.019005000	5.442846000	7.422540000
H	7.719993000	6.401682000	7.001800000
N	5.562011000	6.030348000	8.441417000
H	5.298414000	6.225870000	7.469351000
H	5.987193000	6.890042000	8.808869000
H	4.691789000	5.893680000	8.965306000
N	8.466148000	3.242925000	8.593008000
N	7.436329000	2.642318000	6.631971000
N	5.376877000	3.210938000	6.289085000
N	4.736168000	2.535672000	9.695337000
N	5.724221000	3.878932000	11.087612000
N	7.377502000	5.342505000	10.464455000
C	9.465933000	3.167156000	9.502527000
H	9.339313000	3.758121000	10.407486000
C	10.606640000	2.381737000	9.316327000
C	11.680164000	2.308721000	10.355409000
H	12.650534000	2.606565000	9.930767000
H	11.796805000	1.275322000	10.715857000
H	11.466976000	2.951298000	11.217798000
C	10.698257000	1.667485000	8.105531000
H	11.578010000	1.052903000	7.903662000
C	9.678654000	1.732545000	7.160615000
H	9.745717000	1.178510000	6.224521000
C	8.573724000	2.523203000	7.443949000
C	6.433440000	3.451303000	7.092042000
C	7.019167000	1.918415000	5.516378000
H	7.658677000	1.235087000	4.968968000
C	5.722609000	2.275771000	5.307412000
H	5.020888000	1.954501000	4.543983000
C	3.991124000	3.504559000	6.677715000
H	3.392292000	3.668749000	5.773053000
H	3.993098000	4.441849000	7.241772000
C	3.406837000	2.350308000	7.512479000
H	2.485777000	2.702052000	8.000534000
H	3.098925000	1.535304000	6.839821000

C	4.355067000	1.706997000	8.527274000
H	3.870211000	0.814317000	8.940883000
H	5.283440000	1.364621000	8.053710000
C	4.164222000	2.346958000	10.938659000
H	3.374299000	1.622052000	11.111996000
C	4.785247000	3.192329000	11.822598000
H	4.624032000	3.329901000	12.886342000
C	5.713635000	3.485769000	9.760789000
C	6.636043000	4.878661000	11.492024000
C	6.762704000	5.351251000	12.793604000
H	6.152787000	4.953320000	13.604261000
C	7.695678000	6.355584000	13.034080000
H	7.814671000	6.746187000	14.046678000
C	9.480775000	7.959442000	12.209810000
H	10.027984000	8.215654000	11.295209000
H	10.207198000	7.664343000	12.982047000
H	8.983442000	8.868870000	12.581599000
C	8.479755000	6.872400000	11.985851000
C	8.275920000	6.321046000	10.712451000
H	8.846095000	6.668295000	9.849754000

CCSD identifier	E	charge	Spin
$[^{NMe2}(NCCN)Fe(NCCH_3)(NH^{**})]$	-2780.82575032	+2	1

Fe	4.256499000	10.037909000	1.928809000
N	5.010131000	9.145866000	0.310580000
N	5.488970000	9.217594000	2.872160000
H	6.406200000	8.845181000	2.582968000
N	3.107976000	11.089012000	0.563451000
N	1.444126000	13.251901000	-2.624335000
N	4.951414000	12.441738000	0.663469000
N	6.331054000	12.232812000	2.324190000
N	3.659186000	11.588953000	4.542309000
N	2.651778000	9.708653000	4.190836000
N	2.863072000	8.553925000	2.223144000
N	0.383933000	5.444989000	3.545931000
C	5.565043000	8.629239000	-0.568200000
C	6.265624000	7.975942000	-1.650856000
H	5.636568000	7.947920000	-2.552122000
H	6.524796000	6.946933000	-1.360330000
H	7.193206000	8.521896000	-1.878951000
C	1.902269000	10.762958000	0.035791000
H	1.396213000	9.912517000	0.490216000
C	1.311030000	11.448585000	-1.000588000
H	0.336087000	11.122234000	-1.353176000
C	1.982315000	12.555619000	-1.598495000
C	0.088139000	12.958228000	-3.094579000
H	0.056032000	11.990884000	-3.620417000
H	-0.223086000	13.742164000	-3.789940000
H	-0.619148000	12.938059000	-2.254166000
C	2.181125000	14.332532000	-3.281205000

H	2.179018000	15.244733000	-2.662886000
H	1.697978000	14.561713000	-4.235175000
H	3.216261000	14.030483000	-3.487404000
C	3.254424000	12.892607000	-1.051339000
H	3.829463000	13.731823000	-1.433284000
C	3.740200000	12.149987000	0.003680000
C	5.296757000	11.619337000	1.707750000
C	5.783785000	13.550313000	0.617284000
H	5.709956000	14.331672000	-0.129667000
C	6.647517000	13.414545000	1.665131000
H	7.456714000	14.060766000	1.989423000
C	6.739092000	11.912026000	3.699487000
H	7.812833000	12.107777000	3.808718000
H	6.560331000	10.837774000	3.833599000
C	5.923180000	12.741873000	4.703634000
H	6.095529000	12.328480000	5.708383000
H	6.300237000	13.775890000	4.729328000
C	4.423864000	12.843238000	4.410717000
H	3.971078000	13.553151000	5.113998000
H	4.233083000	13.237072000	3.404636000
C	2.969565000	11.257611000	5.707041000
H	2.969272000	11.903200000	6.578544000
C	2.337968000	10.074271000	5.492593000
H	1.695875000	9.496417000	6.145392000
C	3.469248000	10.637960000	3.602369000
C	2.304875000	8.557752000	3.460289000
C	1.478391000	7.570472000	3.949698000
H	1.056990000	7.657406000	4.947174000
C	-0.059764000	5.368791000	4.938100000
H	0.762481000	5.613894000	5.622392000
H	-0.389394000	4.347014000	5.148460000
H	-0.907081000	6.050833000	5.118042000
C	-0.044057000	4.376601000	2.640111000
H	-0.365844000	4.787749000	1.675384000
H	-0.890443000	3.851360000	3.092265000
H	0.770179000	3.651775000	2.477742000
C	1.165978000	6.460776000	3.113471000
C	1.717022000	6.480361000	1.798871000
H	1.536473000	5.670196000	1.096976000
C	2.539138000	7.517711000	1.415162000
H	2.989409000	7.526780000	0.423926000

CCSD identifier	E	charge	Spin
$[^{NMe2}(NCCN)Fe(NCCH_3)(NH^{**})]$	-2781.25459115	+3	3/2

Fe	4.366544000	10.020063000	1.981520000
N	5.097364000	9.192617000	0.349003000
N	5.618883000	9.205259000	2.879551000
H	6.555525000	8.920867000	2.558093000
N	3.159047000	11.052032000	0.626866000
N	1.426143000	13.158358000	-2.560884000
N	4.956662000	12.456038000	0.723736000
N	6.374068000	12.267157000	2.349948000
N	3.687229000	11.618995000	4.546675000
N	2.659382000	9.757981000	4.176545000
N	2.959844000	8.537201000	2.262615000
N	0.316180000	5.539525000	3.534865000
C	5.588727000	8.705854000	-0.582345000
C	6.213724000	8.088669000	-1.733785000
H	5.512594000	8.065013000	-2.579696000
H	6.514287000	7.059564000	-1.490336000
H	7.107187000	8.657623000	-2.027896000
C	1.974469000	10.678791000	0.090543000
H	1.504234000	9.804966000	0.539983000

C	1.362959000	11.342848000	-0.949066000
H	0.400467000	10.983126000	-1.303064000
C	1.986793000	12.482239000	-1.536488000
C	0.132141000	12.740379000	-3.099416000
H	0.178262000	11.714126000	-3.493861000
H	-0.144263000	13.408340000	-3.919588000
H	-0.654980000	12.794267000	-2.332197000
C	2.095417000	14.324543000	-3.131655000
H	2.214435000	15.123412000	-2.383103000
H	1.489543000	14.716032000	-3.952976000
H	3.084882000	14.058549000	-3.534428000
C	3.238569000	12.867300000	-0.979286000
H	3.788967000	13.721955000	-1.362256000
C	3.750430000	12.135924000	0.071071000
C	5.349217000	11.623955000	1.743024000
C	5.739610000	13.606691000	0.691861000
H	5.617768000	14.393773000	-0.041496000
C	6.624251000	13.485595000	1.717008000
H	7.414338000	14.153325000	2.041447000
C	6.788629000	11.937138000	3.715786000
H	7.860470000	12.142479000	3.829782000
H	6.614756000	10.858804000	3.833157000
C	5.966480000	12.743798000	4.734441000
H	6.121673000	12.307177000	5.732189000
H	6.346111000	13.776296000	4.784874000
C	4.471274000	12.857840000	4.425362000
H	4.019555000	13.573184000	5.123925000
H	4.304225000	13.257880000	3.417319000
C	2.912614000	11.338519000	5.670059000
H	2.877575000	12.003689000	6.524927000
C	2.264698000	10.167141000	5.441009000
H	1.567222000	9.618728000	6.060487000
C	3.544784000	10.642243000	3.618230000
C	2.319951000	8.596983000	3.450825000
C	1.438241000	7.650134000	3.924518000
H	0.969813000	7.779191000	4.895778000
C	-0.329600000	5.629973000	4.842341000
H	0.414761000	5.659335000	5.653092000
H	-0.958338000	4.748174000	4.991243000
H	-0.971585000	6.522052000	4.909472000
C	0.061817000	4.372967000	2.690186000
H	-0.348677000	4.672143000	1.714258000
H	-0.671390000	3.730474000	3.184885000
H	0.980393000	3.788121000	2.529604000
C	1.162636000	6.506202000	3.123894000
C	1.834638000	6.444386000	1.868725000
H	1.688857000	5.606301000	1.192512000
C	2.699769000	7.450531000	1.501026000
H	3.227112000	7.402756000	0.549301000

CCSD identifier	E	charge	Spin
$[^{H}(NCCN)Fe(NCCH_3)(NH^{**})]$	-2437.37964088	+2	1

Fe	-0.763321000	6.479181000	8.722938000
N	-2.255149000	5.550437000	7.524589000
H	-2.056698000	5.812287000	6.553986000
H	-3.241233000	5.751497000	7.717151000
H	-2.152293000	4.532002000	7.561322000
N	0.370100000	6.303495000	7.430292000
H	1.165679000	6.838393000	7.057425000
N	-2.232577000	6.494704000	10.233048000
N	-1.266788000	4.452460000	10.595172000
N	0.626675000	3.957853000	9.669313000
N	1.714692000	7.261810000	10.372093000

N	0.593514000	8.822278000	9.383999000
N	-1.273213000	8.398025000	8.117559000
C	-3.154944000	7.442529000	10.499449000
H	-3.070779000	8.367970000	9.931719000
C	-4.158476000	7.266754000	11.444067000
H	-4.874879000	8.066163000	11.627924000
C	-4.224622000	6.058379000	12.141279000
H	-5.004184000	5.886780000	12.883833000
C	-3.281040000	5.068030000	11.881457000
H	-3.305029000	4.114440000	12.406818000
C	-2.303499000	5.335135000	10.927781000
C	-0.367622000	4.872307000	9.641675000
C	-0.830905000	3.277735000	11.207794000
H	-1.391099000	2.757287000	11.974688000
C	0.358522000	2.975372000	10.624084000
H	1.029370000	2.140780000	10.795443000
C	1.940705000	4.224252000	9.082866000
H	1.751931000	4.818263000	8.179672000
H	2.399832000	3.272912000	8.787145000
C	2.843354000	4.973087000	10.083319000
H	3.308139000	4.246038000	10.767180000
H	3.669968000	5.439186000	9.527174000
C	2.159454000	5.999930000	10.992542000
H	1.295950000	5.561149000	11.508926000
H	2.870497000	6.291178000	11.775237000
C	2.402734000	8.461586000	10.561842000
H	3.321382000	8.511719000	11.135145000
C	1.704139000	9.445817000	9.941840000
H	1.902103000	10.507488000	9.865271000
C	0.593651000	7.472818000	9.643128000
C	-0.425319000	9.346431000	8.572399000
C	-0.544541000	10.697076000	8.259714000
H	0.159037000	11.428485000	8.654009000
C	-1.590437000	11.084877000	7.425941000
H	-1.712811000	12.134121000	7.156532000
C	-2.472670000	10.117455000	6.941461000
H	-3.299074000	10.381178000	6.282960000
C	-2.278522000	8.792180000	7.311416000
H	-2.948763000	8.012448000	6.952360000

CCSD identifier	E	charge	Spin
$[^{H}(NCCN)Fe(NCCH_3)(NH^{**})]$	-2436.89843942	+3	3/2

Fe	-0.817297000	6.469975000	8.725798000
N	-2.337169000	5.655955000	7.539600000

H	-2.280594000	6.025391000	6.583038000
H	-3.290580000	5.822554000	7.881417000
H	-2.235387000	4.638611000	7.447206000
N	0.264053000	6.188786000	7.397550000
H	0.403122000	5.479238000	6.661466000
N	-2.265562000	6.455112000	10.246504000
N	-1.257845000	4.425606000	10.575899000
N	0.678440000	3.990343000	9.669866000
N	1.715808000	7.272113000	10.370590000
N	0.629860000	8.818066000	9.310633000
N	-1.306424000	8.428967000	8.143686000
C	-3.185421000	7.397503000	10.538642000
H	-3.106780000	8.339134000	9.997553000
C	-4.185849000	7.189747000	11.486573000
H	-4.901680000	7.984688000	11.696285000
C	-4.252608000	5.963181000	12.148109000
H	-5.032228000	5.768993000	12.886213000
C	-3.302302000	4.979654000	11.861533000
H	-3.324071000	4.014715000	12.367708000
C	-2.331427000	5.281414000	10.917409000
C	-0.340424000	4.889791000	9.654209000
C	-0.842043000	3.230704000	11.109985000
H	-1.411308000	2.653986000	11.832200000
C	0.387044000	2.966456000	10.540480000
H	1.060469000	2.129616000	10.706925000
C	2.025409000	4.296803000	9.150965000
H	1.887371000	4.950651000	8.282768000
H	2.497154000	3.363372000	8.820468000
C	2.862056000	4.989963000	10.239454000
H	3.239027000	4.241209000	10.953074000
H	3.749917000	5.423192000	9.755525000
C	2.130352000	6.041714000	11.076545000
H	1.240141000	5.630231000	11.569902000
H	2.796750000	6.369321000	11.884076000
C	2.453945000	8.449822000	10.466235000
H	3.382265000	8.502657000	11.026032000
C	1.779386000	9.422109000	9.796855000
H	2.020734000	10.468181000	9.647929000
C	0.585817000	7.497073000	9.663365000
C	-0.390783000	9.353287000	8.504422000
C	-0.456405000	10.688695000	8.125171000
H	0.306281000	11.401519000	8.436009000
C	-1.530250000	11.091040000	7.332442000
H	-1.613118000	12.130410000	7.011588000
C	-2.493612000	10.151098000	6.959967000
H	-3.348603000	10.426934000	6.342829000
C	-2.346696000	8.835856000	7.383439000
H	-3.087976000	8.087575000	7.106975000

CCSD identifier	E	charge	Spin
$[^{Me}(NCCN)Fe(NCCH_3)(NH^{**})]$	-2515.94010598	+2	1

Fe	6.911579000	4.529756000	8.619198000
N	8.235743000	5.797789000	7.579417000
H	8.118789000	5.709749000	6.565635000
H	9.233273000	5.660519000	7.771552000
H	7.997359000	6.767066000	7.810548000
N	5.683161000	5.711645000	8.321185000
H	5.318980000	6.154000000	7.465385000
N	8.542699000	3.214992000	8.586419000
N	7.500690000	2.657615000	6.628371000
N	5.437734000	3.233332000	6.299140000
N	4.746325000	2.593516000	9.693038000
N	5.696848000	3.961697000	11.063783000
N	7.438403000	5.340079000	10.480220000
C	9.552752000	3.116511000	9.475938000
H	9.435816000	3.693653000	10.393260000
C	10.684141000	2.323251000	9.274526000
C	11.763005000	2.227831000	10.310688000
H	12.739456000	2.503001000	9.886794000
H	11.853768000	1.196922000	10.683330000
H	11.562953000	2.882284000	11.167914000
C	10.756369000	1.617335000	8.061679000
H	11.624229000	0.991144000	7.847734000
C	9.729368000	1.706048000	7.129574000
H	9.778458000	1.163065000	6.186666000
C	8.636974000	2.509900000	7.438975000
C	6.503027000	3.482883000	7.098469000
C	7.058050000	1.909233000	5.538540000
H	7.684239000	1.208267000	5.000509000
C	5.764792000	2.273583000	5.338929000
H	5.053076000	1.942763000	4.590720000
C	4.061421000	3.540369000	6.705963000
H	3.459867000	3.749235000	5.812495000
H	4.106391000	4.445621000	7.321198000
C	3.466140000	2.369002000	7.504405000
H	2.535924000	2.712311000	7.980578000
H	3.183255000	1.556261000	6.817900000
C	4.403185000	1.744986000	8.540971000
H	3.928313000	0.846021000	8.953131000
H	5.343204000	1.417117000	8.079832000
C	4.098516000	2.473148000	10.921742000
H	3.284442000	1.774347000	11.076097000
C	4.693034000	3.332627000	11.787953000
H	4.491937000	3.527393000	12.833690000
C	5.732384000	3.519022000	9.765412000
C	6.612247000	4.947937000	11.467619000
C	6.667765000	5.472257000	12.755412000
H	5.979604000	5.136980000	13.529661000
C	7.624374000	6.442995000	13.025423000
H	7.686300000	6.873788000	14.026231000
C	9.547486000	7.921723000	12.274040000
H	10.176545000	8.092391000	11.391727000
H	10.199339000	7.635908000	13.111923000
H	9.075849000	8.877693000	12.545375000
C	8.507173000	6.872975000	12.021714000
C	8.360382000	6.279369000	10.766582000
H	9.021665000	6.575713000	9.951500000

CCSD identifier	E	charge	Spin
$[^{Me}(NCCN)Fe(NCCH_3)(NH^{**})]$	-2515.45915168	+3	3/2

Fe	6.898301000	4.570369000	8.636682000
N	7.939312000	5.604073000	7.633978000
H	8.305490000	5.812718000	6.694219000
N	5.477287000	6.045435000	8.511289000
H	5.215722000	6.224382000	7.535685000
H	5.922210000	6.911843000	8.837846000
H	4.617130000	5.916722000	9.055012000
N	8.454737000	3.246524000	8.616272000
N	7.464866000	2.722320000	6.614611000
N	5.371169000	3.215986000	6.316919000
N	4.693907000	2.542863000	9.712934000
N	5.675495000	3.891846000	11.088534000
N	7.348042000	5.337228000	10.448442000
C	9.445960000	3.168119000	9.523030000
H	9.302268000	3.717019000	10.451585000
C	10.614156000	2.418204000	9.314607000
C	11.670778000	2.329871000	10.367433000
H	12.642917000	2.653501000	9.965029000
H	11.799779000	1.287464000	10.697636000
H	11.436339000	2.942444000	11.245817000
C	10.736647000	1.757922000	8.080634000
H	11.633512000	1.175681000	7.860660000
C	9.720449000	1.835344000	7.134363000
H	9.805435000	1.321138000	6.177073000
C	8.594769000	2.588027000	7.443213000
C	6.438106000	3.495845000	7.095302000
C	7.049703000	1.989490000	5.516257000
H	7.690676000	1.307355000	4.968215000
C	5.734228000	2.312419000	5.324564000
H	5.037164000	1.976945000	4.562290000
C	3.979563000	3.495747000	6.703062000
H	3.387368000	3.670552000	5.796069000
H	3.975686000	4.424076000	7.280694000
C	3.398119000	2.327429000	7.517993000
H	2.462366000	2.667078000	7.986123000
H	3.115832000	1.512599000	6.834033000
C	4.332393000	1.698930000	8.554123000
H	3.844339000	0.808399000	8.968495000
H	5.268105000	1.351557000	8.097671000
C	4.118571000	2.357516000	10.969254000
H	3.332570000	1.629443000	11.142766000
C	4.733629000	3.202942000	11.839051000
H	4.578352000	3.350527000	12.901645000
C	5.659709000	3.488258000	9.770667000
C	6.597002000	4.877079000	11.480196000
C	6.744789000	5.352721000	12.777873000
H	6.133751000	4.968081000	13.593775000
C	7.698772000	6.338883000	13.006717000
H	7.832862000	6.728480000	14.018174000
C	9.515115000	7.907275000	12.179461000

H	10.069207000	8.152669000	11.266230000
H	10.232875000	7.598214000	12.954941000
H	9.035024000	8.826250000	12.551704000
C	8.493638000	6.841664000	11.953565000
C	8.273110000	6.296723000	10.685236000
H	8.841926000	6.626561000	9.814402000

CCSD identifier	E	charge	Spin	Fe-N-C
$[^{NMe2}(NCCN)Fe(NCCH_3)(N^{***})]$	-2436.79834790	+2	3/2	bonded

Fe	4.264032000	10.100352000	1.628414000
N	5.378497000	8.924555000	0.355038000
N	4.991982000	9.906896000	3.243589000
N	3.072823000	11.046709000	0.232721000
N	1.101467000	13.266192000	-2.734289000
N	4.616257000	12.705366000	0.588085000
N	6.116608000	12.493667000	2.135520000
N	4.181566000	11.226193000	5.101295000
N	2.838044000	9.660665000	4.283697000
N	2.878200000	8.659729000	2.157434000
N	0.218818000	5.671593000	3.459069000
C	6.134975000	8.254855000	-0.217368000
C	7.085372000	7.423550000	-0.924435000
H	6.580722000	6.877021000	-1.733656000
H	7.531740000	6.697169000	-0.230323000
H	7.883894000	8.043393000	-1.356083000
C	1.962902000	10.578405000	-0.391885000
H	1.615938000	9.597324000	-0.070059000
C	1.279343000	11.269202000	-1.364194000
H	0.396013000	10.814336000	-1.804500000
C	1.731151000	12.558308000	-1.775379000
C	-0.089831000	12.725618000	-3.389998000
H	0.133896000	11.775323000	-3.897152000
H	-0.436460000	13.439626000	-4.141836000
H	-0.903598000	12.564889000	-2.666955000
C	1.590197000	14.588326000	-3.121680000
H	1.552663000	15.290971000	-2.274866000
H	0.955620000	14.982819000	-3.919492000
H	2.622085000	14.534617000	-3.500907000
C	2.886966000	13.059154000	-1.110787000
H	3.298319000	14.034459000	-1.355407000
C	3.490371000	12.285829000	-0.144405000
C	5.154194000	11.802642000	1.472674000
C	5.241771000	13.944457000	0.690607000
H	4.979555000	14.802268000	0.084500000
C	6.184281000	13.804420000	1.661962000
H	6.904963000	14.518633000	2.043894000
C	6.962520000	11.946931000	3.206193000
H	7.976017000	12.341567000	3.050777000
H	6.980959000	10.865397000	3.060335000
C	6.514842000	12.287638000	4.647530000

H	6.986042000	11.551969000	5.315718000
H	6.946160000	13.262772000	4.922652000
C	5.028314000	12.433499000	4.985141000
H	4.960131000	12.926983000	5.963100000
H	4.541284000	13.101633000	4.255079000
C	3.016582000	11.203699000	5.862641000
H	2.857441000	11.893022000	6.684152000
C	2.196777000	10.231690000	5.378948000
H	1.213263000	9.921878000	5.709668000
C	4.098289000	10.257138000	4.125245000
C	2.393684000	8.628467000	3.430633000
C	1.532407000	7.657636000	3.909450000
H	1.247410000	7.676400000	4.957893000
C	-0.289359000	5.679482000	4.828572000
H	0.510634000	5.479035000	5.559136000
H	-1.049758000	4.900578000	4.931571000
H	-0.759952000	6.645182000	5.066405000
C	-0.163191000	4.580768000	2.562695000
H	-0.640898000	4.967258000	1.650191000
H	-0.882317000	3.936472000	3.075544000
H	0.707671000	3.968996000	2.281165000
C	1.083472000	6.621812000	3.046274000
C	1.628512000	6.633141000	1.729193000
H	1.381261000	5.858450000	1.007902000
C	2.499592000	7.630278000	1.357165000
H	2.938377000	7.630001000	0.358857000

CCSD identifier	E	charge	Spin	Fe-N-C
$[^{NMe2}(NCCN)Fe(NCCH_3)(N^{***})]$	-2780.26724956	+3	2	bonded

Fe	4.229633000	10.095246000	1.616519000
N	5.376792000	8.892254000	0.404712000
N	4.902791000	9.953148000	3.250665000
N	3.100239000	11.031856000	0.167371000
N	1.175664000	13.272531000	-2.821580000
N	4.558510000	12.750803000	0.640244000
N	6.039959000	12.536836000	2.212406000
N	4.166807000	11.238182000	5.174544000
N	2.812077000	9.679392000	4.370165000
N	2.789533000	8.744309000	2.203479000
N	0.235727000	5.671854000	3.515017000
C	6.158571000	8.251992000	-0.168415000
C	7.128844000	7.454422000	-0.876868000
H	7.672543000	6.811912000	-0.168064000
H	7.847500000	8.109373000	-1.392145000
H	6.624901000	6.821786000	-1.622725000
C	2.021871000	10.552503000	-0.514772000
H	1.690618000	9.552463000	-0.243471000
C	1.355049000	11.248647000	-1.491218000
H	0.513144000	10.771698000	-1.986493000
C	1.780688000	12.563157000	-1.855842000
C	-0.013903000	12.746671000	-3.502476000
H	0.249921000	11.900937000	-4.155597000
H	-0.447905000	13.538945000	-4.117821000
H	-0.769070000	12.425491000	-2.772622000
C	1.673602000	14.592533000	-3.222177000
H	1.433766000	15.353046000	-2.462925000
H	1.193991000	14.879693000	-4.161637000
H	2.758454000	14.564737000	-3.390501000
C	2.890844000	13.083502000	-1.120201000
H	3.264749000	14.086787000	-1.306764000

C	3.477126000	12.303244000	-0.155150000
C	5.074187000	11.862285000	1.540770000
C	5.197504000	13.974071000	0.734878000
H	4.960568000	14.832226000	0.117174000
C	6.133992000	13.831247000	1.723053000
H	6.865541000	14.541218000	2.095464000
C	6.912198000	11.968553000	3.261831000
H	7.921182000	12.361852000	3.080334000
H	6.932829000	10.890223000	3.093206000
C	6.510510000	12.289442000	4.719059000
H	7.006299000	11.543984000	5.358040000
H	6.952776000	13.258165000	4.998215000
C	5.040579000	12.437339000	5.112352000
H	5.010520000	12.865696000	6.122126000
H	4.534855000	13.154744000	4.445674000
C	3.047463000	11.171230000	5.988002000
H	2.913681000	11.834049000	6.836763000
C	2.213935000	10.200757000	5.506583000
H	1.247124000	9.873730000	5.870915000
C	4.048252000	10.297753000	4.183246000
C	2.352679000	8.664750000	3.497054000
C	1.522635000	7.672134000	3.974180000
H	1.273772000	7.653915000	5.032204000
C	-0.230104000	5.628123000	4.904566000
H	0.610388000	5.462498000	5.595692000
H	-0.935421000	4.801416000	5.018010000
H	-0.748591000	6.560378000	5.171685000
C	-0.192532000	4.612995000	2.593517000
H	-0.773566000	5.032187000	1.759170000
H	-0.825639000	3.906043000	3.134859000
H	0.675605000	4.066734000	2.196352000
C	1.053424000	6.652283000	3.094573000
C	1.532511000	6.724210000	1.750999000
H	1.265350000	5.971182000	1.013687000
C	2.369823000	7.744410000	1.375823000
H	2.768384000	7.774427000	0.361595000

CCSD identifier/Name	E	charge	Spin	Fe-N-C
$[^{H}(NCCN)Fe(NCCH_3)(N^{***})]$	-2436.71563436	+2	3/2	bonded

Fe	-1.045796000	6.462200000	8.885691000
N	-1.969150000	5.577644000	7.126560000
H	-2.244595000	6.235122000	6.389818000
H	-2.776551000	4.971298000	7.299367000
H	-1.248437000	4.980913000	6.708574000
N	0.646404000	6.618991000	8.371515000
N	-2.633815000	6.344835000	10.243899000
N	-1.423783000	4.498966000	10.882021000
N	0.508934000	4.064690000	10.002774000
N	2.320348000	7.371512000	9.953845000
N	0.836767000	8.851710000	9.228571000
N	-1.304458000	8.465104000	8.329173000
C	-3.685733000	7.183530000	10.372444000
H	-3.692564000	8.050877000	9.713795000
C	-4.701685000	6.977925000	11.295516000
H	-5.527399000	7.685037000	11.359856000
C	-4.636754000	5.858146000	12.128528000
H	-5.420567000	5.664437000	12.861217000
C	-3.555590000	4.986691000	12.020734000
H	-3.476152000	4.107250000	12.657979000
C	-2.577496000	5.270751000	11.073102000

C	-0.565834000	4.884415000	9.876514000
C	-0.891516000	3.444291000	11.622058000
H	-1.399075000	2.978099000	12.457419000
C	0.318523000	3.176131000	11.063011000
H	1.052048000	2.419354000	11.318299000
C	1.708614000	4.093690000	9.149634000
H	1.406377000	4.544473000	8.202744000
H	1.997028000	3.050170000	8.964375000
C	2.924823000	4.845853000	9.735159000
H	3.474606000	4.157507000	10.395814000
H	3.604464000	5.061675000	8.897589000
C	2.722924000	6.096496000	10.592643000
H	1.987524000	5.894442000	11.388971000
H	3.677549000	6.313693000	11.088277000
C	2.630954000	8.612273000	10.502380000
H	3.459253000	8.742251000	11.190156000
C	1.737526000	9.531462000	10.046167000
H	1.660293000	10.591698000	10.254654000
C	1.223490000	7.505742000	9.137742000
C	-0.289477000	9.345003000	8.547646000
C	-0.336918000	10.672321000	8.117207000
H	0.517042000	11.328540000	8.280361000
C	-1.470147000	11.123526000	7.450313000
H	-1.528394000	12.156398000	7.106427000
C	-2.513164000	10.227287000	7.204301000
H	-3.413857000	10.533775000	6.673918000
C	-2.381045000	8.918935000	7.646211000
H	-3.170276000	8.188235000	7.462587000

CCSD identifier	E	charge	Spin	Fe-N-C Not bonded
$[^{H}(NCCN)Fe(NCCH_3)(N^{***})]$	-2436.71563436	+2	3/2	

Fe	-0.791659000	6.502875000	8.796153000
N	-2.198902000	5.610639000	7.456010000
H	-2.028437000	6.035961000	6.538120000
H	-3.205984000	5.637420000	7.646818000
H	-1.940119000	4.625506000	7.342478000
N	0.395728000	6.330321000	7.576129000
N	-2.387185000	6.450091000	10.304180000
N	-1.293965000	4.463570000	10.676053000
N	0.624605000	4.000943000	9.769383000
N	1.800379000	7.294288000	10.318941000
N	0.703832000	8.842016000	9.280820000
N	-1.283788000	8.479438000	8.197591000
C	-3.371482000	7.333339000	10.558059000
H	-3.327843000	8.272169000	10.005293000
C	-4.389174000	7.082406000	11.474603000
H	-5.159793000	7.832166000	11.649786000

C	-4.397862000	5.863730000	12.154781000
H	-5.183845000	5.632257000	12.873877000
C	-3.383552000	4.938144000	11.909393000
H	-3.361730000	3.980182000	12.427220000
C	-2.405196000	5.286215000	10.984855000
C	-0.377490000	4.912084000	9.751816000
C	-0.871763000	3.274338000	11.241995000
H	-1.441846000	2.721312000	11.979026000
C	0.342679000	2.992664000	10.672463000
H	1.013474000	2.156420000	10.839413000
C	1.928567000	4.259757000	9.143740000
H	1.720298000	4.884377000	8.260615000
H	2.359191000	3.304211000	8.820732000
C	2.863431000	4.978073000	10.131526000
H	3.298874000	4.248259000	10.831786000
H	3.703678000	5.397234000	9.558996000
C	2.217042000	6.058824000	11.002823000
H	1.344616000	5.669557000	11.543651000
H	2.939907000	6.368134000	11.767667000
C	2.562368000	8.464659000	10.379666000
H	3.511773000	8.502210000	10.901374000
C	1.879469000	9.438180000	9.728889000
H	2.122273000	10.480459000	9.566134000
C	0.654930000	7.515446000	9.634686000
C	-0.337184000	9.385576000	8.512882000
C	-0.393336000	10.719136000	8.117298000
H	0.393083000	11.420724000	8.390378000
C	-1.487085000	11.128941000	7.358850000
H	-1.562417000	12.164923000	7.027820000
C	-2.480177000	10.203307000	7.030463000
H	-3.349683000	10.488953000	6.440061000
C	-2.339621000	8.892465000	7.469003000
H	-3.093818000	8.141466000	7.233257000

CCSD identifier	E	charge	Spin	Fe-N-C
$[^{H}(NCCN)Fe(NCCH_3)(N^{***})]$	-2436.34254445	+3	2	bonded

Fe	-1.120805000	6.422076000	8.831194000
N	-1.962847000	5.556890000	7.066557000
H	-1.757822000	6.124136000	6.234407000
H	-2.977249000	5.398024000	7.075517000
H	-1.538937000	4.639742000	6.880997000
N	0.586272000	6.650540000	8.442215000
N	-2.750926000	6.286481000	10.179651000
N	-1.412311000	4.583118000	10.976514000
N	0.527426000	4.134611000	10.095491000
N	2.373378000	7.362771000	9.938841000
N	0.880718000	8.861893000	9.274865000

N	-1.301147000	8.505610000	8.445650000
C	-3.868566000	7.045622000	10.229843000
H	-3.944601000	7.855114000	9.506679000
C	-4.882297000	6.833083000	11.158488000
H	-5.763861000	7.473882000	11.151216000
C	-4.743864000	5.799152000	12.084488000
H	-5.521050000	5.604516000	12.825036000
C	-3.589433000	5.011974000	12.059956000
H	-3.452019000	4.201696000	12.775381000
C	-2.630403000	5.294677000	11.099527000
C	-0.555283000	4.947253000	9.971454000
C	-0.882018000	3.548727000	11.719109000
H	-1.383226000	3.083206000	12.561180000
C	0.339193000	3.267082000	11.154507000
H	1.062643000	2.500416000	11.417046000
C	1.691640000	4.106500000	9.178177000
H	1.347958000	4.528441000	8.231707000
H	1.930622000	3.048264000	9.008246000
C	2.963841000	4.829129000	9.668861000
H	3.543788000	4.134159000	10.295797000
H	3.587064000	5.009895000	8.780432000
C	2.870757000	6.088365000	10.527213000
H	2.248260000	5.906905000	11.418376000
H	3.881608000	6.317642000	10.887702000
C	2.746538000	8.596855000	10.432943000
H	3.617743000	8.724314000	11.069013000
C	1.833036000	9.532700000	10.017955000
H	1.787161000	10.596678000	10.225439000
C	1.238404000	7.516438000	9.185044000
C	-0.266285000	9.371105000	8.622926000
C	-0.298147000	10.689888000	8.174795000
H	0.567701000	11.337884000	8.310356000
C	-1.434906000	11.147748000	7.512089000
H	-1.481996000	12.176345000	7.151270000
C	-2.490908000	10.261938000	7.290886000
H	-3.390968000	10.570029000	6.758613000
C	-2.379747000	8.960651000	7.763855000
H	-3.184344000	8.243737000	7.594556000

CCSD identifier	E	charge	Spin	Fe-N-C
$[^{Me}(NCCN)Fe(NCCH_3)(N^{***})]$	-2515.36208955	+2	3/2	bonded

Fe	7.137982000	4.384855000	8.593017000
N	7.757502000	6.166901000	7.515209000
H	6.897373000	6.521029000	7.084433000

H	8.422313000	6.018585000	6.750402000
H	8.124074000	6.940369000	8.078994000
N	5.398785000	4.692479000	8.789448000
N	8.886515000	3.239379000	8.509429000
N	7.696222000	2.284922000	6.790525000
N	5.649068000	2.864893000	6.380783000
N	3.991212000	2.976995000	9.764330000
N	5.421353000	4.027284000	11.093686000
N	7.406311000	5.156503000	10.520541000
C	9.979240000	3.327872000	9.300250000
H	9.925942000	4.061768000	10.104359000
C	11.117687000	2.537569000	9.142305000
C	12.299898000	2.679106000	10.052989000
H	13.197570000	2.965919000	9.485962000
H	12.525033000	1.725196000	10.551416000
H	12.128777000	3.436597000	10.827826000
C	11.098185000	1.604532000	8.091287000
H	11.963513000	0.962128000	7.918627000
C	9.983036000	1.486415000	7.269974000
H	9.961058000	0.762466000	6.456593000
C	8.895923000	2.318122000	7.516118000
C	6.729897000	3.202692000	7.131018000
C	7.225964000	1.388977000	5.832898000
H	7.822150000	0.586850000	5.415861000
C	5.942477000	1.760828000	5.578969000
H	5.220496000	1.344901000	4.885062000
C	4.349832000	3.555711000	6.400235000
H	3.994073000	3.601717000	5.362026000
H	4.544302000	4.571837000	6.747657000
C	3.254437000	2.894386000	7.267396000
H	2.483855000	3.657125000	7.452466000
H	2.763116000	2.111913000	6.668337000
C	3.621838000	2.181934000	8.570786000
H	2.751745000	1.588835000	8.879515000
H	4.445945000	1.471543000	8.391686000
C	3.813177000	2.506694000	11.062001000
H	3.093909000	1.729226000	11.294465000
C	4.673977000	3.161240000	11.888274000
H	4.824171000	3.063929000	12.956678000
C	4.969120000	3.942293000	9.769524000
C	6.464847000	4.894832000	11.462936000
C	6.509130000	5.464010000	12.737014000
H	5.713778000	5.259633000	13.452892000
C	7.555628000	6.316149000	13.058057000
H	7.599486000	6.768779000	14.050166000
C	9.678718000	7.546168000	12.397038000
H	10.347459000	7.658129000	11.534629000
H	10.271626000	7.179851000	13.247660000
H	9.306248000	8.543749000	12.672353000
C	8.539336000	6.618777000	12.101256000
C	8.398182000	6.015067000	10.852500000
H	9.129765000	6.219197000	10.067607000

CCSD identifier	E	charge	Spin	Fe-N-C Not bonded
$[^{Me}(NCCN)Fe(NCCH_3)(N^{***})]$	-2515.27975946	+2	3/2	

Fe	6.899192000	4.455196000	8.643971000
N	8.052403000	5.881799000	7.551735000
H	7.719341000	5.881445000	6.582700000
H	9.075113000	5.817079000	7.521270000
H	7.792231000	6.802127000	7.922402000
N	5.546878000	5.494639000	8.462681000
N	8.680445000	3.181352000	8.599261000
N	7.547715000	2.504846000	6.718587000
N	5.497638000	3.099244000	6.317697000
N	4.596661000	2.655583000	9.710335000
N	5.607758000	3.971145000	11.096511000
N	7.400826000	5.282622000	10.527779000
C	9.733041000	3.138356000	9.437452000
H	9.653238000	3.756360000	10.333388000
C	10.872423000	2.356141000	9.215506000
C	12.007093000	2.337808000	10.193617000
H	12.949169000	2.629934000	9.707324000
H	12.155942000	1.325591000	10.597703000
H	11.828977000	3.017647000	11.035755000
C	10.895687000	1.591543000	8.037761000
H	11.762487000	0.969443000	7.808262000
C	9.817111000	1.615922000	7.158824000
H	9.828333000	1.021898000	6.245755000
C	8.732385000	2.420129000	7.490312000
C	6.536386000	3.328647000	7.157081000
C	7.145379000	1.790627000	5.605133000
H	7.783869000	1.094871000	5.073857000
C	5.848343000	2.163059000	5.362918000
H	5.162360000	1.837267000	4.588214000
C	4.134271000	3.551803000	6.624179000
H	3.606217000	3.744441000	5.682318000
H	4.249888000	4.497118000	7.178059000
C	3.397379000	2.493901000	7.461665000
H	2.499785000	2.963885000	7.889472000
H	3.041790000	1.681270000	6.809005000
C	4.222034000	1.817072000	8.560369000
H	3.638613000	0.984128000	8.971735000
H	5.144151000	1.377040000	8.158209000
C	3.911919000	2.592571000	10.926889000
H	3.051707000	1.948028000	11.066116000
C	4.542549000	3.417013000	11.799701000
H	4.332931000	3.635609000	12.838963000
C	5.640429000	3.511404000	9.803374000
C	6.551476000	4.926137000	11.507324000
C	6.614325000	5.460406000	12.791264000
H	5.911487000	5.153261000	13.564097000
C	7.598920000	6.404074000	13.058369000
H	7.665377000	6.843826000	14.054996000
C	9.573455000	7.816526000	12.310104000
H	10.224597000	7.948022000	11.437138000
H	10.199006000	7.530202000	13.167584000
H	9.126031000	8.792429000	12.550138000
C	8.505520000	6.795374000	12.058753000
C	8.355801000	6.191134000	10.808837000
H	9.035169000	6.449862000	9.995015000

CCSD identifier	E	charge	Spin	Fe-N-C
$[^{Me}(NCCN)Fe(NCCH_3)(N^{***})]$	-2514.91434275	+3	2	bonded

Fe	7.201179000	4.444156000	8.553817000
N	7.787191000	6.203866000	7.488491000
H	7.265278000	6.277287000	6.607127000
H	8.779540000	6.269011000	7.234004000
H	7.557370000	7.056169000	8.014227000
N	5.465110000	4.641222000	8.817745000
N	8.969826000	3.280701000	8.479165000
N	7.695961000	2.201039000	6.885252000
N	5.646161000	2.794021000	6.451960000
N	3.924287000	2.997692000	9.746443000
N	5.371614000	3.985182000	11.105802000
N	7.409188000	5.052164000	10.572481000
C	10.100479000	3.423724000	9.206030000
H	10.096850000	4.213214000	9.956152000
C	11.235203000	2.616893000	9.051655000
C	12.459336000	2.826579000	9.884402000
H	13.327136000	3.049392000	9.245207000
H	12.706447000	1.912621000	10.445143000
H	12.338401000	3.648362000	10.600178000
C	11.161978000	1.604448000	8.079979000
H	12.016186000	0.945137000	7.913199000
C	10.002435000	1.427694000	7.328694000
H	9.940762000	0.639361000	6.578823000
C	8.935549000	2.282313000	7.563521000
C	6.733833000	3.124436000	7.199566000
C	7.227621000	1.308538000	5.945157000
H	7.814667000	0.494547000	5.533343000
C	5.935529000	1.691975000	5.671667000
H	5.225303000	1.273706000	4.964209000
C	4.372049000	3.548803000	6.407470000
H	4.068878000	3.592141000	5.352910000
H	4.605213000	4.564741000	6.731881000
C	3.207181000	2.963761000	7.234813000
H	2.481766000	3.777856000	7.380683000
H	2.684771000	2.211627000	6.623668000
C	3.469999000	2.235904000	8.551504000
H	2.531137000	1.757649000	8.858129000
H	4.207423000	1.429253000	8.409289000
C	3.670078000	2.573014000	11.035379000
H	2.898804000	1.842538000	11.261269000
C	4.555317000	3.188109000	11.884605000
H	4.669994000	3.089274000	12.958837000
C	4.952719000	3.904703000	9.777812000
C	6.440376000	4.823818000	11.495771000
C	6.467935000	5.404637000	12.762745000
H	5.657018000	5.223369000	13.468270000
C	7.517583000	6.252187000	13.092390000
H	7.546034000	6.716034000	14.080511000
C	9.653515000	7.466767000	12.452166000
H	10.340010000	7.575148000	11.603944000
H	10.226033000	7.108416000	13.320951000
H	9.272249000	8.464794000	12.716980000
C	8.520719000	6.539196000	12.149740000
C	8.403788000	5.908652000	10.906890000

H	9.152244000	6.101015000	10.135415000

CCSD identifier	E	charge	Spin	Transition State
$[^{H}(NCCN)Fe(NH_3)(NH**)]$	-2438.15383378	+2	1	

Fe	-0.222146000	-0.159449000	-0.749025000
N	-1.604157000	-1.117002000	-2.155592000
H	-1.373836000	-0.743145000	-3.073920000
H	-2.607667000	-1.007268000	-2.029924000
H	-1.434306000	-2.117483000	-2.215476000
N	1.185999000	-0.032215000	-1.767244000
H	2.088864000	-0.493089000	-1.757143000
N	-1.772112000	-0.246261000	0.738705000
N	-0.757402000	-2.273600000	1.067303000
N	1.156605000	-2.772751000	0.231021000
N	2.449735000	0.643701000	0.599299000
N	1.254659000	2.208268000	-0.329855000
N	-0.773145000	1.814100000	-1.317278000
C	-2.724253000	0.661246000	1.008398000
H	-2.637669000	1.611645000	0.503337000
C	-3.762321000	0.417962000	1.891479000
H	-4.498665000	1.185999000	2.082005000
C	-3.829385000	-0.819740000	2.519740000
H	-4.631153000	-1.047119000	3.209937000
C	-2.848988000	-1.766802000	2.258586000
H	-2.873309000	-2.735752000	2.735636000
C	-1.839909000	-1.427409000	1.371822000
C	0.179785000	-1.849867000	0.169639000
C	-0.369037000	-3.462981000	1.673287000
H	-0.959936000	-3.980046000	2.406969000
C	0.832466000	-3.771193000	1.145155000
H	1.479524000	-4.611064000	1.330447000
C	2.477388000	-2.639411000	-0.388380000
H	2.328238000	-2.281767000	-1.401764000
H	2.901471000	-3.638523000	-0.474885000
C	3.443863000	-1.746204000	0.428638000
H	3.960137000	-2.384080000	1.149658000
H	4.219210000	-1.373843000	-0.244961000
C	2.866965000	-0.598237000	1.271883000
H	2.028152000	-0.946094000	1.875348000
H	3.642604000	-0.297245000	1.973794000
C	3.100094000	1.860353000	0.796389000
H	4.020077000	1.930674000	1.350232000
C	2.367081000	2.833611000	0.219963000
H	2.536138000	3.893849000	0.176597000
C	1.311550000	0.848677000	-0.118601000
C	0.175085000	2.727993000	-1.043058000
C	0.093099000	4.054602000	-1.449137000
H	0.881702000	4.756331000	-1.219564000
C	-1.021629000	4.448502000	-2.169755000
H	-1.116223000	5.473708000	-2.502778000

C	-2.008551000	3.512299000	-2.468195000
H	-2.888457000	3.782675000	-3.034606000
C	-1.840223000	2.213171000	-2.029028000
H	-2.586010000	1.460275000	-2.245096000

CCSD identifier	E	charge	Mult	MECP
$[{}^{H}(NCCN)Fe(NH_3)(NH**)]$	-2438.32098494604	+2	1&3	

Fe	-0.492483000	-0.173759000	-0.677544000
N	-1.315683000	-1.079032000	-2.207015000
H	-0.967567000	-0.659909000	-3.067896000
H	-2.331995000	-1.023083000	-2.229791000
H	-1.086969000	-2.068435000	-2.252548000
N	1.342626000	0.109622000	-1.584275000
H	1.958639000	-0.631230000	-1.875724000
N	-2.097679000	-0.368037000	0.562596000
N	-0.733529000	-2.012899000	1.412549000
N	1.134379000	-2.551136000	0.505576000
N	2.809415000	0.599692000	0.328370000
N	1.143851000	1.955051000	-0.154742000
N	-0.837652000	1.779276000	-1.361148000
C	-3.224117000	0.362213000	0.577552000
H	-3.312493000	1.114479000	-0.192454000
C	-4.220344000	0.187213000	1.521957000
H	-5.109588000	0.799950000	1.487798000
C	-4.047300000	-0.781038000	2.502159000
H	-4.804628000	-0.944646000	3.256796000
C	-2.887006000	-1.543997000	2.506714000
H	-2.726850000	-2.305325000	3.255879000
C	-1.946871000	-1.304118000	1.522406000
C	0.072523000	-1.733585000	0.343462000
C	-0.178680000	-2.989990000	2.230326000
H	-0.647319000	-3.361238000	3.123152000
C	0.995951000	-3.327491000	1.654864000
H	1.729173000	-4.057685000	1.949616000
C	2.217358000	-2.697511000	-0.472002000
H	1.774881000	-2.495076000	-1.441747000
H	2.496478000	-3.749534000	-0.479794000
C	3.497248000	-1.847087000	-0.249621000
H	4.260506000	-2.504764000	0.169194000
H	3.887081000	-1.546851000	-1.224223000
C	3.482507000	-0.663033000	0.711126000
H	3.043669000	-0.969648000	1.663185000
H	4.520626000	-0.396323000	0.905955000

C	2.838960000	1.678320000	1.226261000
H	3.575932000	1.733640000	2.007892000
C	1.830947000	2.510450000	0.932994000
H	1.518594000	3.428896000	1.395409000
C	1.806908000	0.797350000	-0.567905000
C	0.147605000	2.589582000	-0.945320000
C	0.209014000	3.935929000	-1.253525000
H	1.036183000	4.541095000	-0.911031000
C	-0.806646000	4.473608000	-2.035180000
H	-0.792526000	5.523062000	-2.296578000
C	-1.820429000	3.642831000	-2.492141000
H	-2.618937000	4.020951000	-3.114511000
C	-1.793487000	2.303629000	-2.137952000
H	-2.565650000	1.626297000	-2.479136000

CCSD identifier	E	charge	Mult	MECP
$[^{H}(NCCN)Fe(NH_3)(NH**)]$	-2438.327299862158	+2	1&5	

Fe	-0.491139000	-0.207003000	-0.693262000
N	-1.458164000	-1.055861000	-2.385365000
H	-1.202669000	-0.558303000	-3.235375000
H	-2.474315000	-1.072479000	-2.344737000
H	-1.171458000	-2.021307000	-2.520392000
N	1.329100000	0.056561000	-1.450358000
H	1.923268000	-0.734933000	-1.627378000
N	-2.060998000	-0.362873000	0.645240000
N	-0.804438000	-2.167015000	1.313133000
N	1.096506000	-2.658703000	0.451980000
N	2.926315000	0.699358000	0.262047000
N	1.385914000	2.145666000	-0.359340000
N	-0.746754000	1.805270000	-1.266673000
C	-3.133558000	0.439218000	0.753584000
H	-3.162990000	1.289338000	0.088785000
C	-4.146779000	0.216805000	1.666805000
H	-4.988391000	0.892570000	1.715155000
C	-4.053114000	-0.883107000	2.511548000
H	-4.829872000	-1.091225000	3.234959000
C	-2.948193000	-1.716860000	2.421951000
H	-2.850229000	-2.579383000	3.064293000
C	-1.978423000	-1.414936000	1.481630000
C	0.036546000	-1.836884000	0.285912000
C	-0.276708000	-3.182339000	2.103794000
H	-0.776253000	-3.594134000	2.961131000
C	0.918019000	-3.488335000	1.557714000
H	1.645191000	-4.227279000	1.845483000
C	2.274489000	-2.711700000	-0.418053000
H	1.931971000	-2.487965000	-1.423731000
H	2.599335000	-3.750389000	-0.449471000
C	3.490940000	-1.843289000	0.008662000
H	4.141958000	-2.482786000	0.606746000
H	4.067250000	-1.603969000	-0.887584000

C	3.302919000	-0.596943000	0.878354000
H	2.579161000	-0.799824000	1.672128000
H	4.258349000	-0.403647000	1.361634000
C	3.117587000	1.890592000	0.986632000
H	3.908595000	1.986614000	1.708983000
C	2.185254000	2.776532000	0.608699000
H	2.005255000	3.786315000	0.928662000
C	1.877482000	0.880735000	-0.584446000
C	0.262283000	2.666276000	-1.032325000
C	0.225740000	3.997422000	-1.419185000
H	1.073851000	4.640510000	-1.233015000
C	-0.901488000	4.467746000	-2.074409000
H	-0.956521000	5.501401000	-2.388221000
C	-1.940650000	3.586042000	-2.344530000
H	-2.830013000	3.906482000	-2.868089000
C	-1.815241000	2.272196000	-1.933357000
H	-2.600289000	1.556496000	-2.136367000

CCSD identifier	E	charge	Mult	MECP
$[^{H}(NCCN)Fe(NH_3)(NH**)]$	-2438.15383378	+2	3&5	

Fe	-0.527273000	-0.252337000	-0.805073000
N	-1.566047000	-1.118351000	-2.550502000
H	-1.379746000	-0.607563000	-3.410368000
H	-2.577238000	-1.184260000	-2.462730000
H	-1.244354000	-2.069516000	-2.709314000
N	1.345695000	0.054386000	-1.470933000
H	1.948745000	-0.729073000	-1.657589000
N	-2.073948000	-0.378794000	0.642112000
N	-0.771930000	-2.146327000	1.340535000
N	1.115509000	-2.667228000	0.470466000
N	2.895386000	0.676034000	0.294012000
N	1.369743000	2.127994000	-0.346022000
N	-0.750517000	1.832328000	-1.306063000
C	-3.161008000	0.404324000	0.740332000
H	-3.217382000	1.232270000	0.048618000
C	-4.155099000	0.190259000	1.675723000
H	-5.010342000	0.849119000	1.717910000
C	-4.024051000	-0.882140000	2.551072000
H	-4.785167000	-1.084634000	3.292545000
C	-2.904994000	-1.696982000	2.467787000
H	-2.782872000	-2.540215000	3.131145000
C	-1.955093000	-1.404307000	1.503220000
C	0.039846000	-1.876597000	0.274319000
C	-0.208796000	-3.099511000	2.184236000
H	-0.681544000	-3.459464000	3.079220000
C	0.978155000	-3.424233000	1.632147000
H	1.723045000	-4.132190000	1.951085000

C	2.280273000	-2.733217000	-0.414389000
H	1.921004000	-2.517144000	-1.415886000
H	2.607394000	-3.771407000	-0.439596000
C	3.496129000	-1.851209000	-0.012880000
H	4.176795000	-2.486620000	0.555574000
H	4.042407000	-1.586538000	-0.920656000
C	3.307345000	-0.622254000	0.880893000
H	2.605321000	-0.849442000	1.687120000
H	4.269994000	-0.419695000	1.345913000
C	3.065529000	1.855146000	1.041481000
H	3.835894000	1.940266000	1.787055000
C	2.141446000	2.744680000	0.652954000
H	1.949695000	3.748197000	0.985178000
C	1.867047000	0.864738000	-0.575530000
C	0.263879000	2.671711000	-1.030678000
C	0.248691000	4.013642000	-1.384482000
H	1.100262000	4.642775000	-1.168946000
C	-0.861974000	4.513133000	-2.045548000
H	-0.899099000	5.554903000	-2.334339000
C	-1.908810000	3.652373000	-2.354063000
H	-2.785715000	3.997839000	-2.882688000
C	-1.804357000	2.327417000	-1.975711000
H	-2.593244000	1.624387000	-2.208682000

CCSD identifier	E	charge	Spin	Fe-N-C
$[^{H}(NCCN)Fe(NH_3)(NH**)]$	-2438.31943373	+2	0	bonded

Fe	-1.041249000	6.523332000	8.930587000
N	-1.891795000	5.646925000	7.342275000
H	-1.531608000	6.074446000	6.491160000
H	-2.906502000	5.716811000	7.310916000
H	-1.677296000	4.655415000	7.283970000
N	0.761791000	6.773638000	8.063494000
N	-2.635524000	6.352002000	10.185649000
N	-1.344233000	4.612073000	10.947726000
N	0.540807000	4.091385000	10.066520000
N	2.293941000	7.350773000	9.882360000
N	0.712333000	8.766480000	9.308562000
N	-1.356159000	8.472231000	8.273551000
C	-3.729696000	7.129165000	10.244862000
H	-3.779081000	7.937736000	9.531371000
C	-4.741707000	6.933104000	11.166882000
H	-5.601890000	7.586872000	11.170517000
C	-4.623042000	5.890187000	12.076779000
H	-5.396155000	5.706142000	12.810398000
C	-3.495938000	5.081258000	12.038391000
H	-3.376466000	4.262902000	12.732925000

C	-2.533341000	5.350623000	11.082778000
C	-0.510060000	4.926461000	9.910315000
C	-0.819272000	3.597385000	11.739198000
H	-1.313595000	3.196207000	12.604705000
C	0.365850000	3.272091000	11.180420000
H	1.086274000	2.525276000	11.464750000
C	1.674476000	3.988773000	9.143557000
H	1.283932000	4.195125000	8.152100000
H	1.976508000	2.943115000	9.129075000
C	2.924660000	4.852752000	9.466707000
H	3.643785000	4.204557000	9.969526000
H	3.401951000	5.133616000	8.525701000
C	2.815301000	6.061671000	10.394483000
H	2.219524000	5.802398000	11.273203000
H	3.821409000	6.284455000	10.745404000
C	2.419363000	8.502091000	10.677279000
H	3.182482000	8.582018000	11.430966000
C	1.459355000	9.370566000	10.328154000
H	1.222203000	10.347373000	10.708123000
C	1.270427000	7.536075000	9.006398000
C	-0.357718000	9.322320000	8.570640000
C	-0.353000000	10.653024000	8.189227000
H	0.486877000	11.287890000	8.432640000
C	-1.434520000	11.134183000	7.464431000
H	-1.461601000	12.168936000	7.150747000
C	-2.462100000	10.262727000	7.128211000
H	-3.313846000	10.593089000	6.550972000
C	-2.377631000	8.945735000	7.544825000
H	-3.158450000	8.239476000	7.296486000
H	1.358943000	5.994531000	7.844019000

CCSD identifier	E	charge	Spin	Fe-N-C
$[^{H}(NCCN)Fe(NH_3)(NH**)]$	-2438.34013838	+2	1	bonded

Fe	-1.057084000	6.481541000	8.862155000
N	-2.088156000	5.539369000	7.124350000
H	-1.940070000	6.048290000	6.256272000
H	-3.092871000	5.420052000	7.227205000
H	-1.716372000	4.606005000	6.971989000
N	0.766373000	6.748514000	8.176079000
N	-2.594897000	6.361508000	10.224950000
N	-1.378406000	4.534721000	10.876862000
N	0.526298000	4.045861000	10.018061000
N	2.347189000	7.406912000	9.890242000
N	0.826028000	8.855614000	9.233837000
N	-1.293188000	8.491700000	8.302230000
C	-3.650263000	7.187148000	10.337574000
H	-3.660276000	8.043021000	9.679917000

C	-4.668048000	6.978983000	11.247828000
H	-5.494726000	7.672546000	11.300967000
C	-4.599476000	5.870105000	12.084638000
H	-5.381747000	5.674107000	12.805494000
C	-3.513878000	5.013003000	11.990588000
H	-3.435339000	4.143489000	12.626243000
C	-2.536194000	5.301035000	11.053350000
C	-0.532676000	4.871698000	9.853342000
C	-0.854669000	3.513055000	11.662650000
H	-1.359142000	3.095551000	12.514320000
C	0.340740000	3.209919000	11.118958000
H	1.065901000	2.468340000	11.404871000
C	1.720699000	3.998735000	9.171044000
H	1.400817000	4.216365000	8.156205000
H	2.050309000	2.961352000	9.147932000
C	2.925956000	4.871500000	9.624868000
H	3.571485000	4.229281000	10.225850000
H	3.514838000	5.124659000	8.740674000
C	2.717271000	6.108432000	10.504480000
H	1.977848000	5.896365000	11.281139000
H	3.662079000	6.301203000	11.008192000
C	2.536927000	8.603332000	10.606910000
H	3.318733000	8.702222000	11.338772000
C	1.615269000	9.491583000	10.207307000
H	1.438703000	10.505978000	10.514239000
C	1.308225000	7.585912000	9.035612000
C	-0.294457000	9.362355000	8.547140000
C	-0.338605000	10.691091000	8.152649000
H	0.500620000	11.343044000	8.348003000
C	-1.459466000	11.147106000	7.476972000
H	-1.519051000	12.178680000	7.157195000
C	-2.485455000	10.253966000	7.193387000
H	-3.368772000	10.562891000	6.652989000
C	-2.354951000	8.943914000	7.613916000
H	-3.128465000	8.218056000	7.402881000
H	1.363110000	5.957304000	8.010607000

CCSD identifier	E	charge	Spin	Fe-N-C
$[^{H}(NCCN)Fe(NH_3)(NH **)]$	-2438.34458542	+2	2	bonded

Fe	-1.155539000	6.335743000	8.513270000
N	-2.012892000	5.662858000	6.656581000
H	-1.613848000	6.157408000	5.860843000
H	-3.024328000	5.735588000	6.571635000
H	-1.795673000	4.678039000	6.515982000

N	0.862837000	6.707816000	7.997270000
N	-2.844511000	6.188314000	10.103634000
N	-1.303690000	4.744501000	11.064063000
N	0.566307000	4.141274000	10.214320000
N	2.341902000	7.277601000	9.854354000
N	0.776868000	8.703254000	9.265051000
N	-1.336713000	8.504658000	8.266208000
C	-4.017321000	6.835177000	10.109686000
H	-4.213612000	7.480970000	9.264640000
C	-4.944677000	6.708627000	11.129345000
H	-5.879605000	7.248547000	11.085607000
C	-4.638767000	5.876510000	12.198164000
H	-5.335198000	5.748768000	13.015866000
C	-3.424849000	5.203943000	12.210957000
H	-3.168384000	4.550460000	13.031020000
C	-2.564606000	5.393234000	11.141609000
C	-0.521169000	4.893218000	9.963083000
C	-0.707266000	3.904724000	11.995990000
H	-1.151103000	3.641867000	12.938134000
C	0.471792000	3.524494000	11.454553000
H	1.228685000	2.860514000	11.834447000
C	1.655317000	3.933892000	9.255192000
H	1.227514000	4.121879000	8.276750000
H	1.909295000	2.875398000	9.280102000
C	2.945376000	4.762603000	9.485398000
H	3.669043000	4.115793000	9.983828000
H	3.389324000	4.989842000	8.514166000
C	2.904722000	6.007911000	10.366116000
H	2.378353000	5.781194000	11.296360000
H	3.935257000	6.239458000	10.631815000
C	2.455740000	8.418219000	10.666118000
H	3.206650000	8.486604000	11.432877000
C	1.506639000	9.290767000	10.310591000
H	1.264230000	10.262467000	10.698890000
C	1.326236000	7.467028000	8.961824000
C	-0.305341000	9.299426000	8.591222000
C	-0.291099000	10.661259000	8.315456000
H	0.573695000	11.257741000	8.567429000
C	-1.389376000	11.224192000	7.686876000
H	-1.401181000	12.281273000	7.457538000
C	-2.460019000	10.409183000	7.336274000
H	-3.331025000	10.806343000	6.835037000
C	-2.384263000	9.063548000	7.636934000
H	-3.193308000	8.396293000	7.368601000
H	1.512297000	5.976996000	7.752230000

CCSD identifier	E	charge	Spin

TEMPO ·	-483.28319576	0	1/2

C	0.228002000	0.045206000	-2.544194000
C	0.478504000	1.284335000	-1.670169000
C	-0.237642000	1.231133000	-0.325838000
C	0.249114000	0.009577000	0.444892000
C	-0.019966000	-1.317296000	-0.281789000
H	0.178276000	2.171174000	-2.249549000
H	1.563089000	1.374934000	-1.490128000
H	-0.030326000	2.146740000	0.248541000
H	-1.329960000	1.195025000	-0.465495000
H	-0.218586000	-0.049109000	1.439907000
H	1.335640000	0.105563000	0.609741000
C	0.837576000	-2.424880000	0.339182000
H	0.595132000	-2.518051000	1.407273000
H	0.650721000	-3.383789000	-0.157347000
H	1.905522000	-2.184498000	0.241619000
C	-1.504191000	-1.716378000	-0.201191000
H	-1.759513000	-1.988762000	0.832891000
H	-2.171804000	-0.902864000	-0.513431000
C	-1.178873000	0.071776000	-3.168513000
H	-1.233639000	0.870246000	-3.922373000
H	-1.962413000	0.255813000	-2.422055000
C	1.275757000	-0.018309000	-3.660630000
H	2.285972000	-0.102539000	-3.236256000
H	1.097754000	-0.884545000	-4.307723000
H	1.223473000	0.899820000	-4.262719000
N	0.379102000	-1.210125000	-1.728543000
H	-1.683280000	-2.584738000	-0.848519000
H	-1.379414000	-0.889368000	-3.659641000
O	0.395426000	-2.311240000	-2.390121000

CCSD identifier	E	charge	Spin
TEMPO − H	-483.88741884	0	1

C	0.373312000	0.008019000	-2.507760000
C	0.473140000	1.298170000	-1.671767000
C	-0.283342000	1.217194000	-0.350161000
C	0.232066000	0.028699000	0.454432000
C	0.122742000	-1.308430000	-0.302251000
H	0.114743000	2.136953000	-2.288551000
H	1.539395000	1.482583000	-1.461449000
H	-0.144572000	2.147978000	0.221208000
H	-1.367770000	1.127652000	-0.525952000
H	-0.304965000	-0.070553000	1.410609000
H	1.294870000	0.195020000	0.695380000
C	0.943355000	-2.364705000	0.452398000
H	0.592693000	-2.438479000	1.492173000
H	0.850997000	-3.350379000	-0.020237000
H	2.004744000	-2.082882000	0.452132000
C	-1.347220000	-1.779750000	-0.355802000
H	-1.658597000	-2.111062000	0.644924000
H	-2.041153000	-0.994742000	-0.677284000
C	-1.030943000	-0.119492000	-3.137956000
H	-1.136785000	0.620665000	-3.943696000
H	-1.842139000	0.051369000	-2.420964000
C	1.408868000	0.079664000	-3.639515000
H	2.421713000	0.115724000	-3.216627000
H	1.342804000	-0.797501000	-4.295174000
H	1.240192000	0.983900000	-4.242200000
N	0.784428000	-1.117765000	-1.623017000
H	-1.474055000	-2.646556000	-1.022614000

H	-1.181402000	-1.106453000	-3.601755000
O	0.766570000	-2.350796000	-2.360025000
H	-0.177192000	-2.534452000	-2.568817000

CCSD identifier	E	charge	Spin
$^{t}Bu_3ArO\cdot$	-424.41180048	0	1/2

C	0.093261000	0.033807000	-2.896791000
C	-1.088397000	-0.251254000	-2.241713000
C	1.276077000	0.360331000	-2.199362000
C	-1.124322000	-0.218085000	-0.776252000
C	1.255227000	0.393090000	-0.785049000
H	2.178362000	0.642916000	-0.253306000
C	0.113075000	0.119557000	-0.063629000
H	0.120141000	0.007052000	-3.990071000
C	0.070458000	0.146630000	1.428573000
H	-0.690154000	0.859482000	1.781214000
H	-0.236546000	-0.832993000	1.825459000
H	1.045652000	0.418189000	1.852487000
C	-2.349619000	-0.597644000	-2.961046000
H	-2.204675000	-0.596440000	-4.048874000
H	-2.717586000	-1.585521000	-2.644746000
H	-3.150301000	0.111568000	-2.701397000
C	2.535596000	0.692385000	-2.940218000
H	2.735925000	1.776559000	-2.900383000
H	3.407476000	0.194432000	-2.490341000
H	2.472930000	0.403908000	-3.997377000
O	-2.183337000	-0.473130000	-0.148881000

CCSD identifier	E	charge	Spin
$^{t}Bu_3ArOH$	-425.04314378	0	1

C	0.187806000	0.032675000	-2.940992000
C	-1.030149000	-0.134845000	-2.280682000
C	1.377503000	0.324719000	-2.260626000
C	-1.044484000	-0.003111000	-0.881844000
C	1.318828000	0.444453000	-0.870434000
H	2.231962000	0.668545000	-0.311908000
C	0.123308000	0.285617000	-0.160157000
H	0.204742000	-0.070202000	-4.029847000
C	0.075099000	0.414136000	1.339098000
H	-0.594002000	1.229394000	1.665613000
H	-0.272353000	-0.514735000	1.824435000
H	1.071447000	0.634140000	1.742435000
C	-2.301500000	-0.449576000	-3.016607000
H	-2.118732000	-0.521256000	-4.096498000
H	-2.737152000	-1.399611000	-2.671711000
H	-3.065432000	0.323272000	-2.841877000
C	2.670814000	0.513915000	-3.009928000

H	3.520301000	0.604634000	-2.319561000
H	2.873115000	-0.331525000	-3.684676000
H	2.646831000	1.423718000	-3.630326000
O	-2.260642000	-0.174673000	-0.255252000
H	-2.126139000	-0.056466000	0.701732000
